# The impact of midwife workload on delivery of care, and mother and baby outcomes in maternity settings in OECD countries: A systematic review

**DOI:** 10.1371/journal.pone.0329117

**Published:** 2025-08-25

**Authors:** Richard Mattock, Chris Bojke, Judy Wright, Tomasina Stacey

**Affiliations:** 1 Academic Unit of Health Economics, Leeds Institute of Health Sciences, University of Leeds, Leeds, United Kingdom; 2 Lumanity Inc, Sheffield, United Kingdom; 3 Florence Nightingale Faculty of Nursing, Midwifery & Palliative Care, King’s College London, London, United Kingdom; Centre of Postgraduate Medical Education, POLAND

## Abstract

**Background:**

Excessive midwifery workload is a growing concern that may affect safety and quality of care, with potential consequences for mothers and babies.

**Aims:**

To assess how midwife workload affects delivery of care, and maternal and neonatal outcomes; and whether maternal, neonatal, and staffing factors modify these relationships.

**Methods:**

This systematic review updated a prior review (January 1998 to June 2014) with revisions to search strategies. We searched for new evidence (June 2014 to October 2023) across 11 academic databases (Cochrane Database of Systematic Reviews (Wiley); Cochrane Central Register of Controlled Trials (Wiley); CINAHL (EBSCOhost); EconLit (EBSCOhost; Embase (Ovid); Epistemonikos; Health Management Information Consortium (Ovid); International HTA Database (INAHTA); Maternity & Infant Care Database (Ovid); Ovid MEDLINE(R); CEA Registry) and 10 grey literature websites. Screening involved multiple reviewers, with 10% of records independently double-screened. Inclusion criteria were intrapartum births in maternity wards in OECD countries, a measure of midwifery workload, and outcomes related to provision of care, mode of birth, and maternal or neonatal morbidity and mortality. A single reviewer conducted data extraction, bias assessments, and a narrative synthesis.

**Results:**

We included 23 studies (15 new, 8 from the original review) from the UK, USA, Italy, France, and Germany, covering 2,943,120 births. Only three studies were rated as high quality. Many outcomes showed no significant effects, or inconsistent effects across studies. High workload was significantly linked to care delays, increased instrumental and caesarean births, and some maternal outcomes (e.g., perineal trauma). Associations were modified by maternal characteristics, including clinical risk, parity, and civil status. No significant associations were found between workload and neonatal outcomes, except for one low-quality study reporting increased neonatal ward admissions.

**Conclusions:**

High midwifery workload may alter care provision, potentially affecting mother and baby outcomes. Further robust research is needed to address limitations in current evidence.

## Introduction

Maternity services are the single most common reason for admission to hospital [1]. Arguably, the most important part of the maternal care pathway is during the intrapartum period, where underlying mortality rates for babies on the day of birth exceed that of any other average day throughout their life [2]. The availability of maternity services differs across settings and can include both obstetric or consultant led, and midwife led birthing units. Midwifery led units are often preferred by mothers for uncomplicated pregnancies as they offer a more comfortable, homely and less clinical environment [3]. There is also evidence that midwifery led units are associated with reduced intervention rates and better outcomes [4].

A key challenge for maternity services is managing the demand for care given the available supply of maternity staff. For instance, UK quality standards indicate that women in labour should receive one-to-one continuous care from an assigned midwife [5]. However, to reach safe staffing requirements, the Royal College for Midwives estimates there to be a current shortfall of 2,000 FTE midwives, a figure supported by estimates from UK government ministers [6].

Multiple inquiries have highlighted unsafe midwifery staffing levels as a key factor contributing to failures in maternity and neonatal services [7–9]. Despite these findings, there remains limited quantitative evidence definitively linking higher midwifery workload to negative outcomes for mothers and babies. Bazian [10] conducted a systematic literature review to inform the National Institute for Health and Care Excellence (NICE) guidelines on safe midwifery staffing [11]: Across eight UK based studies, some significant positive associations were identified between high midwife workload and increased maternal readmission, time to admission for emergency caesarean birth, and decreased likelihood of birth with bodily integrity (i.e., no uterine damage, perineal tear, stitches or episiotomy). However, in general relationships were inconsistent, and there was no evidence of an impact on overall caesarean birth rates, or on maternal and neonatal morbidity and mortality.

Additionally, the NICE guidelines [11] state limitations in the literature identified by Bazian [10], mainly related to the use of aggregated or annual data to measure midwifery staffing levels and/or the demand for midwifery care (i.e., the number of births per ward). Annual data doesn’t identify real time fluctuations in the supply and demand for midwives which could lead to measurement error bias. A maternity ward could be classified as being appropriately staffed throughout the year but may have days within the year of substantial understaffing that are most strongly associated with negative outcomes. Further, it is unclear how maternal demographic characteristics, case complexity, staffing conditions, and factors like staff skill mix influence the impact of low staffing levels on outcomes. Understanding how these factors contribute to varying staffing requirements across different settings is important for improving care delivery and outcomes.

The purpose of the current study was to update the review by Bazian [10], incorporating new evidence on the relationship between midwifery staffing and delivery of care, and maternal and baby outcomes from all Organisation for Economic Cooperation and Development (OECD) countries. We updated three of the research questions (RQ) specified in the original review [10]:

RQ1: What delivery of care, maternal and neonatal activities and outcomes are associated with midwifery staffing in maternity wards/units or hospitals?

RQ2: What provision of care, and maternal and neonatal factors affect midwifery staffing requirements in maternity wards/units or hospitals?

RQ3: What staffing factors affect safe midwifery staffing requirements in maternity wards/units or hospitals?

The objective of RQ2 and RQ3 was to identify potential modifiers of the relationship between midwifery workload and outcomes. Relevant studies would examine how maternal, neonatal, and staffing factors influence this relationship, for example, through subgroup analyses or interaction tests with staffing variables.

## Materials and methods

### Systematic review methods

This review update involved conducting new database searches, screening newly identified studies, and extracting relevant data, while the synthesis integrates findings from both the original Bazian review [10] and the updated search. Methods for the update generally followed those in the original review by Bazian [10], but made some adaptations including to the search strategy, the data extraction forms, and the risk of bias assessment tool. We followed guidance on systematic review methods for studies of etiology and risk in Chapter 7 of the JBI Manual for Evidence Synthesis [12], and PRISMA reporting guidelines [13]. We did not register a protocol as this was a review update.

### Inclusion and exclusion criteria

The inclusion criteria for this systematic review mirrored that used in the original review by Bazian [10]. We included primary research studies that examined the relationship between midwifery staffing and delivery of care, and maternal and neonatal outcomes.

The population of interest was women giving birth in maternity wards in OECD countries. The exposure of interest was midwifery staffing levels on maternity wards, for example a measure of midwife supply (e.g., number of midwives) and demand for care (e.g., number of births). We included outcomes occurring during the intrapartum period or immediately following birth related to: delivery of care including any measure of midwifery activity, or description of treatment provided by midwives; mode of birth for example rate of caesarean birth or instrumental birth; maternal outcomes including mortality, adverse events, e.g., haemorrhage, perineal tear, and post-birth length of stay; and neonatal outcomes including mortality, transfer to intensive neonatal care unit, and APGAR score. We excluded pregnancy related outcomes, e.g., preterm birth and still birth and postnatal outcomes, e.g., breastfeeding rates.

For RQ2 the included modifiers related to maternal and neonatal factors, for example clinical risk. For RQ3 the included modifiers related to staffing factors, for example midwifery skill mix and the availability of other maternity care staff.

All research questions focused on studies conducted in maternity wards/units or hospitals serving local communities, aligning with the emphasis on local midwifery settings as outlined in the Bazian review, who use terminology specific to the UK context. Consequently we excluded ecological type studies reporting aggregated regional or national level outcomes. We also excluded systematic reviews and meta-analyses, non-English language studies, studies that were not performed in OECD countries, non-primary research studies (e.g., editorials, summary articles, and press releases), studies about maternity workforce planning at regional or national levels, studies about optimal service delivery models, purely qualitative studies, and studies not relevant to the research question.

### Search strategy

Our searches identified publications between June 2014 and October 2023. Two sets of searches were conducted, originally from 21^st^ to 23^rd^ of February 2022, and updated on 3^rd^ to 10^th^ October 2023. Searches were based on the NICE [11] guideline [NG4] Evidence review 2 [10] and Evidence review 3 [14] searches run in June 2014, were conducted by an Information Specialist [JW], and peer-reviewed by a second Information Specialist using the PRESS checklist [15]. The searches replicated the original June 2014 searches where possible. Minor changes made are outlined below. The full search strategies are detailed for each source in supplementary material S2.

Searches were designed for the concepts: Midwives or Maternity workers, staffing or workload. New subject headings were included in the searches. The MeSHs ‘Manpower’ and ‘Health Manpower’ have been re-named ‘Workforce’ and ‘Health workforce’, and there is no longer a manpower floating sub-heading so this was removed from the Medline search. Additional headings were identified and some headings were changed from a single to an exploded term in MEDLINE and Cochrane (Burnout, Professional/, Occupational Stress/, Work Schedule Tolerance/, exp animals/), EMBASE (professional burnout/, Job stress/, exp shift worker/, exp personnel shortage/, exp human/), HMIC (workforce/ or workforce planning/) CINAHL (Workforce/, nursing manpower/, stress, occupational/).

Following testing, minor changes were made to the free text word searches to improve precision without compromising sensitivity. Truncation was added to ‘skill’ in the phrase “skill? mix*” to identify skill and skills mix. The search for midwi*.tw was edited to (midwif* or midwiv*) to remove irrelevant results. The redundant search lines for midwi* adj (near to) worker search terms were removed to simplify the search.

The NICE OECD countries’ geographic search filters [16] were used to limit searches to OECD countries in MEDLINE and EMBASE to align with the review’s inclusion criteria. The search filters were adapted for use in CINAHL and Maternity & Infant Care Database. All searches limited studies and reports to those published from January 2014 onwards.

### Information sources

We searched the following academic databases: Cochrane Database of Systematic Reviews; Cochrane Central Register of Controlled Trials; CINAHL (EBSCOhost); EconLit (EBSCOhost); Embase Classic+Embase (Ovid); Epistemonikos (https://www.epistemonikos.org/); HMIC Health Management Information Consortium (Ovid); International HTA Database (INAHTA) (https://database.inahta.org/); Maternity & Infant Care Database (MIDIRS); Ovid MEDLINE(R) ALL; and the CEA Registry (https://cevr.tuftsmedicalcenter.org/databases/cea-registry/).

Unpublished (grey) literature was retrieved from the following sources: the King’s Fund; the Royal College of Midwives; the Royal College of Paediatrics and Child Health; the Department of Health; NHS England; NHS Scotland; the Welsh Government Statistics and Research Scottish Government; NICE Evidence; and Google Scholar.

Further relevant studies were sought by citation searching (forwards and backwards) the included studies. This included a scoping review on a related topic by Turner et al. (2021) [17]. We were also provided with four potentially relevant studies from peer reviewers. Search results were managed in an EndNote library where duplicates were removed automatically and manually using University of Leeds AUHE guidance [18].

### Screening

Search results (with duplicates removed) were transferred to Rayyan.ai software, which was used for study screening. The title and abstract screening for the main search was conducted by three reviewers (RM, CB, and JW), while the updated search was screened by two reviewers (RM, JW). Ten percent of records were screened independently by all reviewers to ensure a consistent approach and reduce risk of bias, with any conflicts resolved through discussions between all reviewers. Full-text screening was conducted independently by two reviewers. One reviewer (RM) screened 100% of the records, while the other reviewer (CB) screened 50%. Any uncertainties were discussed with a clinical specialist (TS) before being included or excluded.

### Data extraction

Data extraction was conducted by a single reviewer (RM), using pre-designed data extraction forms that were piloted prior to implementation. The items extracted for RQ1 were: study characteristics (location, type of maternity ward, number in sample, population inclusion/exclusion criteria, study design, and analytical methods); measurement units for midwifery staffing (e.g., number on staff on wards), service demand (e.g., total births on ward) and workload (e.g., ratio of midwives to births) including the time these measures were taken in relation to births; a list of any primary and secondary outcomes that fit into categories of delivery of care, mode of birth, and maternal and neonatal morbidity and mortality; a list of all control variables; quantitative findings (e.g., regression coefficients and statistical significance); and any author’s interpretations.

For RQ2 and RQ3 additional data were extracted for: study design including methods for analysing impacts of modifying factors; quantitative findings across modifying factor categories (e.g., regression coefficients for different sub-groups/interactions); a list of control variables that are associated/not associated with the outcomes of interest and the midwifery staffing variable; and any author’s interpretations related to the modifying factors.

In some cases the data extracted for the midwifery workload variable were modified to ensure that all associations with outcomes were reported on a consistent scale, where increasingly positive values indicate higher workload (i.e., a higher ratio of births to midwives).

### Risk of bias assessment

Risk of bias assessments were conducted as part of the data extraction by a single reviewer (RM) using the National Heart Lung and Blood Institute (NHLBI) study quality assessment tool for observational cohort and cross sectional studies [19]. This tool was selected due to specific focus on exposure measures allowing us to assess study quality in relation to the midwifery workload measure, a key area where the current evidence is limited [14]. Assessments were made by designating the exposure as the midwifery staffing variable, irrespective of whether this was the primary objective/aim of the study. Consequently, some studies of generally good quality could be categorised as poor quality due to them being less relevant or not reporting detailed outcomes for the midwifery staffing questions posed in this review.

### Synthesis methods

Prior to synthesis, we merged the studies included in the original Bazian review with those identified through the updated search. A narrative synthesis was then conducted by RM with input from CB and TS. The synthesis included a full description of each study and a tabulated summary of quantitative results for the midwifery workload measures on all reported outcomes as direction of effect and statistical significance. A narrative summary of findings was conducted after grouping studies by outcome measure (delivery of care, maternal, neonatal) and inclusion of moderating factors relating to mothers or babies (RQ2) and staff (RQ3).

Due to the nature of the review questions, the included studies were expected to use heterogeneous methodologies, with potentially different measures for midwifery workload, and reported impacts on multiple differing outcomes. Therefore, it was not appropriate to conduct any meta-analysis or other quantitative synthesis.

## Results

### Study selection

After de-duplication, the search identified 7,394 records from databases and 817 records from websites. An additional 6 records were identified through prior knowledge of the literature and citation searching, which included a scoping review found during the search and recommended citations provided during peer review. In total 8,217 records were screened on title and abstract. A total of 138 records passed the first phase of screening. The full text was obtained for 130 studies.

The second phase of screening excluded 115 records after reading the full text. Reasons for exclusion were: wrong study design, i.e., not containing a primary research study (n = 30); being the wrong publication type, e.g., editorials and commentaries (n = 32); not including variables related to midwifery staffing (n = 33); wrong outcome, e.g., post-natal outcomes, or no outcome (n = 11); wrong setting, e.g., reporting aggregated national outcomes (n = 4); and not published in English language (n = 1). Due to an overlap of search dates, four studies were identified that had already been included in the NICE safe midwifery staffing guideline, including the guideline itself [11]. A full list of the excluded studies is provided in supplementary material S3.

**Fig 1:**
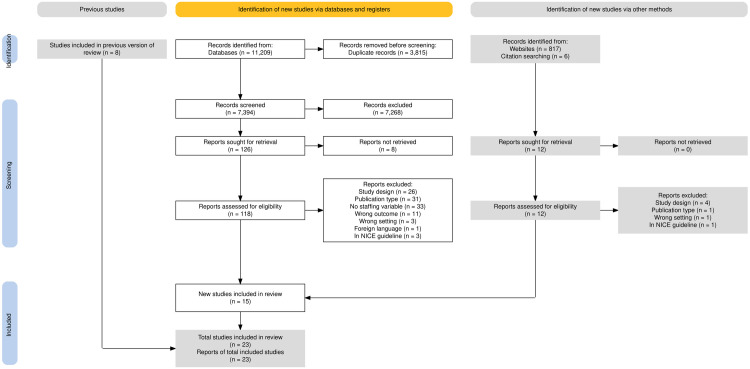
PRISMA flow diagram.

A total of 15 studies were identified as relevant to RQ1 [20–34] with 5 also applicable to RQ2 [21–23,26,34], and 3 to RQ3 [26,28,34]. These were combined with 8 studies [35–42] from the original Bazian [10] review for the synthesis, resulting in 23 studies for RQ1, 12 studies for RQ2 [21–23,26,34–40,42], and 8 studies for RQ3 [26,28,34–38,40]. The study selection process is detailed in the PRISMA flow diagram in Fig 1.

### Narrative synthesis

#### Overview of included studies.

***Study characteristics:*** The full data extraction tables for the updated search are reported in supplementary material S4 and S5 for RQ1 and RQ2/RQ3 respectively, including a risk of bias assessment. A full summary of each study is provided in supplementary material S6 Data extraction tables and risk of bias assessments for the studies included in the original review are provided in Bazian [10].

In summary, 23 studies were included, with 13 from the UK [22,23,27,30,31,35–42], four from the USA [28,29,32,33], three from France [24,26,34], two from Italy [20,21], and one from Germany [25]. The studies covered 2,943,120 births. The study methods were typically cross-sectional [24,26,28–33,36–40] or cohort [20–23,34,35,42], with two randomised control trials (RCT) [25,41] and an enquiry [27] also included.

***Measurement of midwifery staffing:*** In 19 of the 23 included studies [21–26,29–40,42] midwifery workload was measured using both supply (number of midwives) and demand (number of births). This was typically expressed as a ratio (e.g., midwives per number of births) or a dichotomous variable (e.g., understaffed yes/no) based on arbitrary thresholds like less than one midwife per birth.

Only 7 studies measured workload at the same time/or day as births. Robertson et al. (2021) [30] used the Birthrate Plus® tool to calculate the real-time number of midwives required on a labour ward given the number of births and case-mix. Five studies relied on data recorded by staff, for example by shift leaders on birthing units or through midwife self-report [22,25,35,39,42]. Meanwhile, Facchini (2022) [21] did not observe daily staff ratios directly, instead assuming the number of midwives on duty was equivalent to the number of staff scheduled to be on the ward.

Ten studies used aggregated workload data, either over time (e.g., annually) or across hospitals/trusts. This included studies using aggregate measures for births [24], and both births and midwives [26,31,32,34,36–38,40]. Hollowell et al. (2015) [23] obtained the number of midwives and births on the ward each day but aggregated this to a trust level “understaffing” variable identifying the percentage of times that births exceeded staff on duty per quarter or per year.

Four studies inferred workload rather than measuring it directly: One assumed workload differences based on maternity unit type [20], another reported workload within broader staffing/capacity issues [27], a third relied on midwives’ recall via email survey [28], and one incorporated staffing within the differences between caseload and shared care models [41]. In two studies [29,33] methods to derive workload measures were not clearly reported.

***Quality of evidence:*** Only 3 studies [21,22,40] were categorised as “good” quality with low risk of bias. Ten studies [25,26,32,33,36–39,41,42] were categorised as “fair” quality, but were limited due to: the staffing variable being an aggregate measure [26,32,36–38]; use of an arbitrary cut-off value to identify high workload [32]; regression equations not including covariates such as impact of other healthcare staffing levels [26]; level of reporting [25,33]; missing data [39]; and inability to differentiate midwifery workload effects with model of care effects [41].

The remaining 10 studies were categorised as “poor” quality with high risk of bias. Reasons for poor quality rating were: reporting associations without adjusting for control variables [23,24,30,35]; using a before and after study design without a counterfactual [27]; not directly measuring midwifery workload [20]; relying on subjective measures of workload [28]; use of maternal self-reported outcome measures [31]; potential issues with interpretation for the regression equations as midwifery staffing levels were used to derive two variables in the same equation [34]; and level of reporting [29].

***Outcomes:*** The 23 studies examined a range of outcomes related to delivery of care, mode of birth, maternal health, and neonatal health (Tables 1–4). Care delivery outcomes included epidural use, intrapartum transfers, oxytocin administration, analgesia, and delays in emergency caesarean births, transfers, and induction. Mode of birth outcomes covered intrapartum caesarean births, operative birth, augmentation, and spontaneous vaginal birth. Maternal outcomes included hospital length of stay, perineal outcomes, postpartum haemorrhage, and a composite measure of maternal health. Neonatal outcomes included gestation length, low birth weight, neonatal ward admission, Apgar scores, neonatal, skin-to-skin contact, and a perinatal morbidity/mortality composite.

**Table 1:** Association between midwife workload and delivery of care outcomes.

Outcome	Study	Statistical significance	Direction of effect
Epidural	Freeman (2017) [22]	***	–
	Kpéa (2015) [26]	*	+
	NSCCRT (2000) [41]	**	–
	Joyce (2002) [37]	NS	N/A
	Rowe (2014) [39]	NS	N/A
Intrapartum transfer/ referral	Freeman (2017) [22]	NS	N/A
	Hollowell (2015) [23]	NS	N/A
Oxytocin dose	Isidore (2018) [24]	***	+
Analgesia	Facchini (2022) [21]	NS	N/A
Decision to emergency caesarean birth < 30 minutes	Cerbinskaite (2011) [35]	***	–
Transfer time < 15 minutes	Cerbinskaite (2011) [35]	***	–
Delay to induction	Robertson (2021) [30]	NS	N/A
	Draper (2017) [27]	N/A	–
Likelihood of induction	Wilson (2021) [33]	**	–
Attended by known midwife	NSCCRT (2000) [41]	***	–

Statistical significance: * = p < 0.05; ** = p < 0.01; *** = p < 0.001; NS = not statistically significant; N/A = no statistical test conducted. Direction of effect: negative (-) values indicate outcome decreases with increasing midwife workload; positive (+) values indicate outcome increases with increasing midwife workload.

**Table 2:** Association between midwife workload and mode of birth outcomes.

Outcome	Study	Statistical significance	Direction of effect
Intrapartum caesarean birth	Facchini (2022) [21]	*	+
	Vanderlaan (2023) [32]	***	+
	Zbiri (2018) [34]	NS	N/A
	Hollowell (2015) [23]	* [Nulliparous, OU]	–
		NS [All other]	N/A
	Wilson (2021) [33]	NS	N/A
	Sandall (2014) [40]	NS	N/A
	Rowe (2014) [39]	NS	N/A
	Joyce (2002) [37]	NS	N/A
	NSCCRT (2000) [41]	NS	N/A
Operative birth	Facchini (2022) [21]	NS	N/A
	Hollowell (2015) [23]	NS	N/A
Intrapartum caesarean or operative birth	Knape (2014) [25]	NS	N/A
Normal birth	Hollowell (2015) [23]	NS	N/A
	Sandall (2014) [40]	NS	N/A
	Rowe (2014) [39]	NS	N/A
	NSCCRT (2000) [41]	NS	N/A
Straightforward birth	Rowe (2014) [39]	NS [Nulliparous] ** [Multiparous]	N/A +
Spontaneous vaginal birth	Sandall (2014) [40]	NS	N/A
Instrumental vaginal birth	Rowe (2014) [39]	NS	N/A
	Joyce (2002) [37]	NS	N/A
	NSCCRT (2000) [41]	NS	N/A
Augmentation	Hollowell (2015) [23]	* [Multiparous, OU]	–
		NS [All other analyses]	N/A
	Wilson (2021) [33]	**	–
	Rowe (2014) [39]	*	–
	NSCCRT (2000) [41]	**	+

Statistical significance: * = p < 0.05; ** = p < 0.01; *** = p < 0.001; NS = not statistically significant; N/A = no statistical test conducted. Direction of effect: negative (-) values indicate outcome decreases with increasing midwife workload; positive (+) values indicate outcome increases with increasing midwife workload. OU = obstetric unit.

**Table 3:** Association between midwife workload and maternal outcomes.

Outcome	Study	Statistical significance	Direction of effect
Length of hospital stay	Dani (2020) [20]	NS	N/A
	Freeman (2017) [22]	** [non-complex cases]	–
		NS [complex cases]	N/A
	Facchini (2022) [21]	NS	N/A
	Turner (2022) [31]	*	+
Perineal tear	Freeman (2017) [22]	*** [complex cases]	+
		NS [non-complex cases]	N/A
	NSCCRT (2000) [41]	NS	N/A
Intact perineum	Sandall (2014) [40]	*	–
	NSCCRT (2000) [41]	NS	N/A
Birth with bodily integrity	Sandall (2014) [40]	*	–
Haemorrhage	Facchini (2022) [21]	**	+
	Mercer (2016) [29]	NS	N/A
Maternal readmission to hospital	Gerova (2010) [36]	***	+
Duration of labour < 8 hours	NSCCRT (2000) [41]	***	–
Episiotomy	NSCCRT (2000) [41]	NS	N/A
Healthy mother composite ^A^	Sandall (2014) [40]	NS	N/A

Statistical significance: * = p < 0.05; ** = p < 0.01; *** = p < 0.001; NS = not statistically significant; N/A = no statistical test conducted. Direction of effect: negative (-) values indicate outcome decreases with increasing midwife workload; positive (+) values indicate outcome increases with increasing midwife workload. A: Healthy mother composite comprises birth with bodily integrity, return home in 2 days or less, and no instrumental birth, maternal sepsis, anaesthetic complication, or readmission within 28 days. Complex cases in Freeman (2017) defined if labour onset was spontaneous or required induction.

**Table 4:** Association between midwife workload and neonatal outcomes.

Outcome	Study	Statistical significance	Direction of effect
Admission to neonatal ward	Dani (2020) [20]	***	+
	Facchini (2022) [21]	NS	N/A
	Hollowell (2015) [23]	Not reported	Not reported
	Tucker (2003) [42]	NS	N/A
	NSCCRT (2000) [41]	NS	NA
Healthy baby composite ^A^	Sandall (2014) [40]	NS	N/A
Neonatal resuscitation	Tucker (2003) [42]	NS	N/A
	NSCCRT (2000) [41]	NS	N/A
Time to response to foetal heart trace abnormality	Tucker (2003) [42]	NS	N/A
Continuous Electronic Foetal Monitoring usage	Tucker (2003) [42]	NS	N/A
Apgar score	Freeman (2017) [22]	NS	N/A
	Facchini (2022) [21]	NS	N/A
	Mercer (2016) [29]	NS	N/A
	Tucker (2003) [42]	NS	N/A
Hypoxic Ischaemic Encephalopathy	Mercer (2016) [29]	NS	N/A
Gestation length	NSCCRT (2000) [41]	NS	N/A
Low birth weight	NSCCRT (2000) [41]	NS	N/A
Shoulder dystocia	Mercer (2016) [29]	NS	N/A
Cord pH below 7.0	Mercer (2016) [29]	NS	N/A
Neonatal mortality	Draper (2017) [27]	N/A	+
	Joyce (2004) [38]	NS	N/A
	NSCCRT [41]	NS	N/A
Skin to skin contact	Facchini (2022) [21]	NS	N/A
	Lyndon (2022) [28]	Not reported	–

Statistical significance: * = p < 0.05; ** = p < 0.01; *** = p < 0.001; NS = not statistically significant; N/A = no statistical test conducted. Direction of effect: negative (-) values indicate outcome decreases with increasing midwife workload; positive (+) values indicate outcome increases with increasing midwife workload. A: Healthy baby composite if baby’s weight 2.5 to 4.5 kg, gestational age 37–42 weeks, and live baby.

### Findings

The findings across all 23 studies are summarised in (Tables 1–4). This reports the statistical significance of the associations between midwife workload and outcomes, and the direction of effect for outcomes that are statistically significant.

### Delivery of care

There was some evidence that higher midwifery workload was associated with changes or delays to care. Cerbinskaite et al. (2011) [35] found that when midwife-to-labouring women ratios were at least 1:1, emergency caesarean births were significantly less likely to occur within the recommended 30-minute decision-to-delivery timeframe, and fewer women were transferred to operating theatres within 15 minutes. Similarly, the MBRRACE-UK enquiry into stillbirths and neonatal deaths [27] identified staffing (i.e., maternity units with high workload) as a key factor contributing to delays in transfer for births (10 cases) and induction of labour (4 cases). In contrast, Robertson et al. (2021) [30] did not find significant delays to induction with midwifery staffing shortfall. Isidore and Rousseau (2018) [24] reported a significant association between increased workload and higher oxytocin administration during spontaneous labour. Meanwhile Wilson et al. (2021) [33] identified that higher workload was significantly associated with lower induction rates.

Findings on other delivery of care outcomes were inconsistent. Higher midwife workload was not associated with [37,39], and linked to both decreased [26] and increased [22,41] administration of epidurals. Freeman et al. (2017) [22] found no overall relationship between workload and referrals for obstetrician-led births but noted a significant increase in referral rates for complex cases involving pharmacological induction. Similarly, Hollowell et al. (2015) [23] found no significant effect of workload on transfer rates from planned birth settings to obstetrician-led units during or immediately after labour. Facchini (2022) [21] found no relationship between workload and rates of analgesia.

### Mode of birth

The relationship between midwife workload and caesarean birth rates appeared complex. In a multivariate analysis, Zbiri et al. (2018) [34] found no association between workload and intrapartum caesarean births but reported a significantly higher likelihood of elective caesarean births at higher workload. Facchini (2022) [21] observed a 19% increase in caesarean births when comparing the highest to lowest workload 20th percentiles; however, no significant association was found when workload was measured as a continuous variable, suggesting effects may be limited to workload extremes. Consistent with this, Vanderlaan (2023) [32] found that counties with the highest 10% of midwife to birth ratios (> 4.5 midwives per 1,000 births) had significantly lower caesarean birth rates compared to the 80% of counties with lower midwife densities (1 to 4.5 midwives per 1,000 births). Meanwhile, Wilson (2021) [33] identified a positive association between caesarean birth rates and a quadratic term for midwifery staffing, suggesting that there may be an optimal staffing range where too few or too many nursing hours per birth could increase the likelihood of a caesarean birth.

Several other studies using simple linear measures of midwifery staffing [23,37,39–41] found no significant associations with caesarean birth rates. Meanwhile, Knape et al. (2014) [25] found that midwifery workload was significantly associated with mode of birth (caesarean birth or operative birth) in unadjusted bivariate analyses, but this association was not significant and therefore removed from the adjusted multivariate logistic model.

Across other mode of birth outcomes, three studies found that higher workload correlated with significantly reduced augmentation rates [23,33,39]. There was no consistent evidence of associations between midwife workload and normal/spontaneous births, operative vaginal births, and overall intervention rates [21,23,39–41].

### Maternal outcomes

Seven studies examined the impact of midwifery workload on maternal outcomes, with mixed findings. Higher workload was associated with an increased risk of perineal tears [22], and a small but significant decrease in birth with bodily integrity [40], including an intact perineum [21]. Midwife workload was also associated with postpartum haemorrhage [21], though this relationship disappeared when adjusting for caesarean birth rates, suggesting the effect was largely mediated by surgical births.

Findings on post-birth length of stay were inconsistent. Freeman et al. (2017) [22] reported an 8% reduction in hospital stay during higher workload, potentially due to fewer epidurals and pressures to discharge earlier. In contrast, Turner (2022) [31] found that higher staffing levels were associated with fewer delays in discharge using maternal self-reported data. Other studies [20,21] found no significant relationship between workload and length of stay. But, higher staffing levels were significantly linked to a lower risk of maternal readmission within 28 days [36].

### Neonatal outcomes

Eleven studies examined the impact of midwifery workload on neonatal outcomes [20–23,27–29,38,40–42]. Overall, higher midwife workload was not found to significantly increase the risk of poor neonatal outcomes, including Apgar scores, a composite “healthy baby” outcome, gestation length, low birth weight, shoulder dystocia, cord pH, neonatal resuscitation rates, response time to foetal heart trace abnormalities, or continuous electronic foetal monitoring (CEFM) usage. Dani et al. (2020) [20] found a significant association between higher midwifery workload and increased neonatal ward admissions, but this was not supported by four other studies [21,23,41,42], which found no significant relationship.

### Maternal and neonatal modifying factors (RQ2)

Studies examining how maternal and neonatal characteristics influence the impact of midwifery workload produced mixed findings. While some studies found that midwifery workload effects varied by maternal risk factors, parity, or case complexity, others reported no clear patterns.

Two studies conducted formal interaction analyses. Facchini (2022) [21] found that at low workload levels, caesarean birth rates were similar for single and married mothers (8.9% vs. 8.6%), but at high workload (90th+ percentile), single mothers were 42% more likely to undergo a caesarean (13.8% vs. 9.9%). Sandall et al. (2014) [40] found lower workload had greater benefits for low-risk women in “healthy baby” (p < 0.009) and “healthy mother” (p < 0.001) composite outcomes but did not significantly affect mode of birth or perineal outcomes. The benefits of reduced workload were also significantly more pronounced for women with four or more children for the intact perineum outcome.

Five studies examined maternal and neonatal subgroups without formal interaction testing. Freeman et al. (2017) [22] found that higher workload significantly reduced epidural rates in non-complex cases, but not in complex ones. In contrast, referrals to obstetric-led birth and perineal tears increased for complex cases only. Hollowell et al. (2015) [23] found no clear workload patterns across different maternity units and parity, except for significant associations with caesarean births in nulliparous women and straightforward birth and augmentation in multiparous women in obstetric units. Cerbinskaite et al. (2011) [35] found the benefits of lower workload on decision-to-birth time were greater for non-life-threatening emergency caesareans, likely due to prioritisation of urgent cases regardless of workload. Tucker et al. (2003) [42] reported that higher workload was linked to lower CEFM use in low-risk women but higher use in high-risk women, though neither association was significant. Rowe et al. (2014) [39] found that lower workload reduced straightforward births and augmentation for multiparous women, whilst workload increased intrapartum caesarean births only in nulliparous women.

Eight studies included maternal and neonatal factors as covariates but did not test interactions or conduct subgroup analyses. Maternal characteristics (age, parity, BMI) and pregnancy factors (weight, gestation) were consistently associated with outcomes. Demographic factors (e.g., deprivation, education) showed inconsistent relationships, while medical history and risk factors influenced caesarean rates [26] but not care delivery outcomes like oxytocin administration [34].

### Staffing modifying factors (RQ3)

No studies directly examined how staffing factors modified the relationship between midwifery workload and outcomes, but seven studies included staffing as covariates. While staff mix and workload were generally associated with outcomes, individual staff characteristics were not.

Kpéa et al. (2015) [26] found anaesthetist availability significantly influenced oxytocin use, while Zbiri et al. (2018) [34] reported that obstetrician workload, but not midwife workload, was strongly linked to higher non-elective caesarean rates. Gerova et al. (2010) [36] found a higher consultant-to-midwife ratio reduced maternal readmission risk, whereas a higher registered nurse-to-midwife ratio increased it. Sandall et al. (2014) [40] found no significant impact of staff mix ratios on outcomes such as caesarean births or maternal and neonatal health composites. Joyce et al. (2002) [37] linked higher consultant and junior obstetrics and gynaecology (O&G) staffing to increased caesarean births, while more consultant anaesthetist sessions and junior O&G staffing were associated with greater epidural use. Joyce et al. (2004) [38] found increasing consultant O&G rates were linked to lower birth weight.

## Discussion

### Main findings

While there are an increasing number of studies on midwifery staffing, the impact of high workload on delivery of care, and maternal and neonatal outcomes is unclear. Of the 23 studies included in this review, only 3 were rated as high quality. Limitations in midwifery staffing measurements and reliance on cross-sectional study designs were common. Many outcomes showed no significant effects, and where effects were found, the direction often varied across studies.

Significant associations were observed between high workload and delays in care as well as an increased likelihood of instrumental or caesarean births, with some effects on maternal outcomes – particularly perineal trauma. However, there was no evidence of consistent significant impacts on long term maternal or neonatal outcomes (i.e., mortality or severe morbidity). This may suggest that midwives effectively prioritise care during higher workload to mitigate the worst outcomes. One low-quality study [20] did report significant differences between midwife-led and obstetric-led units regarding neonatal ward admissions. However, these differences are more likely to be attributed to variations in ward-level characteristics, such as maternal demographics and risk profiles, rather than differences in midwifery staffing levels.

### Changes to delivery of care

Findings from two good quality studies suggest that midwives may change their delivery of care to account for increased demand. Freeman et al. (2017) [22] propose that midwives may use two levers to manage workload. Firstly reducing high resource discretionary care (i.e., epidurals) for non-complex cases, and secondly increasing transfer rates to obstetric-led birth but only for complex cases. Additionally, Freeman et al. (2017) [22] suggests that obstetricians may be more amenable to taking on additional cases when midwife workload is high. Consistent with this, Facchini (2022) [21] hypothesize that optimal demand management could lead to an increase in mothers who are transferred from midwives to obstetricians during high workload scenarios. However, these findings are not consistently supported by evidence from the other lower quality included studies, and in some cases significant effects were identified in the opposite direction [23,26].

Any impacts of midwife workload on maternal and neonatal outcomes are likely to occur through delivery of care outcomes. Increased post-partum haemorrhage was driven almost entirely through the increase in caesarean birth [21]. Freeman et al. (2017) [22] also found a direct impact on maternal outcomes following changes to delivery of care, but these effects were not straightforward with some being surprisingly beneficial. For example, epidurals can be unnecessary in low risk births and typically result in increased hospital length of stay, therefore increased rationing for the least complex cases may reduce costs and improve maternal outcomes [22]. Similarly, increasing transfer rates to obstetric led birth for the most complex cases could be beneficial if this group require a higher level of care. On the other hand, it may be that obstetricians are more likely to perform (potentially) unnecessary instrumental or caesarean births as a method to reduce birth times and manage higher demand.

High midwifery workload may also lead to missed or delayed essential care, increasing risk and delaying timely management. In a study across nine countries, missed nursing care has been found to mediate the relationship between general nurse staffing and the risk of 30-day mortality in multiple clinical settings [43]. Among the midwifery studies included in this review, one low-quality [35] and one fair-quality study [41] identified significant associations between high workload and missed/delayed care, such as extended transfer times (over 15 minutes), delayed emergency caesarean birth decisions (over 30 minutes), and reduced continuity of care.

Missed midwifery care during periods of high workload may also impact postnatal outcomes. Whilst not included as outcomes in this review, three of the included studies examined exclusive breastfeeding rates, with two finding significant negative associations with increasing midwifery workload [20,21,28]. Lyndon et al. (2022) [28] used a formal mediation analysis with nursing survey data and found that missed care items (skin-to-skin contact and breastfeeding within the first hour) significantly mediated the relationship between workload and exclusive breastfeeding rates.

### Generalisability of findings

Seven of the included studies directly examined factors that could modify the impact of midwifery workload on outcomes. Consistently, staffing effects significantly varied based on maternal characteristics such as clinical risk, case complexity, parity, and marital status. Additionally, midwives’ ability to manage demand appears to depend on case complexity and maternal demographic characteristics, which differ across settings [21,22]. Whilst no studies directly explored how staffing factors modify workload effects, it is likely that skill-mix of other staff on the ward plays a significant role. For example, the impact of high midwifery workload on transfer rates may depend on the availability of obstetricians. Supporting this, Zbiri et al (2018) [34] found that intrapartum caesarean birth increased with greater availability of FTE obstetricians.

These findings have implications for generalisability. First, differences in maternal characteristics across OECD settings may explain some inconsistencies between studies, particularly as several did not adequately adjust for covariates. Second, our results may not be applicable to non-OECD settings, where maternal demographics, staffing levels, and hospital resources differ significantly. For instance, while we found no evidence that workload affected maternal or neonatal mortality, a global ecological study of low- and middle-income countries found that a higher number of midwives per population was associated with lower maternal and neonatal mortality rates. This effect was strongest in lower-income countries, weaker in lower-middle-income countries, and absent in upper-middle-income countries [44].

### Midwifery workload variable specification

Inconsistencies in the evidence may also be partly explained due to study design limitations. Ten of the included studies were rated as poor quality with a high potential for bias whilst eight relied on aggregated staffing or birth data (e.g., annual measures), introducing potential measurement error. A key priority for future research is appropriately measuring midwifery workload.

It may be that high workload is only detrimental in the most extreme cases or reaches a tipping point at an unsafe level. This would explain findings by Facchini (2022) [21] where a continuous measure of workload was not significantly associated with outcomes, but a binary variable using the highest and lowest 20^th^ percentiles was. Similarly Wilson et al. (2021) [33] did not identify a significant impact of workload on likelihood of caesarean birth using a simple linear regression specification for workload, but found a positive association for a quadratic term which differed across optimal staffing scenarios.

Future research should focus on estimating midwifery staffing levels and service demand on the day of birth to reduce measurement error bias. Such evidence may become more widely available through digital tools, for example Birthrate Plus®, which helps midwives plan required staffing levels using real time data on birth number and complexity [45]. One study in this review utilised the Birthrate Plus® but was limited due to other study design factors [30].

### Limitations

This review updated a previous review [10] and involved methodologies conducted separately by different research teams. While we integrated studies from both the updated search and the original review in our narrative synthesis, due to resource constraints we did not re-extract data or reassess the risk of bias for the original studies. Since different researchers used slightly different tools in the original review, some inconsistencies in methodology may be present.

One study [41] from the original review was an RCT comparing different models of care (caseload versus standard “shared” care). While it reported differences in midwife-to-patient ratios between trial arms, it was not possible to determine whether the observed outcomes were due to workload or the care model itself. For instance, continuity of care models such as caseload care may place additional demands on midwives, given broader responsibilities across antenatal, intrapartum, and postnatal care. Therefore, we did not include similar RCTs comparing care models from the updated search.

Additionally, during screening for the updated review, a high volume of search results meant it was not possible for multiple reviewers to screen all title and abstracts independently. Further, because of the heterogeneity of maternity services across settings we limited the inclusion criteria to OECD countries, which may have excluded some relevant evidence from non-OECD countries. We also did not formally assess the risk of bias due to missing results (i.e., reporting bias) or conduct a certainty assessment. Finally, the original review [10] was published as part of a National Institute for Health and Care Excellence (NICE) guideline [11] rather than in a peer-reviewed journal, which may influence its perceived rigor and reliability.

## Conclusions

We found some evidence that midwives may modify or delay care during high workload scenarios which could impact on birth modes and maternal outcomes directly related to birth. There was no consistent evidence of a negative impact on maternal and neonatal morbidity or mortality. However, given limitations related to study quality in the current evidence base, this does not mean that midwifery workload has no consequences for women and babies. It may be that midwives effectively prioritise and maintain safe care during high workload periods, but such practices may not be sustainable over the long term. Future research should explore how workload is associated with midwife mental health and wellbeing outcomes, and whether more difficult and stressful working conditions have an impact on staff retention rates.

## Supporting information

S1 File Included studies reference list. (DOCX)

S2 File Search STRATEGIES. (DOCX)

S3 File Excluded studies table. (DOCX)

S4 File Data extraction & risk of bias assessments RQ1. (DOCX)

S5 File Data extraction tables RQ2 & RQ3. (DOCX)

S6 File Summary of included studies. (DOCX)

S7 File PRISMA-P checklist. (DOCX)
