# Supplementary material for: The impact of midwife workload on delivery of care, and mother and baby outcomes in maternity settings in OECD countries: A systematic review
Source: PLoS One. 2025 Aug 25;20(8):e0329117. doi: 10.1371/journal.pone.0329117 (PMC12377604; doi:10.1371/journal.pone.0329117)
Supplement: S1 File — (DOCX) [file pone.0329117.s001.docx]

# Supporting Information S1. Included Studies Reference List

## Updated Search

Dani, C., Papini, S., Iannuzzi, L., & Pratesi, S. (2020). Midwife‐to‐newborn ratio and neonatal outcome in healthy term infants. Acta Paediatrica, 109(9), 1787-1790.

Draper, E. S., Kurinczuk, J. J., & Kenyon, S. (2017). Perinatal Confidential Enquiry: term, singleton, intrapartum stillbirth and intrapartum-related neonatal death (9780993505973) <https://www.npeu.ox.ac.uk/downloads/files/mbrrace-uk/reports/MBRRACE-UK%20Intrapartum%20Confidential%20Enquiry%20Report%202017%20-%20final%20version.pdf> , <https://www.npeu.ox.ac.uk/mbrrace-uk/reports> , <https://www.npeu.ox.ac.uk/mbrrace-uk>

Facchini, G. (2022). Low staffing in the maternity ward: Keep calm and call the surgeon. Journal of Economic Behavior & Organization, 197, 370-394.

Freeman, M., Savva, N., & Scholtes, S. (2017). Gatekeepers at work: An empirical analysis of a maternity unit. Management Science, 63(10), 3147-3167.

Hollowell, J., Rowe, R., Townsend, J., Knight, M., Li, Y., Linsell, L., Redshaw, M., Brocklehurst, P., Macfarlane, A., & Marlow, N. (2015). The Birthplace in England national prospective cohort study: further analyses to enhance policy and service delivery decision-making for planned place of birth.

Isidore, J., & Rousseau, A. (2018). Administration of oxytocin during spontaneous labour: A national vignette-based study among midwives. Midwifery, 62, 214-219.

Kpéa, L., Bonnet, M.-P., Le Ray, C., Prunet, C., Ducloy-Bouthors, A.-S., & Blondel, B. (2015). Initial preference for labor without neuraxial analgesia and actual use: results from a national survey in France. Anesthesia & Analgesia, 121(3), 759-766.

Knape N, Mayer H, Schnepp W, zu Sayn-Wittgenstein F (2014). The association between attendance of midwives and workload of midwives with the mode of birth: secondary analyses in the German healthcare system. BMC pregnancy and childbirth, 14:1-13.

Lyndon, A., Simpson, K.R., Spetz, J., Zhong, J., Gay, C.L., Fletcher, J., Landstrom, G.L (2022). Nurse-reported staffing guidelines and exclusive breast milk feeding. Nursing research;71(6):432-40.

Mercer, B.M (2016). Delivery Volume, Nurse staffing and adverse perinatal outcomes [10D]. Obstetrics & Gynecology;127:36S-7S.

Robertson, K., Hardingham, I., D'Arcy, R., Reddy, A., Clacey, J. (2021). Delay in the induction of labour process: a retrospective cohort study and computer simulation of maternity unit workload. BMJ open, 11(9):e045577

Turner L, Culliford D, Ball J, Kitson-Reynolds E, Griffiths P (2022). The association between midwifery staffing levels and the experiences of mothers on postnatal wards: Cross sectional analysis of routine data. Women & Birth: Journal of the Australian College of Midwives, 35(6):e583-e9

Vanderlaan J. (2023) Midwifery workforce density moderates the association between independent practice and pregnancy outcomes. Journal of Midwifery & Women's Health, 68(5):588-95

Wilson, B.L., Butler R.J., (2021). Identifying optimal labor and delivery nurse staffing: The case of cesarean births and nursing hours. Nursing outlook, 69(1):84-95.

Zbiri, S., Rozenberg, P., Goffinet, F., & Milcent, C. (2018). Cesarean delivery rate and staffing levels of the maternity unit. PloS one, 13(11), e0207379

## Original Review

Cerbinskaite, A., Malone, S., McDermott, J., Loughney, A.D. (2011). Emergency caesarean section: influences on the decision‐to‐delivery interval. Journal of pregnancy; 2011(1):640379.

Gerova, V., Griffiths, P., Jones, S., Bick, D (2010). The association between midwifery staffing and outcomes in maternity units in England: observational study using routinely collected data. University of Southampton Institutional Repository; <https://eprints.soton.ac.uk/168511/>

Joyce, R., Webb, R., Peacock, J (2002). Predictors of obstetric intervention rates: case-mix, staffing levels and organisational factors of hospital of birth. Journal of Obstetrics and Gynaecology; 22(6):618-25.

Joyce, R., Webb, R., Peacock, J. (2004). Associations between perinatal interventions and hospital stillbirth rates and neonatal mortality. Archives of Disease in Childhood-Fetal and Neonatal Edition; 89(1):F51-F6.

Rowe, R.E., Townend, J., Brocklehurst, P., Knight, M., Macfarlane, A., McCourt, C., (2014). Service configuration, unit characteristics and variation in intervention rates in a national sample of obstetric units in England: an exploratory analysis. BMJ open, 2014;4(5):e005551.

Sandall, J., Murrells, T., Dodwell, M., Gibson, R., Bewley, S., Coxon, K. (2014). The efficient use of the maternity workforce and the implications for safety and quality in maternity care: a population-based, cross-sectional study. Health Services and Delivery Research. 2014; 2(38).

North Staffordshire Changing Childbirth Research Team (NSCCRT) (2000). A randomised study of midwifery caseload care and traditional ‘shared-care'. Midwifery. 2000; 16(4):295-302.

Tucker, J., Parry, G., Penney, G., Page, M., Hundley, V. (2003). Is midwife workload associated with quality of process of care (continuous electronic fetal monitoring [CEFM]) and neonatal outcome indicators? A prospective study in consultant‐led labour wards in Scotland. Paediatric and perinatal epidemiology. 2003; 17(4):369-77.
