# Supplementary material for: The impact of midwife workload on delivery of care, and mother and baby outcomes in maternity settings in OECD countries: A systematic review
Source: PLoS One. 2025 Aug 25;20(8):e0329117. doi: 10.1371/journal.pone.0329117 (PMC12377604; doi:10.1371/journal.pone.0329117)
Supplement: S2 File — (DOCX) [file pone.0329117.s002.docx]

# Supporting Information S2. Search Strategies

## S2A: Search Strategies (main search)

### Academic Databases

**Cochrane Database of Systematic Reviews Issue 2 of 12, February 2022 and Cochrane Central Register of Controlled Trials Issue 2 of 12, February 2022 search simultaneously.**

Date searched: 21/02/2022; Records identified: 267

| **ID** | **Search** | **Hits** |
| --- | --- | --- |
| #1 | MeSH descriptor: [Midwifery] this term only | 355 |
| #2 | MeSH descriptor: [Nurse Midwives] this term only | 102 |
| #3 | (midwif* or midwiv*):ti,ab | 2299 |
| #4 | (maternity near/4 worker*):ti,ab | 7 |
| #5 | (maternity near/4 staff*):ti,ab | 35 |
| #6 | (maternity near/4 assistant*):ti,ab | 7 |
| #7 | (midwi* near/4 assistant*):ti,ab | 16 |
| #8 | (midwi* near/4 staff*):ti,ab | 54 |
| #9 | (midwi* near/4 worker*):ti,ab | 44 |
| #10 | (msw* not ("municipal solid" or "municipal solid" or "male sex work*" or "medical social work*")):ti,ab | 254 |
| #11 | #1 or #2 or #3 or #4 or #5 or #6 or #7 or #8 or #9 or #10 | 2699 |
| #12 | (staff* near/4 (level* or ratio* or resourc* or model* or number* or mix* or rota* or rosta* or roster* or schedul* or overtime or supervision or supervisory or sufficient* or sufficiency or adequate* or adequac* or target* or insufficient* or insufficienc* or inadequate* or inadequac* or short or  shortage* or efficient* or efficienc* or inefficien* or burnout or stress or fatigue or magnet)):ti,ab | 1847 |
| #13 | (skill?mix* or "skill? mix*"):ti,ab | 34 |
| #14 | (staffmix* or "staff mix*"):ti,ab | 5 |
| #15 | staffing:ti,ab | 556 |
| #16 | understaff*:ti,ab | 27 |
| #17 | "under staff*":ti,ab | 10 |
| #18 | MeSH descriptor: [Personnel Staffing and Scheduling] explode all trees | 656 |
| #19 | MeSH descriptor: [Health Workforce] this term only | 23 |
| #20 | manpower:ti,ab | 154 |
| #21 | (workload* or workforce* or shift or shiftwork* or shifts or overtime or capacity):ti,ab | 50895 |
| #22 | MeSH descriptor: [Workload] this term only | 419 |
| #23 | ("missed care" or "missing care"):ti,ab | 3 |
| #24 | "care left undone":ti,ab | 1 |
| #25 | (hours near/3 day):ti,ab | 4997 |
| #26 | (work* near/3 hours):ti,ab | 1104 |
| #27 | (hours near/3 care):ti,ab | 491 |
| #28 | (caseload or "case load*"):ti,ab | 285 |
| #29 | (turnover or "turn over"):ti,ab | 5766 |
| #30 | (FTE or "full-time equivalent"):ti,ab | 67 |
| #31 | MeSH descriptor: [Occupational Stress] explode all trees | 451 |
| #32 | #12 or #13 or #14 or #15 or #16 or #17 or #18 or #19 or #20 or #21 or #22 or #23 or #24 or #25 or #26 or #27 or #28 or #29 or #30 or #31 | 65367 |
| #33 | #11 and #32 | 210 |
| #34 | (midwi* near/4 assistant* near/4 (level* or ratio* or resourc* or model* or number* or mix* or rota* or rosta* or roster* or schedul* or overtime or supervision or supervisory or sufficient* or sufficiency or adequate* or adequac* or target* or insufficient* or insufficienc* or inadequate* or inadequac* or short or shortage* or efficient* or efficienc* or inefficien* or burnout or stress or fatigue or magnet)):ti,ab | 0 |
| #35 | (midwi* near/4 worker* near/4 (level* or ratio* or resourc* or model* or number* or mix* or rota* or rosta* or roster* or schedul* or overtime or supervision or supervisory or sufficient* or sufficiency or adequate* or adequac* or target* or insufficient* or insufficienc* or inadequate* or inadequac* or short or shortage* or efficient* or efficienc* or inefficien* or burnout or stress or fatigue or magnet)):ti,ab | 0 |
| #36 | (maternity near/4 assistant* near/4 (level* or ratio* or resourc* or model* or number* or mix* or rota* or rosta* or roster* or schedul* or overtime or supervision or supervisory or sufficient* or sufficiency or adequate* or adequac* or target* or insufficient* or insufficienc* or inadequate* or inadequac* or short or shortage* or efficient* or efficienc* or inefficien* or burnout or stress or fatigue or magnet)):ti,ab | 1 |
| #37 | (maternity near/4 worker* near/4 (level* or ratio* or resourc* or model* or number* or mix* or rota* or rosta* or roster* or schedul* or overtime or supervision or supervisory or sufficient* or sufficiency or adequate* or adequac* or target* or insufficient* or insufficienc* or inadequate* or inadequac* or short or shortage* or efficient* or efficienc* or inefficien* or burnout or stress or fatigue or magnet)):ti,ab | 1 |
| #38 | (midwi* near/4 (level* or ratio* or resourc* or model* or number* or mix* or rota* or rosta* or roster* or schedul* or overtime or supervision or supervisory or sufficient* or sufficiency or adequate* or adequac* or target* or insufficient* or insufficienc* or inadequate* or inadequac* or short or |  |
| #39 | "named midwi*":ti,ab | 0 |
| #40 | #33 or #34 or #35 or #36 or #37 or #38 or #39 | 342 |
| #41 | MeSH descriptor: [Perinatal Care] explode all trees and with qualifier(s): [standards - ST, economics - EC, organization & administration - OG] | 136 |
| #42 | MeSH descriptor: [Delivery Rooms] this term only and with qualifier(s): [standards - ST, economics - EC, organization & administration - OG] | 15 |
| #43 | MeSH descriptor: [Birthing Centers] this term only and with qualifier(s): [standards - ST, economics - EC, organization & administration - OG] | 7 |
| #44 | MeSH descriptor: [Midwifery] this term only and with qualifier(s): [economics - EC, organization & administration - OG] | 34 |
| #45 | MeSH descriptor: [Nurse Midwives] this term only and with qualifier(s): [economics - EC, organization & administration - OG] | 15 |
| #46 | #40 or #41 or #42 or #43 or #44 or #45 | 511 |
| #47 | #40 or #41 or #42 or #43 or #44 or #45 with Publication Year from 2014 to 2022, with Cochrane Library publication date Between Jan 2014 and Dec 2022, in Trials | 267 |

**CINAHL (EBSCOhost)**

Date searched: 21/02/2022; Records identified: 3946

| **ID** | **Search** | **Hits** |
| --- | --- | --- |
| S43 | S42 Limiters - English Language | 3,496 |
| S42 | S30 NOT S40- Limiters - Published Date: 20140101-20221231 |  |
|  | 3,545 |  |
| S41 | S30 NOT S40 | 6,844 |
| S40 | S36 NOT S39 | 366,558 |
| S39 | S37 OR S38 | 1,685,154 |
| S38 | (MH "Australia+") OR (MH "Europe") OR (MH "Austria") OR (MH "Baltic States+") OR (MH "Belgium") OR (MH "Canada+") OR (MH "Chile") OR (MH "Colombia") OR (MH "Costa Rica") OR (MH "Czech Republic") OR (MH "Scandinavia+") OR (MH "France") OR (MH "Germany+") OR (MH "Greece") OR (MH "Hungary") OR (MH "Iceland") OR (MH "Ireland") OR (MH "Italy") OR (MH "Israel") OR (MH "Japan") OR (MH "South Korea") OR (MH "Luxembourg") OR (MH "Mexico") OR (MH "Netherlands") OR (MH "New Zealand") OR (MH "North America") OR (MH "Poland") OR (MH "Portugal") OR (MH "Slovakia") OR (MH "Slovenia") OR (MH "Spain") OR (MH "Switzerland") OR (MH "Turkey") OR (MH "United Kingdom+") OR (MH "United States+") | 1,678,591 |
| S37 | (MH "Developed Countries") or (MH "European Union") or (MH "Organisation for Economic Co-Operation and Development") | 10,251 |
| S36 | (S31 OR S32 OR S33 OR S34 OR S35) 404,734 |  |
| S35 | (MH "Africa+") | 92,324 |
| S34 | (MH "Asia, Southeastern+") OR (MH "China+") OR (MH "Hong Kong") OR (MH "Macao") OR (MH "Mongolia") OR (MH "North Korea") OR (MH "Taiwan") OR (MH "Atlantic Islands") OR (MH "Indian Ocean Islands+") OR (MH "Melanesia+") OR (MH "Micronesia+") OR (MH "Polynesia+") | 127,733 |
| S33 | (MH "Bangladesh") OR (MH "Bhutan") OR (MH "India") OR (MH "Yemen") OR (MH "United Arab Emirates") OR (MH "Syria") OR (MH "Saudi Arabia") OR (MH "Qatar") OR (MH "Oman") OR (MH "Lebanon") OR (MH "Kuwait") OR (MH "Jordan") OR (MH "Iraq") OR (MH "Iran") OR (MH "Bahrain") OR (MH "Afghanistan") OR (MH "Nepal") OR (MH "Pakistan") OR (MH "Sri Lanka") OR (MH "Asia, Central+") | 108,974 |
| S32 | (MH "Argentina") OR (MH "Bolivia") OR (MH "Brazil") OR (MH "Ecuador") OR (MH "French Guiana") OR (MH "Guyana") OR (MH "Paraguay") OR (MH "Peru") OR (MH "Suriname") OR (MH "Uruguay") OR (MH "Venezuela") OR (MH "Belize") OR (MH "El Salvador") OR (MH "Guatemala") OR (MH "Honduras") OR (MH "Nicaragua") OR (MH "Panama+") or (MH "West Indies+") | 72,972 |
| S31 | (MH "Albania") OR (MH "Andorra") OR (MH "Armenia") OR (MH "Azerbaijan") or (MH "Byelarus") OR (MH "Bosnia-Herzegovina") OR (MH "Croatia") OR (MH "Bulgaria") OR (MH "Georgia (Republic)") OR (MH "Gibraltar") OR (MH "Liechtenstein") OR (MH "Macedonia (Republic)") OR (MH "Moldova") OR (MH "Monaco") OR (MH "Romania") OR (MH "Russia") OR (MH "San Marino") OR (MH "Serbia") OR (MH "Ukraine") OR (MH "Yugoslavia") | 11,810 |
| S30 | S19 OR S20 OR S21 OR S22 OR S23 OR S24 OR S25 OR S26 OR S27 OR S29 | 7,758 |
| S29 | S7 AND S28 | 5,287 |
| S28 | S8 OR S9 OR S10 OR S11 OR S12 OR S13 OR S14 OR S15 OR S16 OR S17 OR S18 | 290,943 |
| S27 | (MH "Perinatal Care/EC/ST/OG") | 394 |
| S26 | (MH "Delivery Rooms+/EC/OG/ST") | 104 |
| S25 | (MH "Midwives+/EC/ST") 222 |  |
| S24 | TI "named midwi*" or AB "named midwi*" | 19 |
| S23 | ( TI (midwi* N3 (level* OR ratio* OR resourc* OR model* OR number* OR mix* OR rota* OR rosta* OR roster* OR schedul* OR overtime OR supervision OR supervisory OR sufficient* OR sufficiency OR adequate* OR adequac* OR target* OR insufficient* OR insufficienc* OR inadequate* OR inadequac* OR short OR shortage* OR efficient* OR efficienc* OR inefficien* OR burnout OR stress OR fatigue OR magnet)) ) OR ( AB (midwi* N3 (level* OR ratio* OR resourc* OR model* OR number* OR mix* OR rota* OR rosta* OR roster* OR schedul* OR overtime OR supervision OR supervisory OR sufficient* OR sufficiency OR adequate* OR adequac* OR target* OR insufficient* OR insufficienc* OR inadequate* OR inadequac* OR short OR shortage* OR efficient* OR efficienc* OR inefficien* OR burnout OR stress OR fatigue OR magnet)) ) 2,579 |  |
| S22 | ( TI (maternity N3 worker* N3 (level* OR ratio* OR resourc* OR model* OR number* OR mix* OR rota* OR rosta* OR roster* OR schedul* OR overtime OR supervision OR supervisory OR sufficient* OR sufficiency OR adequate* OR adequac* OR target* OR insufficient* OR insufficienc* OR inadequate* OR inadequac* OR short OR shortage* OR efficient* OR efficienc* OR inefficien* OR burnout OR stress OR fatigue OR magnet)) ) OR ( AB (maternity N3 worker* N3 (level* OR ratio* OR resourc* OR model* OR number* OR mix* OR rota* OR rosta* OR roster* OR schedul* OR overtime OR supervision OR supervisory OR sufficient* OR sufficiency OR adequate* OR adequac* OR target* OR insufficient* OR insufficienc* OR inadequate* OR inadequac* OR short OR shortage* OR efficient* OR efficienc* OR inefficien* OR burnout OR stress OR fatigue OR magnet)) ) | 5 |
| S21 | ( TI (maternity N3 assistant* N3 (level* OR ratio* OR resourc* OR model* OR number* OR mix* OR rota* OR rosta* OR roster* OR schedul* OR overtime OR supervision OR supervisory OR sufficient* OR sufficiency OR adequate* OR adequac* OR target* OR insufficient* OR insufficienc* OR inadequate* OR inadequac* OR short OR shortage* OR efficient* OR efficienc* OR inefficien* OR burnout OR stress OR fatigue OR magnet)) ) OR ( AB (maternity N3 assistant* N3 (level* OR ratio* OR resourc* OR model* OR number* OR mix* OR rota* OR rosta* OR roster* OR schedul* OR overtime OR supervision OR supervisory OR sufficient* OR sufficiency OR adequate* OR adequac* OR target* OR insufficient* OR insufficienc* OR inadequate* OR inadequac* OR short OR shortage* OR efficient* OR efficienc* OR inefficien* OR burnout OR stress OR fatigue OR magnet)) ) | 4 |
| S20 | ( TI (midwi* N3 worker* N3 (level* OR ratio* OR resourc* OR model* OR number* OR mix* OR rota* OR rosta* OR roster* OR schedul* OR overtime OR supervision OR supervisory OR sufficient* OR sufficiency OR adequate* OR adequac* OR target* OR insufficient* OR insufficienc* OR inadequate* OR inadequac* OR short OR shortage* OR efficient* OR efficienc* OR inefficien* OR burnout OR stress OR fatigue OR magnet)) ) OR ( AB (midwi* N3 worker* N3 (level* OR ratio* OR resourc* OR model* OR number* OR mix* OR rota* OR rosta* OR roster* OR schedul* OR overtime OR supervision OR supervisory OR sufficient* OR sufficiency OR adequate* OR adequac* OR target* OR insufficient* OR insufficienc* OR inadequate* OR inadequac* OR short OR shortage* OR efficient* OR efficienc* OR inefficien* OR burnout OR stress OR fatigue OR magnet)) ) | 12 |
| S19 | ( TI (midwi* N3 assistant* N3 (level* OR ratio* OR resourc* OR model* OR number* OR mix* OR rota* OR rosta* OR roster* OR schedul* OR overtime OR supervision OR supervisory OR sufficient* OR sufficiency OR adequate* OR adequac* OR target* OR insufficient* OR insufficienc* OR inadequate* OR inadequac* OR short OR shortage* OR efficient* OR efficienc* OR inefficien* OR burnout OR stress OR fatigue OR magnet)) ) OR ( AB (midwi* N3 assistant* N3 (level* OR ratio* OR resourc* OR model* OR number* OR mix* OR rota* OR rosta* OR roster* OR schedul* OR overtime OR supervision OR supervisory OR sufficient* OR sufficiency OR adequate* OR adequac* OR target* OR insufficient* OR insufficienc* OR inadequate* OR inadequac* OR short OR shortage* OR efficient* OR efficienc* OR inefficien* OR burnout OR stress OR fatigue OR magnet)) ) | 7 |
| S18 | ( TI (skill#mix* OR "skill# mix*" OR staffmix* OR "staff mix*" OR staffing OR understaff* OR manpower OR workload* OR workforce* OR shift OR shiftwork* OR shifts OR overtime OR capacity OR "missed care" OR "missing care" OR "care left undone" OR (hours N2 day) OR (work* N2 hours) OR (hours N2 care) OR caseload OR "case load*" OR turnover OR "turn over" OR FTE OR "full-time equivalent") ) OR ( AB (skill#mix* OR "skill# mix*" OR staffmix* OR "staff mix*" OR staffing OR understaff* OR manpower OR workload* OR workforce* OR shift OR shiftwork* OR shifts OR overtime OR capacity OR "missed care" OR "missing care" OR "care left undone" OR (hours N2 day) OR (work* N2 hours) OR (hours N2 care) OR caseload OR "case load*" OR turnover OR "turn over" OR FTE OR "full-time equivalent") ) | 209,146 |
| S17 | ( TI (staff* N3 (level* OR ratio* OR resourc* OR model* OR number* OR mix* OR rota* OR rosta* OR roster* OR schedul* OR overtime OR supervision OR supervisory OR sufficient* OR sufficiency OR adequate* OR adequac* OR target* OR insufficient* OR insufficienc* OR inadequate* OR inadequac* OR short OR shortage* OR efficient* OR efficienc* OR inefficien* OR burnout OR stress OR fatigue OR magnet)) ) OR ( AB (staff* N3 (level* OR ratio* OR resourc* OR model* OR number* OR mix* OR rota* OR rosta* OR roster* OR schedul* OR overtime OR supervision OR supervisory OR sufficient* OR sufficiency OR adequate* OR adequac* OR target* OR insufficient* OR insufficienc* OR inadequate* OR inadequac* OR short OR shortage* OR efficient* OR efficienc* OR inefficien* OR burnout OR stress OR fatigue OR magnet)) ) 18,590 |  |
| S16 | (MH "Stress, Occupational") | 18,365 |
| S15 | (MH "Nursing Manpower") 6,028 |  |
| S14 | (MH "Workforce") | 12,802 |
| S13 | (MH "Magnet Hospitals") 2,699 |  |
| S12 | (MH "Personnel Turnover") 5,577 |  |
| S11 | (MH "Burnout, Professional+") | 13,467 |
| S10 | (MH "Personnel Shortage+") 17,202 |  |
| S9 | (MH "Workload") | 17,246 |
| S8 | (MH "Personnel Staffing and Scheduling+") | 33,969 |
| S7 | S1 OR S2 OR S3 OR S4 OR S5 OR S6 45,027 |  |
| S6 | TI (msw* NOT ("municipal solid" or "male sex work*" or "medical social work*")) OR AB (msw* NOT ("municipal solid" or "male sex work*" or "medical social work*")) | 799 |
| S5 | TI (maternity N3 assistant*) or AB (maternity N3 assistant*) | 52 |
| S4 | TI (maternity N3 staff*) or AB (maternity N3 staff*) | 258 |
| S3 | TI (maternity N3 worker*) or AB (maternity N3 worker*) | 156 |
| S2 | TI (midwif* or midwiv*) or AB (midwif* or midwiv*) | 36,934 |
| S1 | (MH "Midwives+") | 16,451 |

**ECONLit (EBSCOhost)**

Date searched: 21/02/2022; Records identified: 23

| **ID** | **Search** | **Hits** |
| --- | --- | --- |
| S18 | S17 Limiters - Published Date: 20140101-20221231 | 23 |
| S17 | S9 OR S10 OR S11 OR S12 OR S13 OR S14 OR S16 | 35 |
| S16 | S6 AND S15 | 26 |
| S15 | S7 OR S8 | 74,952 |
| S14 | TI "named midwi*" or AB "named midwi*" | 0 |
| S13 | ( TI (midwi* N3 (level* OR ratio* OR resourc* OR model* OR number* OR mix* OR rota* OR rosta* OR roster* OR schedul* OR overtime OR supervision OR supervisory OR sufficient* OR sufficiency OR adequate* OR adequac* OR target* OR insufficient* OR insufficienc* OR inadequate* OR inadequac* OR short OR shortage* OR efficient* OR efficienc* OR inefficien* OR burnout OR stress OR fatigue OR magnet)) ) OR ( AB (midwi* N3 (level* OR ratio* OR resourc* OR model* OR number* OR mix* OR rota* OR rosta* OR roster* OR schedul* OR overtime OR supervision OR supervisory OR sufficient* OR sufficiency OR adequate* OR adequac* OR target* OR insufficient* OR insufficienc* OR inadequate* OR inadequac* OR short OR shortage* OR efficient* OR efficienc* OR inefficien* OR burnout OR stress OR fatigue OR magnet)) ) | 15 |
| S12 | ( TI (maternity N3 worker* N3 (level* OR ratio* OR resourc* OR model* OR number* OR mix* OR rota* OR rosta* OR roster* OR schedul* OR overtime OR supervision OR supervisory OR sufficient* OR sufficiency OR adequate* OR adequac* OR target* OR insufficient* OR insufficienc* OR inadequate* OR inadequac* OR short OR shortage* OR efficient* OR efficienc* OR inefficien* OR burnout OR stress OR fatigue OR magnet)) ) OR ( AB (maternity N3 worker* N3 (level* OR ratio* OR resourc* OR model* OR number* OR mix* OR rota* OR rosta* OR roster* OR schedul* OR overtime OR supervision OR supervisory OR sufficient* OR sufficiency OR adequate* OR adequac* OR target* OR insufficient* OR insufficienc* OR inadequate* OR inadequac* OR short OR shortage* OR efficient* OR efficienc* OR inefficien* OR burnout OR stress OR fatigue OR magnet)) ) | 0 |
| S11 | ( TI (maternity N3 assistant* N3 (level* OR ratio* OR resourc* OR model* OR number* OR mix* OR rota* OR rosta* OR roster* OR schedul* OR overtime OR supervision OR supervisory OR sufficient* OR sufficiency OR adequate* OR adequac* OR target* OR insufficient* OR insufficienc* OR inadequate* OR inadequac* OR short OR shortage* OR efficient* OR efficienc* OR inefficien* OR burnout OR stress OR fatigue OR magnet)) ) OR ( AB (maternity N3 assistant* N3 (level* OR ratio* OR resourc* OR model* OR number* OR mix* OR rota* OR rosta* OR roster* OR schedul* OR overtime OR supervision OR supervisory OR sufficient* OR sufficiency OR adequate* OR adequac* OR target* OR insufficient* OR insufficienc* OR inadequate* OR inadequac* OR short OR shortage* OR efficient* OR efficienc* OR inefficien* OR burnout OR stress OR fatigue OR magnet)) ) | 0 |
| S10 | ( TI (midwi* N3 worker* N3 (level* OR ratio* OR resourc* OR model* OR number* OR mix* OR rota* OR rosta* OR roster* OR schedul* OR overtime OR supervision OR supervisory OR sufficient* OR sufficiency OR adequate* OR adequac* OR target* OR insufficient* OR insufficienc* OR inadequate* OR inadequac* OR short OR shortage* OR efficient* OR efficienc* OR inefficien* OR burnout OR stress OR fatigue OR magnet)) ) OR ( AB (midwi* N3 worker* N3 (level* OR ratio* OR resourc* OR model* OR number* OR mix* OR rota* OR rosta* OR roster* OR schedul* OR overtime OR supervision OR supervisory OR sufficient* OR sufficiency OR adequate* OR adequac* OR target* OR insufficient* OR insufficienc* OR inadequate* OR inadequac* OR short OR shortage* OR efficient* OR efficienc* OR inefficien* OR burnout OR stress OR fatigue OR magnet)) ) | 0 |
| S9 | ( TI (midwi* N3 assistant* N3 (level* OR ratio* OR resourc* OR model* OR number* OR mix* OR rota* OR rosta* OR roster* OR schedul* OR overtime OR supervision OR supervisory OR sufficient* OR sufficiency OR adequate* OR adequac* OR target* OR insufficient* OR insufficienc* OR inadequate* OR inadequac* OR short OR shortage* OR efficient* OR efficienc* OR inefficien* OR burnout OR stress OR fatigue OR magnet)) ) OR ( AB (midwi* N3 assistant* N3 (level* OR ratio* OR resourc* OR model* OR number* OR mix* OR rota* OR rosta* OR roster* OR schedul* OR overtime OR supervision OR supervisory OR sufficient* OR sufficiency OR adequate* OR adequac* OR target* OR insufficient* OR insufficienc* OR inadequate* OR inadequac* OR short OR shortage* OR efficient* OR efficienc* OR inefficien* OR burnout OR stress OR fatigue OR magnet)) ) | 0 |
| S8 | ( TI (skill#mix* OR "skill# mix*" OR staffmix* OR "staff mix*" OR staffing OR understaff* OR manpower OR workload* OR workforce* OR shift OR shiftwork* OR shifts OR overtime OR capacity OR "missed care" OR "missing care" OR "care left undone" OR (hours N2 day) OR (work* N2 hours) OR (hours N2 care) OR caseload OR "case load*" OR turnover OR "turn over" OR FTE OR "full-time equivalent") ) OR ( AB (skill#mix* OR "skill# mix*" OR staffmix* OR "staff mix*" OR staffing OR understaff* OR manpower OR workload* OR workforce* OR shift OR shiftwork* OR shifts OR overtime OR capacity OR "missed care" OR "missing care" OR "care left undone" OR (hours N2 day) OR (work* N2 hours) OR (hours N2 care) OR caseload OR "case load*" OR turnover OR "turn over" OR FTE OR "full-time equivalent") ) | 74,496 |
| S7 | ( TI (staff* N3 (level* OR ratio* OR resourc* OR model* OR number* OR mix* OR rota* OR rosta* OR roster* OR schedul* OR overtime OR supervision OR supervisory OR sufficient* OR sufficiency OR adequate* OR adequac* OR target* OR insufficient* OR insufficienc* OR inadequate* OR inadequac* OR short OR shortage* OR efficient* OR efficienc* OR inefficien* OR burnout OR stress OR fatigue OR magnet)) ) OR ( AB (staff* N3 (level* OR ratio* OR resourc* OR model* OR number* OR mix* OR rota* OR rosta* OR roster* OR schedul* OR overtime OR supervision OR supervisory OR sufficient* OR sufficiency OR adequate* OR adequac* OR target* OR insufficient* OR insufficienc* OR inadequate* OR inadequac* OR short OR shortage* OR efficient* OR efficienc* OR inefficien* OR burnout OR stress OR fatigue OR magnet)) ) 828 |  |
| S6 | S1 OR S2 OR S3 OR S4 OR S5 | 145 |
| S5 | TI (msw* NOT "municipal solid") OR AB (msw* NOT "municipal solid") | 16 |
| S4 | TI (maternity N3 assistant*) or AB (maternity N3 assistant*) | 20 |
| S3 | TI (maternity N3 staff*) or AB (maternity N3 staff*) | 3 |
| S2 | TI (maternity N3 worker*) or AB (maternity N3 worker*) | 8 |
| S1 | TI (midwif* or midwiv*) or AB (midwif* or midwiv*) | 118 |

**Embase Classic + Embase (Ovid) 1947 to 2022 February 18**

Date searched: 21/02/2022; Records identified: 691

| **ID** | **Search** | **Hits** |
| --- | --- | --- |
| 1 | exp midwife/ | 35111 |
| 2 | (midwif* or midwiv*).tw. | 30937 |
| 3 | Nurse Midwife/ | 7040 |
| 4 | (maternity adj3 worker*).tw. | 118 |
| 5 | (maternity adj3 staff*).tw. | 425 |
| 6 | (maternity adj3 assistant*).tw. | 42 |
| 7 | (msw* not ("municipal solid" or "male sex work*" or "medical social work*")).tw. | 2494 |
| 8 | or/1-7 [Maternity staff] | 47126 |
| 9 | (staff* adj3 (level* or ratio* or resourc* or model* or number* or mix* or rota* or rosta* or roster* or schedul* or overtime or supervision or supervisory or sufficient* or sufficiency or adequate* or adequac* or target* or insufficient* or insufficienc* or inadequate* or inadequac* or short or shortage* or efficient* or efficienc* or inefficien* or burnout or stress or fatigue or magnet)).tw. | 27455 |
| 10 | (skill?mix* or "skill? mix*").tw. | 1411 |
| 11 | (staffmix* or "staff mix*").tw. | 116 |
| 12 | staffing.tw. | 21742 |
| 13 | understaff*.tw. | 910 |
| 14 | "under staff*".tw. | 98 |
| 15 | skill mix/ | 421 |
| 16 | personnel management/ | 59687 |
| 17 | exp health care personnel management/ | 3500 |
| 18 | exp workforce/ | 9647 |
| 19 | manpower planning/ | 924 |
| 20 | work schedule/ | 9596 |
| 21 | workload/ | 50176 |
| 22 | working time/ | 10601 |
| 23 | exp shift worker/ | 6396 |
| 24 | manpower.tw. | 10559 |
| 25 | (workload* or workforce* or shift or shiftwork* or shifts or overtime or capacity).tw. | 1206060 |
| 26 | magnet hospital/ | 48 |
| 27 | burnout/ or professional burnout/ | 23638 |
| 28 | exp personnel shortage/ | 2995 |
| 29 | ("missed care" or "missing care").tw. | 266 |
| 30 | "care left undone".tw. | 45 |
| 31 | (hours adj2 day).tw. | 14696 |
| 32 | (work* adj2 hours).tw. | 16884 |
| 33 | (hours adj2 care).tw. | 2029 |
| 34 | (caseload or "case load*").tw. | 6518 |
| 35 | (turnover or "turn over").tw. | 130097 |
| 36 | (FTE or "full-time equivalent").tw. | 2465 |
| 37 | exp job stress/ | 13100 |
| 38 | or/9-37 | 1511398 |
| 39 | 8 and 38 | 4527 |
| 40 | (midwi* adj3 assistant* adj3 (level* or ratio* or resourc* or model* or number* or mix* or rota* or rosta* or roster* or schedul* or overtime or supervision or supervisory or sufficient* or sufficiency or adequate* or adequac* or target* or insufficient* or insufficienc* or inadequate* or inadequac* or short or shortage* or efficient* or efficienc* or inefficien* or burnout or stress or fatigue or magnet)).tw. | 7 |
| 41 | (midwi* adj3 worker* adj3 (level* or ratio* or resourc* or model* or number* or mix* or rota* or rosta* or roster* or schedul* or overtime or supervision or supervisory or sufficient* or sufficiency or adequate* or adequac* or target* or insufficient* or insufficienc* or inadequate* or inadequac* or short or shortage* or efficient* or efficienc* or inefficien* or burnout or stress or fatigue or magnet)).tw. | 4 |
| 42 | (maternity adj3 assistant* adj3 (level* or ratio* or resourc* or model* or number* or mix* or rota* or rosta* or roster* or schedul* or overtime or supervision or supervisory or sufficient* or sufficiency or adequate* or adequac* or target* or insufficient* or insufficienc* or inadequate* or inadequac* or short or shortage* or efficient* or efficienc* or inefficien* or burnout or stress or fatigue or magnet)).tw. | 0 |
| 43 | (maternity adj3 worker* adj3 (level* or ratio* or resourc* or model* or number* or mix* or rota* or rosta* or roster* or schedul* or overtime or supervision or supervisory or sufficient* or sufficiency or adequate* or adequac* or target* or insufficient* or insufficienc* or inadequate* or inadequac* or short or shortage* or efficient* or efficienc* or inefficien* or burnout or stress or fatigue or magnet)).tw. | 5 |
| 44 | (midwi* adj3 (level* or ratio* or resourc* or model* or number* or mix* or rota* or rosta* or roster* or schedul* or overtime or supervision or supervisory or sufficient* or sufficiency or adequate* or adequac* or target* or insufficient* or insufficienc* or inadequate* or inadequac* or short or shortage* or efficient* or efficienc* or inefficien* or burnout or stress or fatigue or magnet)).tw. | 1689 |
| 45 | or/40-44 | 1699 |
| 46 | "named midwi*".tw. | 16 |
| 47 | 39 or 45 or 46 [Maternity Staff issues] | 5742 |
| 48 | nonhuman/ not exp human/ | 4933158 |
| 49 | 47 not 48 [Animal studies removed] | 5720 |
| 50 | afghanistan/ or africa/ or "africa south of the sahara"/ or albania/ or algeria/ or andorra/ or angola/ or argentina/ or "antigua and barbuda"/ or armenia/ or exp azerbaijan/ or bahamas/ or bahrain/ or bangladesh/ or barbados/ or belarus/ or belize/ or benin/ or bhutan/ or bolivia/ or borneo/ or exp "bosnia and herzegovina"/ or botswana/ or exp brazil/ or brunei darussalam/ or bulgaria/ or burkina faso/ or burundi/ or cambodia/ or cameroon/ or cape verde/ or central africa/ or central african republic/ or chad/ or exp china/ or comoros/ or congo/ or cook islands/ or coted'ivoire/ or croatia/ or cuba/ or cyprus/ or democratic republic congo/ or djibouti/ or dominica/ or dominican republic/ or ecuador/ or el salvador/ or egypt/ or equatorial guinea/ or eritrea/ or eswatini/ or ethiopia/ or exp "federated states of micronesia"/ or fiji/ or gabon/ or gambia/ or exp "georgia (republic)"/ or ghana/ or grenada/ or guatemala/ or guinea/ or guinea-bissau/ or guyana/ or haiti/ or honduras/ or exp india/ or exp indonesia/ or iran/ or exp iraq/ or jamaica/ or jordan/ or kazakhstan/ or kenya/ or kiribati/ or kosovo/ or kuwait/ or kyrgyzstan/ or laos/ or lebanon/ or liechtenstein/ or lesotho/ or liberia/ or libyan arab jamahiriya/ or madagascar/ or malawi/ or exp malaysia/ or maldives/ or mali/ or malta/ or mauritania/ or mauritius/ or melanesia/ or moldova/ or monaco/ or mongolia/ or "montenegro (republic)"/ or morocco/ or mozambique/ or myanmar/ or namibia/ or nauru/ or nepal/ or nicaragua/ or niger/ or nigeria/ or niue/ or north africa/ or oman/ or exp pakistan/ or palau/ or palestine/ or panama/ or papua new guinea/ or paraguay/ or peru/ or philippines/ or polynesia/ or qatar/ or "republic of north macedonia"/ or romania/ or exp russian federation/ or rwanda/ or sahel/ or "saint kitts and nevis"/ or "saint lucia"/ or "saint vincent and the grenadines"/ or saudi arabia/ or senegal/ or exp serbia/ or seychelles/ or sierra leone/ or singapore/ or "sao tome and principe"/ or solomon islands/ or exp somalia/ or south africa/ or south asia/ or south sudan/ or exp southeast asia/ or sri lanka/ or sudan/ or suriname/ or syrian arab republic/ or taiwan/ or tajikistan/ or tanzania/ or thailand/ or timor-leste/ or togo/ or tonga/ or "trinidad andtobago"/ or tunisia/ or turkmenistan/ or tuvalu/ or uganda/ or exp ukraine/ or exp united arab emirates/ or uruguay/ or exp uzbekistan/ or vanuatu/ or venezuela/ or viet nam/ or western sahara/ or yemen/ or zambia/ or zimbabwe/ | 1586019 |
| 51 | "organisation for economic co-operation and development"/ | 1920 |
| 52 | exp australia/ or "australia and new zealand"/ or austria/ or baltic states/ or exp belgium/ or exp canada/ or chile/ or colombia/ or costa rica/ or czech republic/ or denmark/ or estonia/ or europe/ or exp finland/ or exp france/ or exp germany/ or greece/ or hungary/ or iceland/ or ireland/ or israel/ or exp italy/ or japan/ or korea/ or latvia/ or lithuania/ or luxembourg/ or exp mexico/ or netherlands/ or new zealand/ or north america/ or exp norway/ or poland/ or exp portugal/ or scandinavia/ or sweden/ or slovakia/ or slovenia/ or south korea/ or exp spain/ or switzerland/ or exp united kingdom/ or "turkey (republic)"/ or exp united states/ or western europe/ | 3798628 |
| 53 | european union/ | 29388 |
| 54 | developed country/ | 35010 |
| 55 | or/51-54 | 3829836 |
| 56 | 50 not 55 [NICE Filter 2021 Non-OECD countries] | 1438360 |
| 57 | 49 not 56 [non-OECD countries removed] | 4592 |
| 58 | limit 57 to (editorial or letter or note) | 266 |
| 59 | 57 not 58 [ephemera removed] | 4326 |
| 60 | limit 59 to english language | 4136 |
| 61 | limit 60 to yr="2014 -Current" | 2149 |
| 62 | limit 61 to embase | 691 |

**HMIC Health Management Information Consortium (Ovid) 1979 to November 2021**

Date searched: 21/02/2022; Records identified: 110

| **ID** | **Search** | **Hits** |
| --- | --- | --- |
| 1 | exp midwives/ | 2302 |
| 2 | (midwif* or midwiv*).tw. | 4570 |
| 3 | midwifery/ | 666 |
| 4 | midwifery services/ | 542 |
| 5 | (maternity adj3 worker*).tw. | 22 |
| 6 | (maternity adj3 staff*).tw. | 52 |
| 7 | (maternity adj3 assistant*).tw. | 5 |
| 8 | (msw* not ("municipal solid" or "male sex work*" or "medical social work*")).tw. | 19 |
| 9 | maternity support workers/ | 5 |
| 10 | or/1-9 [Maternity Staff] | 5286 |
| 11 | exp staffing levels/ | 486 |
| 12 | skill mix/ | 615 |
| 13 | staff allocation/ | 67 |
| 14 | exp workload/ | 1433 |
| 15 | workload management/ or workload measurement/ | 70 |
| 16 | workload analysis/ | 24 |
| 17 | staff turnover/ | 270 |
| 18 | occupational stress/ | 1317 |
| 19 | (staff* adj3 (level* or ratio* or resourc* or model* or number* or mix* or rota* or rosta* or roster* or schedul* or overtime or supervision or supervisory or sufficient* or sufficiency or adequate* or adequac* or target* or insufficient* or insufficienc* or inadequate* or inadequac* or short or shortage* or efficient* or efficienc* or inefficien* or burnout or stress or fatigue or magnet)).tw. | 3714 |
| 20 | (skill?mix* or "skill? mix*").tw. | 684 |
| 21 | (staffmix* or "staff mix*").tw. | 21 |
| 22 | staffing.tw. | 3176 |
| 23 | understaff*.tw. | 82 |
| 24 | "under staff*".tw. | 12 |
| 25 | manpower.tw. | 1162 |
| 26 | workforce/ or workforce planning/ | 6305 |
| 27 | (workload* or workforce* or shift or shiftwork* or shifts or overtime or capacity).tw. | 15327 |
| 28 | ("missed care" or "missing care").tw. | 9 |
| 29 | "care left undone".tw. | 5 |
| 30 | (hours adj2 day).tw. | 180 |
| 31 | (work* adj2 hours).tw. | 1006 |
| 32 | (hours adj2 care).tw. | 418 |
| 33 | (caseload or "case load*").tw. | 475 |
| 34 | (turnover or "turn over").tw. | 741 |
| 35 | (FTE or "full-time equivalent").tw. | 86 |
| 36 | or/11-35 | 27893 |
| 37 | 10 and 36 | 686 |
| 38 | (midwi* adj3 assistant* adj3 (level* or ratio* or resourc* or model* or number* or mix* or rota* or rosta* or roster* or schedul* or overtime or supervision or supervisory or sufficient* or sufficiency or adequate* or adequac* or target* or insufficient* or insufficienc* or inadequate* or inadequac* or short or shortage* or efficient* or efficienc* or inefficien* or burnout or stress or fatigue or magnet)).tw. | 0 |
| 39 | (midwi* adj3 worker* adj3 (level* or ratio* or resourc* or model* or number* or mix* or rota* or rosta* or roster* or schedul* or overtime or supervision or supervisory or sufficient* or sufficiency or adequate* or adequac* or target* or insufficient* or insufficienc* or inadequate* or inadequac* or short or shortage* or efficient* or efficienc* or inefficien* or burnout or stress or fatigue or magnet)).tw. | 1 |
| 40 | (maternity adj3 assistant* adj3 (level* or ratio* or resourc* or model* or number* or mix* or rota* or rosta* or roster* or schedul* or overtime or supervision or supervisory or sufficient* or sufficiency or adequate* or adequac* or target* or insufficient* or insufficienc* or inadequate* or inadequac* or short or shortage* or efficient* or efficienc* or inefficien* or burnout or stress or fatigue or magnet)).tw. | 0 |
| 41 | (maternity adj3 worker* adj3 (level* or ratio* or resourc* or model* or number* or mix* or rota* or rosta* or roster* or schedul* or overtime or supervision or supervisory or sufficient* or sufficiency or adequate* or adequac* or target* or insufficient* or insufficienc* or inadequate* or inadequac* or short or shortage* or efficient* or efficienc* or inefficien* or burnout or stress or fatigue or magnet)).tw. | 1 |
| 42 | (midwi* adj3 (level* or ratio* or resourc* or model* or number* or mix* or rota* or rosta* or roster* or schedul* or overtime or supervision or supervisory or sufficient* or sufficiency or adequate* or adequac* or target* or insufficient* or insufficienc* or inadequate* or inadequac* or short or shortage* or efficient* or efficienc* or inefficien* or burnout or stress or fatigue or magnet)).tw. | 254 |
| 43 | or/38-42 | 255 |
| 44 | "named midwi*".tw. | 11 |
| 45 | 37 or 43 or 44 [Maternity Staffing issues] | 871 |
| 46 | limit 45 to yr="2014 -Current" | 110 |

**International HTA Database (INAHTA) https://database.inahta.org/**

Date searched: 21/02/2022; Records identified: 14

| **ID** | **Search** | **Hits** |
| --- | --- | --- |
| 19 | #18 year limited to 2014 – 2022 | 14 |
| 18 | #17 AND #5 | 38 |
| 17 | #16 OR #15 OR #14 OR #13 OR #12 OR #11 OR #10 OR #9 OR #8 OR #7 OR #6 | 7286 |
| 16 | "named midwi*" | 0 |
| 15 | turnover or "turn over" OR FTE or "full-time equivalent" or level* or ratio* or resourc* or model* or number* or rota* or rosta* or roster* or schedul* or overtime or supervision or supervisory or sufficient* or sufficiency or adequate* or adequac* or target* or insufficient* or insufficienc* or inadequate* or inadequac* or short or shortage* or efficient* or efficienc* or inefficien* or burnout or stress or fatigue or magnet | 7203 |
| 14 | "missed care" or "missing care" or "working hours" or "hours per day" or "hours of care" or caseload or "case load*" | 13 |
| 13 | manpower OR workload* OR workforce* OR shift OR shiftwork* OR shifts OR overtime OR capacity | 311 |
| 12 | skillmix* OR "skill* mix*" OR staffmix* OR "staff mix*" OR staffing OR understaff* 41 |  |
| 11 | "Work-Life Balance"[mhe] | 0 |
| 10 | "Occupational Stress"[mhe] | 9 |
| 9 | "Burnout, Professional"[mhe] | 8 |
| 8 | "Workload"[mhe] | 13 |
| 7 | "Health Workforce"[mhe] | 3 |
| 6 | "Personnel Staffing and Scheduling Information Systems"[mhe] 1 |  |
| 5 | #4 OR #3 OR #2 OR #1 |  |
| 4 | maternity AND (worker* OR staff* OR assistant*) | 7 |
| 3 | midwif* OR midwiv* | 44 |
| 2 | "Nurse Midwives"[mhe] | 2 |
| 1 | "Midwifery"[mhe] | 15 |

**Maternity & Infant Care Database (MIDIRS) (Ovid) 1971 to February 1, 2022**

Date searched: 21/02/2022; Records identified: 1243

| **ID** | **Search** | **Hits** |
| --- | --- | --- |
| 1 | midwi*.tw. | 37642 |
| 2 | (maternity adj3 worker*).tw. | 202 |
| 3 | (maternity adj3 staff*).tw. | 320 |
| 4 | (maternity adj3 assistant*).tw. | 86 |
| 5 | (msw* not ("municipal solid" or "male sex work*" or "medical social work*")).tw. | 57 |
| 6 | or/1-5 [Maternity staff] | 37862 |
| 7 | (staff* adj3 (level* or ratio* or resourc* or model* or number* or mix* or rota* or rosta* or roster* or schedul* or overtime or supervision or supervisory or sufficient* or sufficiency or adequate* or adequac* or target* or insufficient* or insufficienc* or inadequate* or inadequac* or short or shortage* or efficient* or efficienc* or inefficien* or burnout or stress or fatigue or magnet)).tw. | 2343 |
| 8 | (skill?mix* or "skill? mix*").tw. | 103 |
| 9 | (staffmix* or "staff mix*").tw. | 3 |
| 10 | staffing.tw. | 1470 |
| 11 | understaff*.tw. | 73 |
| 12 | "under staff*".tw. | 7 |
| 13 | manpower.tw. | 243 |
| 14 | (workload* or workforce* or shift or shiftwork* or shifts or overtime or capacity).tw. | 6332 |
| 15 | ("missed care" or "missing care").tw. | 11 |
| 16 | "care left undone".tw. | 1 |
| 17 | (hours adj2 day).tw. | 222 |
| 18 | (work* adj2 hours).tw. | 341 |
| 19 | (hours adj2 care).tw. | 33 |
| 20 | (caseload or "case load*").tw. | 441 |
| 21 | (turnover or "turn over").tw. | 583 |
| 22 | (FTE or "full-time equivalent").tw. | 42 |
| 23 | or/7-22 | 9898 |
| 24 | 6 and 23 | 3424 |
| 25 | (midwi* adj3 assistant* adj3 (level* or ratio* or resourc* or model* or number* or mix* or rota* or rosta* or roster* or schedul* or overtime or supervision or supervisory or sufficient* or sufficiency or adequate* or adequac* or target* or insufficient* or insufficienc* or inadequate* or inadequac* or short or shortage* or efficient* or efficienc* or inefficien* or burnout or stress or fatigue or magnet)).tw. | 0 |
| 26 | (midwi* adj3 worker* adj3 (level* or ratio* or resourc* or model* or number* or mix* or rota* or rosta* or roster* or schedul* or overtime or supervision or supervisory or sufficient* or sufficiency or adequate* or adequac* or target* or insufficient* or insufficienc* or inadequate* or inadequac* or short or shortage* or efficient* or efficienc* or inefficien* or burnout or stress or fatigue or magnet)).tw. | 2 |
| 27 | (maternity adj3 assistant* adj3 (level* or ratio* or resourc* or model* or number* or mix* or rota* or rosta* or roster* or schedul* or overtime or supervision or supervisory or sufficient* or sufficiency or adequate* or adequac* or target* or insufficient* or insufficienc* or inadequate* or inadequac* or short or shortage* or efficient* or efficienc* or inefficien* or burnout or stress or fatigue or magnet)).tw. | 2 |
| 28 | (maternity adj3 worker* adj3 (level* or ratio* or resourc* or model* or number* or mix* or rota* or rosta* or roster* or schedul* or overtime or supervision or supervisory or sufficient* or sufficiency or adequate* or adequac* or target* or insufficient* or insufficienc* or inadequate* or inadequac* or short or shortage* or efficient* or efficienc* or inefficien* or burnout or stress or fatigue or magnet)).tw. | 8 |
| 29 | (midwi* adj3 (level* or ratio* or resourc* or model* or number* or mix* or rota* or rosta* or roster* or schedul* or overtime or supervision or supervisory or sufficient* or sufficiency or adequate* or adequac* or target* or insufficient* or insufficienc* or inadequate* or inadequac* or short or shortage* or efficient* or efficienc* or inefficien* or burnout or stress or fatigue or magnet)).tw. | 2704 |
| 30 | or/25-29 | 2713 |
| 31 | "named midwi*".tw. | 79 |
| 32 | 24 or 30 or 31 | 5427 |
| 33 | limit 32 to (commentary or correspondence or editorial or news or news item or news release) | 1085 |
| 34 | 32 not 33 [Ephemera removed] | 4342 |
| 35 | limit 34 to yr="2014 -Current" | 1457 |
| 36 | (afghanistan* or africa* or albania* or algeria* or andorra* or angola* or argentin* or Antigua* or barbuda* or armenia* or azerbaijan* or Bahamas* or bahrain* or bangladesh* or barbados* or belarus* or belize* or benin* or bhutan* or bolivia* or borneo* or bosnia* or herzegovin* or botswan* or brazil* or brunei* or bulgaria* or burkina faso* or burundi* or cambodia* or cameroon* or cape verde* or chad* or china* or Chinese or comoros* or congo* or cook islands* or cote d'ivoire* or croatia* or cuba* or cyprus* or Cypriot* or djibouti* or dominica* or ecuador* or el salvador* or egypt* or eritrea* or eswatini* or ethiopia* or micronesia* or fiji* or gabon* or gambia* or georgia* or ghana* or grenada* or guatemala* or guinea* or guyana* or haiti* or honduras* or india* or indonesia* or iran* or iraq* or jamaica* or jordan* or kazakhstan* or kenya* or kiribati* or kosovo* or kuwait* or kyrgyzstan* or laos* or lebanon* or liechtenstein* or lesotho* or liberia* or libya* or madagascar* or malawi* or malaysia* or maldives* or mali* or malta* or mauritania* or mauritius* or melanesia* or moldova* or monaco* or mongolia* or montenegro * or morocc* or mozambique* or myanmar* or namibia* or nauru* or nepal* or nicaragua* or niger* or niue* or oman* or pakistan* or palau* or palestin* or panama* or paraguay* or peru* or philippin* or polynesia* or qatar* or macedonia* or romania* or russia* or rwanda* or sahel* or "saint kitts and nevis" or "saint lucia" or "saint vincent and the grenadines*" or saudi arabia* or senegal* or serbia* or seychelles* or sierra leone* or singapore* or "sao tome and principe*" or solomon islands* or somalia* or south* asia* or sri lanka* or sudan* or suriname* or syria* or taiwan* or tajikistan* or tanzania* or thail* or timor* or togo* or tonga* or "trinidad and tobago" or tunisia* or turkmenistan* or tuvalu* or uganda* or ukrain* or "united arab emirates" or uruguay* or uzbekistan* or vanuatu* or venezuela* or vietnam* or sahara* or yemen* or zambia* or zimbabwe*).ti. | 20686 |
| 37 | (australia* or austria* or baltic* or belgium* or canad* or chile* or colombia* or costa rica* or czech* or denmark* or danish or estonia* or europe* or finland* or france* or French or german* or greece* or hungar* or iceland* or ireland* or irish or israel* or italy* or Italian* or japan* or korea* or latvia* or lithuania* or luxembourg* or mexic* or netherlands* or new zealand* or north america* or norway* or norweigan* or polish or poland* or portug* or scandinavia* or swede* or Swedish or slovak* or slovenia* or spain* or switzerland* or swiss or English or welsh or Scottish or england or wales or scotland or Britain or British or united kingdom* or turkey* or Turkish or united states* or western europe* or european union).ti. | 18532 |
| 38 | 36 not 37 [Adapted NICE filter 2021] | 20293 |
| 39 | 35 not 38 | 1243 |

**Ovid MEDLINE(R) ALL <1946 to February 18, 2022>**

Date searched: 21/02/2022; Records identified: 2417

| **ID** | **Search** | **Hits** |
| --- | --- | --- |
| 1 | Midwifery/ | 20349 |
| 2 | (midwif* or midwiv*).tw. | 26516 |
| 3 | Nurse Midwives/ | 7401 |
| 4 | (maternity adj3 worker*).tw. | 99 |
| 5 | (maternity adj3 staff*).tw. | 280 |
| 6 | (maternity adj3 assistant*).tw. | 37 |
| 7 | (msw* not ("municipal solid" or "male sex work*" or "medical social work*")).tw. | 1586 |
| 8 | or/1-7 [Maternity staff] | 39622 |
| 9 | (staff* adj3 (level* or ratio* or resourc* or model* or number* or mix* or rota* or rosta* or roster* or schedul* or overtime or supervision or supervisory or sufficient* or sufficiency or adequate* or adequac* or target* or insufficient* or insufficienc* or inadequate* or inadequac* or short or shortage* or efficient* or efficienc* or inefficien* or burnout or stress or fatigue or magnet)).tw. | 19169 |
| 10 | (skill?mix* or "skill? mix*").tw. | 1186 |
| 11 | (staffmix* or "staff mix*").tw. | 109 |
| 12 | staffing.tw. | 15827 |
| 13 | understaff*.tw. | 705 |
| 14 | "under staff*".tw. | 79 |
| 15 | "personnel staffing and scheduling"/ or shift work schedule/ or work-life balance/ | 19517 |
| 16 | Health Workforce/ | 14052 |
| 17 | manpower.tw,fs. | 7523 |
| 18 | (workload* or workforce* or shift or shiftwork* or shifts or overtime or capacity).tw. | 968745 |
| 19 | Workload/ | 23119 |
| 20 | ("missed care" or "missing care").tw. | 259 |
| 21 | "care left undone".tw. | 47 |
| 22 | (hours adj2 day).tw. | 8501 |
| 23 | (work* adj2 hours).tw. | 11858 |
| 24 | (hours adj2 care).tw. | 1592 |
| 25 | (caseload or "case load*").tw. | 4549 |
| 26 | (turnover or "turn over").tw. | 104054 |
| 27 | (FTE or "full-time equivalent").tw. | 1511 |
| 28 | Burnout, Professional/ | 14790 |
| 29 | Occupational Stress/ | 2906 |
| 30 | Work Schedule Tolerance/ | 7283 |
| 31 | or/9-30 | 1163195 |
| 32 | 8 and 31 | 3176 |
| 33 | (midwi* adj3 assistant* adj3 (level* or ratio* or resourc* or model* or number* or mix* or rota* or rosta* or roster* or schedul* or overtime or supervision or supervisory or sufficient* or sufficiency or adequate* or adequac* or target* or insufficient* or insufficienc* or inadequate* or inadequac* or short or shortage* or efficient* or efficienc* or inefficien* or burnout or stress or fatigue or magnet)).tw. | 5 |
| 34 | (midwi* adj3 worker* adj3 (level* or ratio* or resourc* or model* or number* or mix* or rota* or rosta* or roster* or schedul* or overtime or supervision or supervisory or sufficient* or sufficiency or adequate* or adequac* or target* or insufficient* or insufficienc* or inadequate* or inadequac* or short or shortage* or efficient* or efficienc* or inefficien* or burnout or stress or fatigue or magnet)).tw. | 5 |
| 35 | (maternity adj3 assistant* adj3 (level* or ratio* or resourc* or model* or number* or mix* or rota* or rosta* or roster* or schedul* or overtime or supervision or supervisory or sufficient* or sufficiency or adequate* or adequac* or target* or insufficient* or insufficienc* or inadequate* or inadequac* or short or shortage* or efficient* or efficienc* or inefficien* or burnout or stress or fatigue or magnet)).tw. | 0 |
| 36 | (maternity adj3 worker* adj3 (level* or ratio* or resourc* or model* or number* or mix* or rota* or rosta* or roster* or schedul* or overtime or supervision or supervisory or sufficient* or sufficiency or adequate* or adequac* or target* or insufficient* or insufficienc* or inadequate* or inadequac* or short or shortage* or efficient* or efficienc* or inefficien* or burnout or stress or fatigue or magnet)).tw. | 4 |
| 37 | (midwi* adj3 (level* or ratio* or resourc* or model* or number* or mix* or rota* or rosta* or roster* or schedul* or overtime or supervision or supervisory or sufficient* or sufficiency or adequate* or adequac* or target* or insufficient* or insufficienc* or inadequate* or inadequac* or short or shortage* or efficient* or efficienc* or inefficien* or burnout or stress or fatigue or magnet)).tw. | 1439 |
| 38 | or/33-37 | 1445 |
| 39 | "named midwi*".tw. | 12 |
| 40 | 32 or 38 or 39 [Maternity staffing issues search 1] | 4243 |
| 41 | Perinatal Care/ec, og, st [Economics, Organization & Administration, Standards] | 1579 |
| 42 | Delivery Rooms/ec, og, st [Economics, Organization & Administration, Standards] | 510 |
| 43 | Birthing Centers/ec, og, st [Economics, Organization & Administration, Standards] | 388 |
| 44 | Midwifery/ec, og [Economics, Organization & Administration] | 2242 |
| 45 | Nurse Midwives/ec, og [Economics, Organization & Administration] | 862 |
| 46 | 41 or 42 or 43 or 44 or 45 [Maternity service staffing] | 5146 |
| 47 | 40 or 46 [All maternity staffing searches] | 8858 |
| 48 | exp animals/ not humans/ | 4961403 |
| 49 | 47 not 48 [Animal studies removed] | 8845 |
| 50 | afghanistan/ or africa/ or africa, northern/ or africa, central/ or africa, eastern/ or "africa south of the sahara"/ or africa, southern/ or africa, western/ or albania/ or algeria/ or andorra/ or angola/ or "antigua and barbuda"/ or argentina/ or armenia/ or azerbaijan/ or bahamas/ or bahrain/ or bangladesh/ or barbados/ or belize/ or benin/ or bhutan/ or bolivia/ or borneo/ or "bosnia and herzegovina"/ or botswana/ or brazil/ or brunei/ or bulgaria/ or burkina faso/ or burundi/ or cabo verde/ or cambodia/ or cameroon/ or central african republic/ or chad/ or exp china/ or comoros/ or congo/ or cote d'ivoire/ or croatia/ or cuba/ or "democratic republic of the congo"/ or cyprus/ or djibouti/ or dominica/ or dominican republic/ or ecuador/ or egypt/ or el salvador/ or equatorial guinea/ or eritrea/ or eswatini/ or ethiopia/ or fiji/ or gabon/ or gambia/ or "georgia (republic)"/ or ghana/ or grenada/ or guatemala/ or guinea/ or guinea-bissau/ or guyana/ or haiti/ or honduras/ or independent state of samoa/ or exp india/ or indian ocean islands/ or indochina/ or indonesia/ or iran/ or iraq/ or jamaica/ or jordan/ or kazakhstan/ or kenya/ or kosovo/ or kuwait/ or kyrgyzstan/ or laos/ or lebanon/ or liechtenstein/ or lesotho/ or liberia/ or libya/ or madagascar/ or malaysia/ or malawi/ or mali/ or malta/ or mauritania/ or mauritius/ or mekong valley/ or melanesia/ or micronesia/ or monaco/ or mongolia/ or montenegro/ or morocco/ or mozambique/ or myanmar/ or namibia/ or nepal/ or nicaragua/ or niger/ or nigeria/ or oman/ or pakistan/ or palau/ or exp panama/ or papua new guinea/ or paraguay/ or peru/ or philippines/ or qatar/ or "republic of belarus"/ or "republic of north macedonia"/ or romania/ or exp russia/ or rwanda/ or "saint kitts and nevis"/ or saint lucia/ or "saint vincent and the grenadines"/ or "sao tome and principe"/ or saudi arabia/ or serbia/ or sierra leone/ or senegal/ or seychelles/ or singapore/ or somalia/ or south africa/ or south sudan/ or sri lanka/ or sudan/ or suriname/ or syria/ or taiwan/ or tajikistan/ or tanzania/ or thailand/ or timor-leste/ or togo/ or tonga/ or "trinidad and tobago"/ or tunisia/ or turkmenistan/ or uganda/ or ukraine/ or united arab emirates/ or uruguay/ or uzbekistan/ or vanuatu/ or venezuela/ or vietnam/ or west indies/ or yemen/ or zambia/ or zimbabwe/ | 1200781 |
| 51 | "Organisation for Economic Co-Operation and Development"/ | 415 |
| 52 | australasia/ or exp australia/ or austria/ or baltic states/ or belgium/ or exp canada/ or chile/ or colombia/ or costa rica/ or czech republic/ or exp denmark/ or estonia/ or europe/ or finland/ or exp france/ or exp germany/ or greece/ or hungary/ or iceland/ or ireland/ or israel/ or exp italy/ or exp japan/ or korea/ or latvia/ or lithuania/ or luxembourg/ or mexico/ or netherlands/ or new zealand/ or north america/ or exp norway/ or poland/ or portugal/ or exp "republic of korea"/ or "scandinavian and nordic countries"/ or slovakia/ or slovenia/ or spain/ or sweden/ or switzerland/ or turkey/ or exp united kingdom/ or exp united states/ | 3384436 |
| 53 | European Union/ | 17113 |
| 54 | Developed Countries/ | 21083 |
| 55 | or/51-54 | 3399710 |
| 56 | 50 not 55 [OECD search filter NICE 2021] | 1113965 |
| 57 | 49 not 56 [non-OECD countries removed] | 7377 |
| 58 | limit 57 to (comment or editorial or letter or news) | 833 |
| 59 | 57 not 58 [ephemera removed] | 6544 |
| 60 | limit 59 to english language | 6178 |
| 61 | limit 60 to yr="2014 -Current" | 2417 |

**CEA Registry https://cevr.tuftsmedicalcenter.org/databases/cea-registry**

Date searched: 21/02/2022; Records identified: 8

Basic interface

Searched for the following words individually: maternity; midwife; midwifery; midwives; MSW; MSWs in ‘methods’, ‘ratios’ and ‘utilities’

Download 2014+

### Websites and Search Engines search strategies

**King’s Fund**

<http://www.kingsfund.org.uk/>

Date searched: 23/02/2022

Records identified: 64 found, 2 downloaded

Searched ‘publications’ section for single words: midwife; midwifery; midwives; maternity, and published from 2014 onwards. Note – searched for each term separately

**Royal College of Midwives**

<https://www.rcm.org.uk/publications/>

Date searched: 23/02/2022

Records identified: 12 found, 3 downloaded

Searched ‘publications’ section for single words: workforce, staff, staffing, ratio

Limited to reports published 2014+

**Royal College of Paediatrics and Child Health**

<https://www.rcpch.ac.uk/>

Date searched: 23/02/2022

Records identified: 67 found, 11 downloaded

Searched for phrases: midwife staff, midwife staffing, midwife workforce, midwifery staff, midwifery staffing, midwifery workforce, maternity staff, maternity staffing, maternity workforce. Note – searched for each phrase separately.

Limited to reports published 2014+

**Department of Health**

<https://www.gov.uk/>

Date searched: 23/02/2022

Records identified: 181

*Search 1*

searched for the string: maternity midwives midwife midwifery. Note terms are automatically combined with OR boolean logic.

Results limited to About ‘Health and social care’ and About ‘National Health Service’

Results limited to In ‘Guidance and regulation’ or In ‘Policy papers and consultations’

Results limited to Updated ‘after 1 January 2014’

*Search 2*

searched for the string: maternity midwives midwife midwifery. Note terms are automatically combined with OR boolean logic.

Results limited to About ‘Health and social care’ and About ‘National Health Service’

Results limited to In ‘Research & Statistics’

Results limited to Updated ‘after 1 January 2014’

**NHS England**

<http://www.england.nhs.uk/>

Date searched: 23/02/2022

Records identified: 7 found, 2 downloaded

Searched for Keyword: maternity midwifery midwife midwives. Note terms are automatically combined with OR boolean logic.

Results limit to Topic: Safe Staffing, or Topic: Workforce

**NHS Scotland**

<https://www.publications.scot.nhs.uk/>

Date searched: 23/02/2022

Records identified: 2

Searched for keyword phrases: midwife staff, midwife staffing, midwife workforce, midwives staff, midwives staffing, midwives workforce, midwifery staff, midwifery staffing, midwifery workforce, maternity staff, maternity staffing, maternity workforce. Note – searched for each phrase separately.

Limited to reports published 2014+

**Welsh Government Statistics and Research**

<https://gov.wales/statistics-and-research>

Date searched: 22/02/2022

Records identified: 1

Searched for single words: midwife; midwives; maternity staff; maternity staffing, and published from 2014 onwards. Note – searched for each term separately

Limited to Topics: Health & Social Care

Limited to Type: Research

**Scottish Government**

<https://www.gov.scot/publications/>

Date searched: 22/02/2022

Records identified: 0

Searched: (maternity OR midwi*) AND staffing

Limited to Topics: Health & Social Care

Limited to Type: Research and Analysis

Limited to From: 01/01/2014 to 22/02/2022

**NICE Evidence**

<https://www.evidence.nhs.uk/>

Date searched: 22/02/2022

Records identified: 823

Searched: (maternity OR midwi*) AND (staffing OR workforce OR shortage* OR sufficien* OR number*)

Limited to From 01/01/2014- 22/02/2022

Limited to Evidence type: Primary Research OR Systematic Reviews OR Health Technology Assessments OR Economic Evaluations

**Google Scholar**

<http://scholar.google.co.uk/>

searched via Harzing’s Publish or Perish

Date searched: 22/02/2022

Records identified: 48

Searched in Title only: maternity|midwi* AND staffing|workforce|shortage*|sufficien*|number*

Limited to From: 2014- 2022

## S2B: Search Strategies (2023 update)

**CINAHL (EBSCOhost)**

**Date searched: 05/10/2023**

**Records identified: 4180**

| S43 | S42 | 4,180 |
| --- | --- | --- |
| S42 | S30 NOT S40 | 4,243 |
| S41 | S30 NOT S40 | 7,376 |
| S40 | S36 NOT S39 | 402,034 |
| S39 | S37 OR S38 | 1,728,057 |
| S38 | (MH "Australia+") OR (MH "Europe") OR (MH "Austria") OR (MH "Baltic States+") OR (MH "Belgium") OR (MH "Canada+") OR (MH "Chile") OR (MH "Colombia") OR (MH "Costa Rica") OR (MH "Czech Republic") OR (MH "Scandinavia+") OR (MH "France") OR (MH "Germany+") OR (MH "Greece") OR (MH "Hungary") OR (MH "Iceland") OR (MH "Ireland") OR (MH "Italy") OR (MH "Israel") OR (MH "Japan") OR (MH "South Korea") OR (MH "Luxembourg") OR (MH "Mexico") OR (MH "Netherlands") OR (MH "New Zealand") OR (MH "North America") OR (MH "Poland") OR (MH "Portugal") OR (MH "Slovakia") OR (MH "Slovenia") OR (MH "Spain") OR (MH "Switzerland") OR (MH "Turkey") OR (MH "United Kingdom+") OR (MH "United States+") | 1,721,235 |
| S37 | (MH "Developed Countries") or (MH "European Union") or (MH "Organisation for Economic Co-Operation and Development") | 10,607 |
| S36 | (S31 OR S32 OR S33 OR S34 OR S35) | 441,497 |
| S35 | (MH "Africa+") | 99,586 |
| S34 | (MH "Asia, Southeastern+") OR (MH "China+") OR (MH "Hong Kong") OR (MH "Macao") OR (MH "Mongolia") OR (MH "North Korea") OR (MH "Taiwan") OR (MH "Atlantic Islands") OR (MH "Indian Ocean Islands+") OR (MH "Melanesia+") OR (MH "Micronesia+") OR (MH "Polynesia+") | 142,414 |
| S33 | (MH "Bangladesh") OR (MH "Bhutan") OR (MH "India") OR (MH "Yemen") OR (MH "United Arab Emirates") OR (MH "Syria") OR (MH "Saudi Arabia") OR (MH "Qatar") OR (MH "Oman") OR (MH "Lebanon") OR (MH "Kuwait") OR (MH "Jordan") OR (MH "Iraq") OR (MH "Iran") OR (MH "Bahrain") OR (MH "Afghanistan") OR (MH "Nepal") OR (MH "Pakistan") OR (MH "Sri Lanka") OR (MH "Asia, Central+") | 119,652 |
| S32 | (MH "Argentina") OR (MH "Bolivia") OR (MH "Brazil") OR (MH "Ecuador") OR (MH "French Guiana") OR (MH "Guyana") OR (MH "Paraguay") OR (MH "Peru") OR (MH "Suriname") OR (MH "Uruguay") OR (MH "Venezuela") OR (MH "Belize") OR (MH "El Salvador") OR (MH "Guatemala") OR (MH "Honduras") OR (MH "Nicaragua") OR (MH "Panama+") or (MH "West Indies+") | 76,716 |
| S31 | (MH "Albania") OR (MH "Andorra") OR (MH "Armenia") OR (MH "Azerbaijan") or (MH "Byelarus") OR (MH "Bosnia-Herzegovina") OR (MH "Croatia") OR (MH "Bulgaria") OR (MH "Georgia (Republic)") OR (MH "Gibraltar") OR (MH "Liechtenstein") OR (MH "Macedonia (Republic)") OR (MH "Moldova") OR (MH "Monaco") OR (MH "Romania") OR (MH "Russia") OR (MH "San Marino") OR (MH "Serbia") OR (MH "Ukraine") OR (MH "Yugoslavia") | 12,689 |
| S30 | S19 OR S20 OR S21 OR S22 OR S23 OR S24 OR S25 OR S26 OR S27 OR S29 | 8,416 |
| S29 | S7 AND S28 | 5,782 |
| S28 | S8 OR S9 OR S10 OR S11 OR S12 OR S13 OR S14 OR S15 OR S16 OR S17 OR S18 | 316,243 |
| S27 | (MH "Perinatal Care/EC/ST/OG") | 404 |
| S26 | (MH "Delivery Rooms+/EC/OG/ST") | 106 |
| S25 | (MH "Midwives+/EC/ST") | 199 |
| S24 | TI "named midwi*" or AB "named midwi*" | 22 |
| S23 | ( TI (midwi* N3 (level* OR ratio* OR resourc* OR model* OR number* OR mix* OR rota* OR rosta* OR roster* OR schedul* OR overtime OR supervision OR supervisory OR sufficient* OR sufficiency OR adequate* OR adequac* OR target* OR insufficient* OR insufficienc* OR inadequate* OR inadequac* OR short OR shortage* OR efficient* OR efficienc* OR inefficien* OR burnout OR stress OR fatigue OR magnet)) ) OR ( AB (midwi* N3 (level* OR ratio* OR resourc* OR model* OR number* OR mix* OR rota* OR rosta* OR roster* OR schedul* OR overtime OR supervision OR supervisory OR sufficient* OR sufficiency OR adequate* OR adequac* OR target* OR insufficient* OR insufficienc* OR inadequate* OR inadequac* OR short OR shortage* OR efficient* OR efficienc* OR inefficien* OR burnout OR stress OR fatigue OR magnet)) ) | 2,892 |
| S22 | ( TI (maternity N3 worker* N3 (level* OR ratio* OR resourc* OR model* OR number* OR mix* OR rota* OR rosta* OR roster* OR schedul* OR overtime OR supervision OR supervisory OR sufficient* OR sufficiency OR adequate* OR adequac* OR target* OR insufficient* OR insufficienc* OR inadequate* OR inadequac* OR short OR shortage* OR efficient* OR efficienc* OR inefficien* OR burnout OR stress OR fatigue OR magnet)) ) OR ( AB (maternity N3 worker* N3 (level* OR ratio* OR resourc* OR model* OR number* OR mix* OR rota* OR rosta* OR roster* OR schedul* OR overtime OR supervision OR supervisory OR sufficient* OR sufficiency OR adequate* OR adequac* OR target* OR insufficient* OR insufficienc* OR inadequate* OR inadequac* OR short OR shortage* OR efficient* OR efficienc* OR inefficien* OR burnout OR stress OR fatigue OR magnet)) ) | 7 |
| S21 | ( TI (maternity N3 assistant* N3 (level* OR ratio* OR resourc* OR model* OR number* OR mix* OR rota* OR rosta* OR roster* OR schedul* OR overtime OR supervision OR supervisory OR sufficient* OR sufficiency OR adequate* OR adequac* OR target* OR insufficient* OR insufficienc* OR inadequate* OR inadequac* OR short OR shortage* OR efficient* OR efficienc* OR inefficien* OR burnout OR stress OR fatigue OR magnet)) ) OR ( AB (maternity N3 assistant* N3 (level* OR ratio* OR resourc* OR model* OR number* OR mix* OR rota* OR rosta* OR roster* OR schedul* OR overtime OR supervision OR supervisory OR sufficient* OR sufficiency OR adequate* OR adequac* OR target* OR insufficient* OR insufficienc* OR inadequate* OR inadequac* OR short OR shortage* OR efficient* OR efficienc* OR inefficien* OR burnout OR stress OR fatigue OR magnet)) ) | 4 |
| S20 | ( TI (midwi* N3 worker* N3 (level* OR ratio* OR resourc* OR model* OR number* OR mix* OR rota* OR rosta* OR roster* OR schedul* OR overtime OR supervision OR supervisory OR sufficient* OR sufficiency OR adequate* OR adequac* OR target* OR insufficient* OR insufficienc* OR inadequate* OR inadequac* OR short OR shortage* OR efficient* OR efficienc* OR inefficien* OR burnout OR stress OR fatigue OR magnet)) ) OR ( AB (midwi* N3 worker* N3 (level* OR ratio* OR resourc* OR model* OR number* OR mix* OR rota* OR rosta* OR roster* OR schedul* OR overtime OR supervision OR supervisory OR sufficient* OR sufficiency OR adequate* OR adequac* OR target* OR insufficient* OR insufficienc* OR inadequate* OR inadequac* OR short OR shortage* OR efficient* OR efficienc* OR inefficien* OR burnout OR stress OR fatigue OR magnet)) ) | 14 |
| S19 | ( TI (midwi* N3 assistant* N3 (level* OR ratio* OR resourc* OR model* OR number* OR mix* OR rota* OR rosta* OR roster* OR schedul* OR overtime OR supervision OR supervisory OR sufficient* OR sufficiency OR adequate* OR adequac* OR target* OR insufficient* OR insufficienc* OR inadequate* OR inadequac* OR short OR shortage* OR efficient* OR efficienc* OR inefficien* OR burnout OR stress OR fatigue OR magnet)) ) OR ( AB (midwi* N3 assistant* N3 (level* OR ratio* OR resourc* OR model* OR number* OR mix* OR rota* OR rosta* OR roster* OR schedul* OR overtime OR supervision OR supervisory OR sufficient* OR sufficiency OR adequate* OR adequac* OR target* OR insufficient* OR insufficienc* OR inadequate* OR inadequac* OR short OR shortage* OR efficient* OR efficienc* OR inefficien* OR burnout OR stress OR fatigue OR magnet)) ) | 8 |
| S18 | ( TI (skill#mix* OR "skill# mix*" OR staffmix* OR "staff mix*" OR staffing OR understaff* OR manpower OR workload* OR workforce* OR shift OR shiftwork* OR shifts OR overtime OR capacity OR "missed care" OR "missing care" OR "care left undone" OR (hours N2 day) OR (work* N2 hours) OR (hours N2 care) OR caseload OR "case load*" OR turnover OR "turn over" OR FTE OR "full-time equivalent") ) OR ( AB (skill#mix* OR "skill# mix*" OR staffmix* OR "staff mix*" OR staffing OR understaff* OR manpower OR workload* OR workforce* OR shift OR shiftwork* OR shifts OR overtime OR capacity OR "missed care" OR "missing care" OR "care left undone" OR (hours N2 day) OR (work* N2 hours) OR (hours N2 care) OR caseload OR "case load*" OR turnover OR "turn over" OR FTE OR "full-time equivalent") ) | 230,499 |
| S17 | ( TI (staff* N3 (level* OR ratio* OR resourc* OR model* OR number* OR mix* OR rota* OR rosta* OR roster* OR schedul* OR overtime OR supervision OR supervisory OR sufficient* OR sufficiency OR adequate* OR adequac* OR target* OR insufficient* OR insufficienc* OR inadequate* OR inadequac* OR short OR shortage* OR efficient* OR efficienc* OR inefficien* OR burnout OR stress OR fatigue OR magnet)) ) OR ( AB (staff* N3 (level* OR ratio* OR resourc* OR model* OR number* OR mix* OR rota* OR rosta* OR roster* OR schedul* OR overtime OR supervision OR supervisory OR sufficient* OR sufficiency OR adequate* OR adequac* OR target* OR insufficient* OR insufficienc* OR inadequate* OR inadequac* OR short OR shortage* OR efficient* OR efficienc* OR inefficien* OR burnout OR stress OR fatigue OR magnet)) ) | 20,414 |
| S16 | (MH "Stress, Occupational") | 19,725 |
| S15 | (MH "Nursing Labor Supply") | 5,847 |
| S14 | (MH "Workforce") | 14,860 |
| S13 | (MH "Magnet Hospitals") | 2,639 |
| S12 | (MH "Personnel Turnover") | 6,138 |
| S11 | (MH "Burnout, Professional+") | 16,407 |
| S10 | (MH "Personnel Shortage+") | 18,170 |
| S9 | (MH "Workload") | 18,693 |
| S8 | (MH "Personnel Staffing and Scheduling+") | 34,482 |
| S7 | S1 OR S2 OR S3 OR S4 OR S5 OR S6 | 46,551 |
| S6 | TI (msw* NOT ("municipal solid" or "male sex work*" or "medical social work*")) OR AB (msw* NOT ("municipal solid" or "male sex work*" or "medical social work*")) | 886 |
| S5 | TI (maternity N3 assistant*) or AB (maternity N3 assistant*) | 54 |
| S4 | TI (maternity N3 staff*) or AB (maternity N3 staff*) | 283 |
| S3 | TI (maternity N3 worker*) or AB (maternity N3 worker*) | 171 |
| S2 | TI (midwif* or midwiv*) or AB (midwif* or midwiv*) | 38,047 |
| S1 | (MH "Midwives+") OR (MH "Midwifery Service+") | 18,146 |

**Cochrane Database of Systematic Reviews Issue 10 of 12, October 2023 and**

**Cochrane Central Register of Controlled Trials Issue 10 of 12, October 2023 search simultaneously.**

**Date searched: 05/10/2023**

**Records identified: 371**

ID Search Hits

#1 MeSH descriptor: [Midwifery] this term only 472

#2 MeSH descriptor: [Nurse Midwives] this term only 127

#3 (midwif* or midwiv*):ti,ab 2788

#4 (maternity near/4 worker*):ti,ab 11

#5 (maternity near/4 staff*):ti,ab 36

#6 (maternity near/4 assistant*):ti,ab 8

#7 (midwi* near/4 assistant*):ti,ab 22

#8 (midwi* near/4 staff*):ti,ab 65

#9 (midwi* near/4 worker*):ti,ab 58

#10 msw* not ("municipal solid" or "municipal solid" or (male NEXT sex NEXT work*) or (medical NEXT social NEXT work*)):ti,ab 341

#11 #1 or #2 or #3 or #4 or #5 or #6 or #7 or #8 or #9 or #10 3304

#12 (staff* near/4 (level* or ratio* or resourc* or model* or number* or mix* or rota* or rosta* or

roster* or schedul* or overtime or supervision or supervisory or sufficient* or sufficiency or adequate*

or adequac* or target* or insufficient* or insufficienc* or inadequate* or inadequac* or short or

shortage* or efficient* or efficienc* or inefficien* or burnout or stress or fatigue or magnet)):ti,ab 2145

#13 (skill?mix* or (skill? NEXT mix*)):ti,ab 41

#14 (staffmix* or (staff NEXT mix*)):ti,ab 6

#15 staffing:ti,ab 642

#16 understaff*:ti,ab 32

#17 (under NEXT staff*):ti,ab 18

#18 MeSH descriptor: [Personnel Staffing and Scheduling] explode all trees 1020

#19 MeSH descriptor: [Health Workforce] this term only 35

#20 manpower:ti,ab 193

#21 (workload* or workforce* or shift or shiftwork* or shifts or overtime or capacity):ti,ab 58294

#22 MeSH descriptor: [Workload] this term only 714

#23 ("missed care" or "missing care"):ti,ab 4

#24 "care left undone":ti,ab 1

#25 (hours near/3 day):ti,ab 5930

#26 (work* near/3 hours):ti,ab 1308

#27 (hours near/3 care):ti,ab 572

#28 (caseload or (case NEXT load*)):ti,ab 330

#29 (turnover or "turn over"):ti,ab 6086

#30 (FTE or "full-time equivalent"):ti,ab 84

#31 MeSH descriptor: [Occupational Stress] explode all trees 682

#32 #12 or #13 or #14 or #15 or #16 or #17 or #18 or #19 or #20 or #21 or #22 or #23 or #24 or #25

or #26 or #27 or #28 or #29 or #30 or #31 74751

#33 #11 and #32 269

#34 (midwi* near/4 assistant* near/4 (level* or ratio* or resourc* or model* or number* or mix* or

rota* or rosta* or roster* or schedul* or overtime or supervision or supervisory or sufficient* or

sufficiency or adequate* or adequac* or target* or insufficient* or insufficienc* or inadequate* or

inadequac* or short or shortage* or efficient* or efficienc* or inefficien* or burnout or stress or

fatigue or magnet)):ti,ab 0

#35 (midwi* near/4 worker* near/4 (level* or ratio* or resourc* or model* or number* or mix* or

rota* or rosta* or roster* or schedul* or overtime or supervision or supervisory or sufficient* or

sufficiency or adequate* or adequac* or target* or insufficient* or insufficienc* or inadequate* or

inadequac* or short or shortage* or efficient* or efficienc* or inefficien* or burnout or stress or

fatigue or magnet)):ti,ab 0

#36 (maternity near/4 assistant* near/4 (level* or ratio* or resourc* or model* or number* or mix*

or rota* or rosta* or roster* or schedul* or overtime or supervision or supervisory or sufficient* or

sufficiency or adequate* or adequac* or target* or insufficient* or insufficienc* or inadequate* or inadequac* or short or shortage* or efficient* or efficienc* or inefficien* or burnout or stress or

fatigue or magnet)):ti,ab 1

#37 (maternity near/4 worker* near/4 (level* or ratio* or resourc* or model* or number* or mix* or

rota* or rosta* or roster* or schedul* or overtime or supervision or supervisory or sufficient* or

sufficiency or adequate* or adequac* or target* or insufficient* or insufficienc* or inadequate* or

inadequac* or short or shortage* or efficient* or efficienc* or inefficien* or burnout or stress or

fatigue or magnet)):ti,ab 1

#38 (midwi* near/4 (level* or ratio* or resourc* or model* or number* or mix* or rota* or rosta* or

roster* or schedul* or overtime or supervision or supervisory or sufficient* or sufficiency or adequate*

or adequac* or target* or insufficient* or insufficienc* or inadequate* or inadequac* or short or

shortage* or efficient* or efficienc* or inefficien* or burnout or stress or fatigue or magnet)):ti,ab 206

#39 (named NEXT midwi*):ti,ab 6

#40 #33 or #34 or #35 or #36 or #37 or #38 or #39 437

#41 MeSH descriptor: [Perinatal Care] explode all trees and with qualifier(s): [standards - ST, economics - EC, organization & administration - OG] 160

#42 MeSH descriptor: [Delivery Rooms] this term only and with qualifier(s): [standards - ST, economics - EC, organization & administration - OG] 17

#43 MeSH descriptor: [Birthing Centers] this term only and with qualifier(s): [standards - ST, economics - EC, organization & administration - OG] 9

#44 MeSH descriptor: [Midwifery] this term only and with qualifier(s): [economics - EC, organization & administration - OG] 36

#45 MeSH descriptor: [Nurse Midwives] this term only and with qualifier(s): [economics - EC, organization & administration - OG] 18

#46 #40 or #41 or #42 or #43 or #44 or #45 636

#47 #40 or #41 or #42 or #43 or #44 or #45 with Publication Year from 2014 to 2023, with Cochrane Library publication date Between Jan 2014 and Dec 2023

**ECONLit (EBSCOhost)**

Date searched: 05/10/2023

Records identified: 32

| S18 | S17 [Limiters - Published Date: 20140101-20231231] | 32 |
| --- | --- | --- |
| S17 | S9 OR S10 OR S11 OR S12 OR S13 OR S14 OR S16 | 44 |
| S16 | S6 AND S15 | 34 |
| S15 | S7 OR S8 | 83,495 |
| S14 | TI "named midwi*" or AB "named midwi*" | 1 |
| S13 | ( TI (midwi* N3 (level* OR ratio* OR resourc* OR model* OR number* OR mix* OR rota* OR rosta* OR roster* OR schedul* OR overtime OR supervision OR supervisory OR sufficient* OR sufficiency OR adequate* OR adequac* OR target* OR insufficient* OR insufficienc* OR inadequate* OR inadequac* OR short OR shortage* OR efficient* OR efficienc* OR inefficien* OR burnout OR stress OR fatigue OR magnet)) ) OR ( AB (midwi* N3 (level* OR ratio* OR resourc* OR model* OR number* OR mix* OR rota* OR rosta* OR roster* OR schedul* OR overtime OR supervision OR supervisory OR sufficient* OR sufficiency OR adequate* OR adequac* OR target* OR insufficient* OR insufficienc* OR inadequate* OR inadequac* OR short OR shortage* OR efficient* OR efficienc* OR inefficien* OR burnout OR stress OR fatigue OR magnet)) ) | 17 |
| S12 | ( TI (maternity N3 worker* N3 (level* OR ratio* OR resourc* OR model* OR number* OR mix* OR rota* OR rosta* OR roster* OR schedul* OR overtime OR supervision OR supervisory OR sufficient* OR sufficiency OR adequate* OR adequac* OR target* OR insufficient* OR insufficienc* OR inadequate* OR inadequac* OR short OR shortage* OR efficient* OR efficienc* OR inefficien* OR burnout OR stress OR fatigue OR magnet)) ) OR ( AB (maternity N3 worker* N3 (level* OR ratio* OR resourc* OR model* OR number* OR mix* OR rota* OR rosta* OR roster* OR schedul* OR overtime OR supervision OR supervisory OR sufficient* OR sufficiency OR adequate* OR adequac* OR target* OR insufficient* OR insufficienc* OR inadequate* OR inadequac* OR short OR shortage* OR efficient* OR efficienc* OR inefficien* OR burnout OR stress OR fatigue OR magnet)) ) | 0 |
| S11 | ( TI (maternity N3 assistant* N3 (level* OR ratio* OR resourc* OR model* OR number* OR mix* OR rota* OR rosta* OR roster* OR schedul* OR overtime OR supervision OR supervisory OR sufficient* OR sufficiency OR adequate* OR adequac* OR target* OR insufficient* OR insufficienc* OR inadequate* OR inadequac* OR short OR shortage* OR efficient* OR efficienc* OR inefficien* OR burnout OR stress OR fatigue OR magnet)) ) OR ( AB (maternity N3 assistant* N3 (level* OR ratio* OR resourc* OR model* OR number* OR mix* OR rota* OR rosta* OR roster* OR schedul* OR overtime OR supervision OR supervisory OR sufficient* OR sufficiency OR adequate* OR adequac* OR target* OR insufficient* OR insufficienc* OR inadequate* OR inadequac* OR short OR shortage* OR efficient* OR efficienc* OR inefficien* OR burnout OR stress OR fatigue OR magnet)) ) | 0 |
| S10 | ( TI (midwi* N3 worker* N3 (level* OR ratio* OR resourc* OR model* OR number* OR mix* OR rota* OR rosta* OR roster* OR schedul* OR overtime OR supervision OR supervisory OR sufficient* OR sufficiency OR adequate* OR adequac* OR target* OR insufficient* OR insufficienc* OR inadequate* OR inadequac* OR short OR shortage* OR efficient* OR efficienc* OR inefficien* OR burnout OR stress OR fatigue OR magnet)) ) OR ( AB (midwi* N3 worker* N3 (level* OR ratio* OR resourc* OR model* OR number* OR mix* OR rota* OR rosta* OR roster* OR schedul* OR overtime OR supervision OR supervisory OR sufficient* OR sufficiency OR adequate* OR adequac* OR target* OR insufficient* OR insufficienc* OR inadequate* OR inadequac* OR short OR shortage* OR efficient* OR efficienc* OR inefficien* OR burnout OR stress OR fatigue OR magnet)) ) | 0 |
| S9 | ( TI (midwi* N3 assistant* N3 (level* OR ratio* OR resourc* OR model* OR number* OR mix* OR rota* OR rosta* OR roster* OR schedul* OR overtime OR supervision OR supervisory OR sufficient* OR sufficiency OR adequate* OR adequac* OR target* OR insufficient* OR insufficienc* OR inadequate* OR inadequac* OR short OR shortage* OR efficient* OR efficienc* OR inefficien* OR burnout OR stress OR fatigue OR magnet)) ) OR ( AB (midwi* N3 assistant* N3 (level* OR ratio* OR resourc* OR model* OR number* OR mix* OR rota* OR rosta* OR roster* OR schedul* OR overtime OR supervision OR supervisory OR sufficient* OR sufficiency OR adequate* OR adequac* OR target* OR insufficient* OR insufficienc* OR inadequate* OR inadequac* OR short OR shortage* OR efficient* OR efficienc* OR inefficien* OR burnout OR stress OR fatigue OR magnet)) ) | 0 |
| S8 | ( TI (skill#mix* OR "skill# mix*" OR staffmix* OR "staff mix*" OR staffing OR understaff* OR manpower OR workload* OR workforce* OR shift OR shiftwork* OR shifts OR overtime OR capacity OR "missed care" OR "missing care" OR "care left undone" OR (hours N2 day) OR (work* N2 hours) OR (hours N2 care) OR caseload OR "case load*" OR turnover OR "turn over" OR FTE OR "full-time equivalent") ) OR ( AB (skill#mix* OR "skill# mix*" OR staffmix* OR "staff mix*" OR staffing OR understaff* OR manpower OR workload* OR workforce* OR shift OR shiftwork* OR shifts OR overtime OR capacity OR "missed care" OR "missing care" OR "care left undone" OR (hours N2 day) OR (work* N2 hours) OR (hours N2 care) OR caseload OR "case load*" OR turnover OR "turn over" OR FTE OR "full-time equivalent") ) | 82,986 |
| S7 | ( TI (staff* N3 (level* OR ratio* OR resourc* OR model* OR number* OR mix* OR rota* OR rosta* OR roster* OR schedul* OR overtime OR supervision OR supervisory OR sufficient* OR sufficiency OR adequate* OR adequac* OR target* OR insufficient* OR insufficienc* OR inadequate* OR inadequac* OR short OR shortage* OR efficient* OR efficienc* OR inefficien* OR burnout OR stress OR fatigue OR magnet)) ) OR ( AB (staff* N3 (level* OR ratio* OR resourc* OR model* OR number* OR mix* OR rota* OR rosta* OR roster* OR schedul* OR overtime OR supervision OR supervisory OR sufficient* OR sufficiency OR adequate* OR adequac* OR target* OR insufficient* OR insufficienc* OR inadequate* OR inadequac* OR short OR shortage* OR efficient* OR efficienc* OR inefficien* OR burnout OR stress OR fatigue OR magnet)) ) | 925 |
| S6 | S1 OR S2 OR S3 OR S4 OR S5 | 171 |
| S5 | TI (msw* NOT "municipal solid") OR AB (msw* NOT "municipal solid") | 18 |
| S4 | TI (maternity N3 assistant*) or AB (maternity N3 assistant*) | 25 |
| S3 | TI (maternity N3 staff*) or AB (maternity N3 staff*) | 5 |
| S2 | TI (maternity N3 worker*) or AB (maternity N3 worker*) | 9 |
| S1 | TI (midwif* or midwiv*) or AB (midwif* or midwiv*) | 140 |

**Embase Classic+Embase (Ovid) 1947 to 2022 February 18**

**Date searched: 04/10/2023**

**Records identified: 980**

**Embase Classic+Embase (Ovid) <1947 to 2023 October 03>**

1 exp midwife/ 37897

2 (midwif* or midwiv*).tw. 34253

3 (maternity adj3 worker*).tw. 141

4 (maternity adj3 staff*).tw. 506

5 (maternity adj3 assistant*).tw. 45

6 (msw* not ("municipal solid" or "male sex work*" or "medical social work*")).tw. 2803

7 or/1-6 [Maternity staff] 51188

8 (staff* adj3 (level* or ratio* or resourc* or model* or number* or mix* or rota* or rosta* or roster* or schedul* or overtime or supervision or supervisory or sufficient* or sufficiency or adequate* or adequac* or target* or insufficient* or insufficienc* or inadequate* or inadequac* or short or shortage* or efficient* or efficienc* or inefficien* or burnout or stress or fatigue or magnet)).tw. 31723

9 (skill?mix* or "skill? mix*").tw. 1568

10 (staffmix* or "staff mix*").tw. 119

11 staffing.tw. 24845

12 understaff*.tw. 1081

13 "under staff*".tw. 121

14 skill mix/ 503

15 personnel management/ 60763

16 exp health care personnel management/ 4055

17 exp workforce/ 15856

18 manpower planning/ 949

19 work schedule/ 10195

20 workload/ 55698

21 working time/ 11810

22 exp shift worker/ 6730

23 manpower.tw. 11123

24 (workload* or workforce* or shift or shiftwork* or shifts or overtime or capacity).tw. 1348138

25 magnet hospital/ 54

26 burnout/ or professional burnout/ 29570

27 exp personnel shortage/ 4239

28 ("missed care" or "missing care").tw. 345

29 "care left undone".tw. 51

30 (hours adj2 day).tw. 16397

31 (work* adj2 hours).tw. 19216

32 (hours adj2 care).tw. 2284

33 (caseload or "case load*").tw. 7352

34 (turnover or "turn over").tw. 138951

35 (FTE or "full-time equivalent").tw. 2778

36 exp job stress/ 16011

37 or/8-36 1680899

38 7 and 37 5267

39 (midwi* adj3 assistant* adj3 (level* or ratio* or resourc* or model* or number* or mix* or rota* or rosta* or roster* or schedul* or overtime or supervision or supervisory or sufficient* or sufficiency or adequate* or adequac* or target* or insufficient* or insufficienc* or inadequate* or inadequac* or short or shortage* or efficient* or efficienc* or inefficien* or burnout or stress or fatigue or magnet)).tw. 7

40 (midwi* adj3 worker* adj3 (level* or ratio* or resourc* or model* or number* or mix* or rota* or rosta* or roster* or schedul* or overtime or supervision or supervisory or sufficient* or sufficiency or adequate* or adequac* or target* or insufficient* or insufficienc* or inadequate* or inadequac* or short or shortage* or efficient* or efficienc* or inefficien* or burnout or stress or fatigue or magnet)).tw. 4

41 (maternity adj3 assistant* adj3 (level* or ratio* or resourc* or model* or number* or mix* or rota* or rosta* or roster* or schedul* or overtime or supervision or supervisory or sufficient* or sufficiency or adequate* or adequac* or target* or insufficient* or insufficienc* or inadequate* or inadequac* or short or shortage* or efficient* or efficienc* or inefficien* or burnout or stress or fatigue or magnet)).tw. 0

42 (maternity adj3 worker* adj3 (level* or ratio* or resourc* or model* or number* or mix* or rota* or rosta* or roster* or schedul* or overtime or supervision or supervisory or sufficient* or sufficiency or adequate* or adequac* or target* or insufficient* or insufficienc* or inadequate* or inadequac* or short or shortage* or efficient* or efficienc* or inefficien* or burnout or stress or fatigue or magnet)).tw. 6

43 (midwi* adj3 (level* or ratio* or resourc* or model* or number* or mix* or rota* or rosta* or roster* or schedul* or overtime or supervision or supervisory or sufficient* or sufficiency or adequate* or adequac* or target* or insufficient* or insufficienc* or inadequate* or inadequac* or short or shortage* or efficient* or efficienc* or inefficien* or burnout or stress or fatigue or magnet)).tw. 2025

44 or/39-43 2036

45 "named midwi*".tw. 18

46 38 or 44 or 45 [Maternity Staff issues] 6681

47 nonhuman/ not exp human/ 5297921

48 46 not 47 [Animal studies removed] 6659

49 afghanistan/ or africa/ or "africa south of the sahara"/ or albania/ or algeria/ or andorra/ or angola/ or argentina/ or "antigua and barbuda"/ or armenia/ or exp azerbaijan/ or bahamas/ or bahrain/ or bangladesh/ or barbados/ or belarus/ or belize/ or benin/ or bhutan/ or bolivia/ or borneo/ or exp "bosnia and herzegovina"/ or botswana/ or exp brazil/ or brunei darussalam/ or bulgaria/ or burkina faso/ or burundi/ or cambodia/ or cameroon/ or cape verde/ or central africa/ or central african republic/ or chad/ or exp china/ or comoros/ or congo/ or cook islands/ or coted'ivoire/ or croatia/ or cuba/ or cyprus/ or democratic republic congo/ or djibouti/ or dominica/ or dominican republic/ or ecuador/ or el salvador/ or egypt/ or equatorial guinea/ or eritrea/ or eswatini/ or ethiopia/ or exp "federated states of micronesia"/ or fiji/ or gabon/ or gambia/ or exp "georgia (republic)"/ or ghana/ or grenada/ or guatemala/ or guinea/ or guinea-bissau/ or guyana/ or haiti/ or honduras/ or exp india/ or exp indonesia/ or iran/ or exp iraq/ or jamaica/ or jordan/ or kazakhstan/ or kenya/ or kiribati/ or kosovo/ or kuwait/ or kyrgyzstan/ or laos/ or lebanon/ or liechtenstein/ or lesotho/ or liberia/ or libyan arab jamahiriya/ or madagascar/ or malawi/ or exp malaysia/ or maldives/ or mali/ or malta/ or mauritania/ or mauritius/ or melanesia/ or moldova/ or monaco/ or mongolia/ or "montenegro (republic)"/ or morocco/ or mozambique/ or myanmar/ or namibia/ or nauru/ or nepal/ or nicaragua/ or niger/ or nigeria/ or niue/ or north africa/ or oman/ or exp pakistan/ or palau/ or palestine/ or panama/ or papua new guinea/ or paraguay/ or peru/ or philippines/ or polynesia/ or qatar/ or "republic of north macedonia"/ or romania/ or exp russian federation/ or rwanda/ or sahel/ or "saint kitts and nevis"/ or "saint lucia"/ or "saint vincent and the grenadines"/ or saudi arabia/ or senegal/ or exp serbia/ or seychelles/ or sierra leone/ or singapore/ or "sao tome and principe"/ or solomon islands/ or exp somalia/ or south africa/ or south asia/ or south sudan/ or exp southeast asia/ or sri lanka/ or sudan/ or suriname/ or syrian arab republic/ or taiwan/ or tajikistan/ or tanzania/ or thailand/ or timor-leste/ or togo/ or tonga/ or "trinidad andtobago"/ or tunisia/ or turkmenistan/ or tuvalu/ or uganda/ or exp ukraine/ or exp united arab emirates/ or uruguay/ or exp uzbekistan/ or vanuatu/ or venezuela/ or viet nam/ or western sahara/ or yemen/ or zambia/ or zimbabwe/ 1777598

50 "organisation for economic co-operation and development"/ 2677

51 exp australia/ or "australia and new zealand"/ or austria/ or baltic states/ or exp belgium/ or exp canada/ or chile/ or colombia/ or costa rica/ or czech republic/ or denmark/ or estonia/ or europe/ or exp finland/ or exp france/ or exp germany/ or greece/ or hungary/ or iceland/ or ireland/ or israel/ or exp italy/ or japan/ or korea/ or latvia/ or lithuania/ or luxembourg/ or exp mexico/ or netherlands/ or new zealand/ or north america/ or exp norway/ or poland/ or exp portugal/ or scandinavia/ or sweden/ or slovakia/ or slovenia/ or south korea/ or exp spain/ or switzerland/ or exp united kingdom/ or "turkey (republic)"/ or exp united states/ or western europe/ 4041844

52 european union/ 31552

53 developed country/ 36192

54 or/50-53 4075720

55 49 not 54 [NICE Filter 2021 Non-OECD countries] 1615334

56 48 not 55 [non-OECD countries removed] 5332

57 limit 56 to (editorial or letter or note) 286

58 56 not 57 [ephemera removed] 5046

59 limit 58 to english language 4846

60 limit 59 to yr="2014 -Current" 2852

61 limit 60 to embase 980

**Epistemonikos** [**https://www.epistemonikos.org/**](https://www.epistemonikos.org/)

**Date searched: 10/10/2023**

**Total records identified: 910**

Search 1

TI/AB Midwif* OR Midwiv* OR (maternity AND (worker* OR staff OR assistant*)) OR (msw* NOT ("municipal solid" OR "male sex worker*"))

AND

TI/AB "skill* mix*" OR "staff mix*" OR staffing OR understaff* OR manpower OR workload* OR workforce* OR shift OR shiftwork* OR shifts OR overtime OR capacity OR "missed care" OR "missing care" OR "working hours" OR "hours per day" OR "hours of care" OR caseload OR "case load*" OR turnover OR "turn over" OR FTE OR "full-time equivalent" OR (staff AND (level* OR ratio* OR resourc* OR model* OR number* OR rota* OR rosta* OR roster* OR schedul* OR overtime OR supervision OR supervisory OR sufficient* OR sufficiency OR adequate* OR adequac* OR target* OR insufficient* OR insufficienc* OR inadequate* OR inadequac* OR short OR shortage* OR efficient* OR efficienc* OR inefficien* OR burnout OR stress OR fatigue OR magnet))

Limit 2014+ = 681

Search 2

TI/AB (Midwif* OR midwiv* AND (worker* OR assistant*))

AND

TI/AB level* OR ratio* OR resourc* OR model* OR number* OR mix* OR rota* OR rosta* OR roster* OR schedul* OR overtime OR supervision OR supervisory OR sufficient* OR sufficiency OR adequate* OR adequac* OR target* OR insufficient* OR insufficienc* OR inadequate* OR inadequac* OR short OR shortage* OR efficient* OR efficienc* OR inefficien* OR burnout OR stress OR fatigue OR magnet

Limit 2014+ = 242

Search 3

TI/AB "named midwi*"

= 0

**Total once OR’s in builder = 838**

**HMIC Health Management Information Consortium <1979 to July 2023>**

**Date searched: 04/10/2023**

**Records identified: 140**

1 exp midwives/ 2339

2 (midwif* or midwiv*).tw. 4633

3 midwifery/ 672

4 midwifery services/ 542

5 (maternity adj3 worker*).tw. 22

6 (maternity adj3 staff*).tw. 54

7 (maternity adj3 assistant*).tw. 5

8 (msw* not ("municipal solid" or "male sex work*" or "medical social work*")).tw. 20

9 maternity support workers/ 5

10 or/1-9 [Maternity Staff] 5361

11 exp staffing levels/ 490

12 skill mix/ 627

13 staff allocation/ 67

14 exp workload/ 1486

15 workload management/ or workload measurement/ 71

16 workload analysis/ 24

17 staff turnover/ 274

18 occupational stress/ 1362

19 (staff* adj3 (level* or ratio* or resourc* or model* or number* or mix* or rota* or rosta* or roster* or schedul* or overtime or supervision or supervisory or sufficient* or sufficiency or adequate* or adequac* or target* or insufficient* or insufficienc* or inadequate* or inadequac* or short or shortage* or efficient* or efficienc* or inefficien* or burnout or stress or fatigue or magnet)).tw. 3815

20 (skill?mix* or "skill? mix*").tw. 692

21 (staffmix* or "staff mix*").tw. 21

22 staffing.tw. 3264

23 understaff*.tw. 89

24 "under staff*".tw. 12

25 manpower.tw. 1162

26 workforce/ or workforce planning/ 6859

27 (workload* or workforce* or shift or shiftwork* or shifts or overtime or capacity).tw. 16027

28 ("missed care" or "missing care").tw. 10

29 "care left undone".tw. 6

30 (hours adj2 day).tw. 183

31 (work* adj2 hours).tw. 1028

32 (hours adj2 care).tw. 432

33 (caseload or "case load*").tw. 484

34 (turnover or "turn over").tw. 765

35 (FTE or "full-time equivalent").tw. 92

36 or/11-35 28992

37 10 and 36 716

38 (midwi* adj3 assistant* adj3 (level* or ratio* or resourc* or model* or number* or mix* or rota* or rosta* or roster* or schedul* or overtime or supervision or supervisory or sufficient* or sufficiency or adequate* or adequac* or target* or insufficient* or insufficienc* or inadequate* or inadequac* or short or shortage* or efficient* or efficienc* or inefficien* or burnout or stress or fatigue or magnet)).tw. 0

39 (midwi* adj3 worker* adj3 (level* or ratio* or resourc* or model* or number* or mix* or rota* or rosta* or roster* or schedul* or overtime or supervision or supervisory or sufficient* or sufficiency or adequate* or adequac* or target* or insufficient* or insufficienc* or inadequate* or inadequac* or short or shortage* or efficient* or efficienc* or inefficien* or burnout or stress or fatigue or magnet)).tw. 1

40 (maternity adj3 assistant* adj3 (level* or ratio* or resourc* or model* or number* or mix* or rota* or rosta* or roster* or schedul* or overtime or supervision or supervisory or sufficient* or sufficiency or adequate* or adequac* or target* or insufficient* or insufficienc* or inadequate* or inadequac* or short or shortage* or efficient* or efficienc* or inefficien* or burnout or stress or fatigue or magnet)).tw. 0

41 (maternity adj3 worker* adj3 (level* or ratio* or resourc* or model* or number* or mix* or rota* or rosta* or roster* or schedul* or overtime or supervision or supervisory or sufficient* or sufficiency or adequate* or adequac* or target* or insufficient* or insufficienc* or inadequate* or inadequac* or short or shortage* or efficient* or efficienc* or inefficien* or burnout or stress or fatigue or magnet)).tw. 1

42 (midwi* adj3 (level* or ratio* or resourc* or model* or number* or mix* or rota* or rosta* or roster* or schedul* or overtime or supervision or supervisory or sufficient* or sufficiency or adequate* or adequac* or target* or insufficient* or insufficienc* or inadequate* or inadequac* or short or shortage* or efficient* or efficienc* or inefficien* or burnout or stress or fatigue or magnet)).tw. 259

43 or/38-42 260

44 "named midwi*".tw. 11

45 37 or 43 or 44 [Maternity Staffing issues] 902

46 limit 45 to yr="2014 -Current" 140

**International HTA Database (INAHTA) https://database.inahta.org/**

**Date searched: 05/10/2023**

**Records identified:** **16**

19 #18 year limited to 2014 – 2023 16

18 #17 AND #5 40

17 #16 OR #15 OR #14 OR #13 OR #12 OR #11 OR #10 OR #9 OR #8 OR #7 OR #6 8534

16 "named midwi*" 0

15 turnover or "turn over" OR FTE or "full-time equivalent" or level* or ratio* or resourc* or model* or number* or rota* or rosta* or roster* or schedul* or overtime or supervision or supervisory or sufficient* or sufficiency or adequate* or adequac* or target* or insufficient* or insufficienc* or inadequate* or inadequac* or short or shortage* or efficient* or efficienc* or inefficien* or burnout or stress or fatigue or magnet 8436

14 "missed care" or "missing care" or "working hours" or "hours per day" or "hours of care" or caseload or "case load*" 16

13 manpower OR workload* OR workforce* OR shift OR shiftwork* OR shifts OR overtime OR capacity 339

12 skillmix* OR "skill* mix*" OR staffmix* OR "staff mix*" OR staffing OR understaff* 45

11 "Work-Life Balance"[mhe] 0

10 "Occupational Stress"[mhe] 1

9 "Burnout, Professional"[mhe] 8

8 "Workload"[mhe] 16

7 "Health Workforce"[mhe] 4

6 "Personnel Staffing and Scheduling Information Systems"[mhe] 2

5 #4 OR #3 OR #2 OR #1 56

4 maternity AND (worker* OR staff* OR assistant*) 7

3 midwif* OR midwiv* 50

2 "Nurse Midwives"[mhe] 2

1 "Midwifery"[mhe] 15

**Maternity & Infant Care Database (MIDIRS) <1971 to September 26, 2023>**

**Date searched: 04/10/2023**

**Records identified: 1699**

1 midwi*.tw. 39833

2 (maternity adj3 worker*).tw. 247

3 (maternity adj3 staff*).tw. 377

4 (maternity adj3 assistant*).tw. 88

5 (msw* not ("municipal solid" or "male sex work*" or "medical social work*")).tw. 64

6 or/1-5 [Maternity staff] 40090

7 (staff* adj3 (level* or ratio* or resourc* or model* or number* or mix* or rota* or rosta* or roster* or schedul* or overtime or supervision or supervisory or sufficient* or sufficiency or adequate* or adequac* or target* or insufficient* or insufficienc* or inadequate* or inadequac* or short or shortage* or efficient* or efficienc* or inefficien* or burnout or stress or fatigue or magnet)).tw. 2565

8 (skill?mix* or "skill? mix*").tw. 121

9 (staffmix* or "staff mix*").tw. 3

10 staffing.tw. 1629

11 understaff*.tw. 81

12 "under staff*".tw. 7

13 manpower.tw. 251

14 (workload* or workforce* or shift or shiftwork* or shifts or overtime or capacity).tw. 7052

15 ("missed care" or "missing care").tw. 13

16 "care left undone".tw. 1

17 (hours adj2 day).tw. 236

18 (work* adj2 hours).tw. 373

19 (hours adj2 care).tw. 33

20 (caseload or "case load*").tw. 470

21 (turnover or "turn over").tw. 638

22 (FTE or "full-time equivalent").tw. 55

23 or/7-22 10920

24 6 and 23 3817

25 (midwi* adj3 assistant* adj3 (level* or ratio* or resourc* or model* or number* or mix* or rota* or rosta* or roster* or schedul* or overtime or supervision or supervisory or sufficient* or sufficiency or adequate* or adequac* or target* or insufficient* or insufficienc* or inadequate* or inadequac* or short or shortage* or efficient* or efficienc* or inefficien* or burnout or stress or fatigue or magnet)).tw. 1

26 (midwi* adj3 worker* adj3 (level* or ratio* or resourc* or model* or number* or mix* or rota* or rosta* or roster* or schedul* or overtime or supervision or supervisory or sufficient* or sufficiency or adequate* or adequac* or target* or insufficient* or insufficienc* or inadequate* or inadequac* or short or shortage* or efficient* or efficienc* or inefficien* or burnout or stress or fatigue or magnet)).tw. 2

27 (maternity adj3 assistant* adj3 (level* or ratio* or resourc* or model* or number* or mix* or rota* or rosta* or roster* or schedul* or overtime or supervision or supervisory or sufficient* or sufficiency or adequate* or adequac* or target* or insufficient* or insufficienc* or inadequate* or inadequac* or short or shortage* or efficient* or efficienc* or inefficien* or burnout or stress or fatigue or magnet)).tw. 2

28 (maternity adj3 worker* adj3 (level* or ratio* or resourc* or model* or number* or mix* or rota* or rosta* or roster* or schedul* or overtime or supervision or supervisory or sufficient* or sufficiency or adequate* or adequac* or target* or insufficient* or insufficienc* or inadequate* or inadequac* or short or shortage* or efficient* or efficienc* or inefficien* or burnout or stress or fatigue or magnet)).tw. 10

29 (midwi* adj3 (level* or ratio* or resourc* or model* or number* or mix* or rota* or rosta* or roster* or schedul* or overtime or supervision or supervisory or sufficient* or sufficiency or adequate* or adequac* or target* or insufficient* or insufficienc* or inadequate* or inadequac* or short or shortage* or efficient* or efficienc* or inefficien* or burnout or stress or fatigue or magnet)).tw. 2918

30 or/25-29 2929

31 "named midwi*".tw. 82

32 24 or 30 or 31 5964

33 limit 32 to (commentary or correspondence or editorial or news or news item or news release) 1065

34 32 not 33 [Ephemera removed] 4899

35 limit 34 to yr="2014 -Current" 1967

36 (afghanistan* or africa* or albania* or algeria* or andorra* or angola* or argentin* or Antigua* or barbuda* or armenia* or azerbaijan* or Bahamas* or bahrain* or bangladesh* or barbados* or belarus* or belize* or benin* or bhutan* or bolivia* or borneo* or bosnia* or herzegovin* or botswan* or brazil* or brunei* or bulgaria* or burkina faso* or burundi* or cambodia* or cameroon* or cape verde* or chad* or china* or Chinese or comoros* or congo* or cook islands* or cote d'ivoire* or croatia* or cuba* or cyprus* or Cypriot* or djibouti* or dominica* or ecuador* or el salvador* or egypt* or eritrea* or eswatini* or ethiopia* or micronesia* or fiji* or gabon* or gambia* or georgia* or ghana* or grenada* or guatemala* or guinea* or guyana* or haiti* or honduras* or india* or indonesia* or iran* or iraq* or jamaica* or jordan* or kazakhstan* or kenya* or kiribati* or kosovo* or kuwait* or kyrgyzstan* or laos* or lebanon* or liechtenstein* or lesotho* or liberia* or libya* or madagascar* or malawi* or malaysia* or maldives* or mali* or malta* or mauritania* or mauritius* or melanesia* or moldova* or monaco* or mongolia* or montenegro * or morocc* or mozambique* or myanmar* or namibia* or nauru* or nepal* or nicaragua* or niger* or niue* or oman* or pakistan* or palau* or palestin* or panama* or paraguay* or peru* or philippin* or polynesia* or qatar* or macedonia* or romania* or russia* or rwanda* or sahel* or "saint kitts and nevis" or "saint lucia" or "saint vincent and the grenadines*" or saudi arabia* or senegal* or serbia* or seychelles* or sierra leone* or singapore* or "sao tome and principe*" or solomon islands* or somalia* or south* asia* or sri lanka* or sudan* or suriname* or syria* or taiwan* or tajikistan* or tanzania* or thail* or timor* or togo* or tonga* or "trinidad and tobago" or tunisia* or turkmenistan* or tuvalu* or uganda* or ukrain* or "united arab emirates" or uruguay* or uzbekistan* or vanuatu* or venezuela* or vietnam* or sahara* or yemen* or zambia* or zimbabwe*).ti. 23212

37 (australia* or austria* or baltic* or belgium* or canad* or chile* or colombia* or costa rica* or czech* or denmark* or danish or estonia* or europe* or finland* or france* or French or german* or greece* or hungar* or iceland* or ireland* or irish or israel* or italy* or Italian* or japan* or korea* or latvia* or lithuania* or luxembourg* or mexic* or netherlands* or new zealand* or north america* or norway* or norweigan* or polish or poland* or portug* or scandinavia* or swede* or Swedish or slovak* or slovenia* or spain* or switzerland* or swiss or English or welsh or Scottish or england or wales or scotland or Britain or British or united kingdom* or turkey* or Turkish or united states* or western europe* or european union).ti. 20123

38 36 not 37 [Adapted NICE filter 2021] 22793

39 35 not 38 1699

**Ovid MEDLINE(R) ALL <1946 to October 03, 2023>**

**Date searched: 04/10/2023**

**Records identified: 2941**

1 Midwifery/ 21361

2 (midwif* or midwiv*).tw. 29066

3 Nurse Midwives/ 7546

4 (maternity adj3 worker*).tw. 117

5 (maternity adj3 staff*).tw. 323

6 (maternity adj3 assistant*).tw. 39

7 (msw* not ("municipal solid" or "male sex work*" or "medical social work*")).tw. 1775

8 or/1-7 [Maternity staff] 42434

9 (staff* adj3 (level* or ratio* or resourc* or model* or number* or mix* or rota* or rosta* or roster* or schedul* or overtime or supervision or supervisory or sufficient* or sufficiency or adequate* or adequac* or target* or insufficient* or insufficienc* or inadequate* or inadequac* or short or shortage* or efficient* or efficienc* or inefficien* or burnout or stress or fatigue or magnet)).tw. 21962

10 (skill?mix* or "skill? mix*").tw. 1282

11 (staffmix* or "staff mix*").tw. 112

12 staffing.tw. 17876

13 understaff*.tw. 827

14 "under staff*".tw. 89

15 "personnel staffing and scheduling"/ or shift work schedule/ or work-life balance/ 20098

16 Health Workforce/ 14511

17 manpower.tw,fs. 7919

18 (workload* or workforce* or shift or shiftwork* or shifts or overtime or capacity).tw. 1088736

19 Workload/ 24026

20 ("missed care" or "missing care").tw. 336

21 "care left undone".tw. 55

22 (hours adj2 day).tw. 9289

23 (work* adj2 hours).tw. 13570

24 (hours adj2 care).tw. 1755

25 (caseload or "case load*").tw. 4994

26 (turnover or "turn over").tw. 110824

27 (FTE or "full-time equivalent").tw. 1687

28 Burnout, Professional/ 17228

29 Occupational Stress/ 3577

30 Work Schedule Tolerance/ 7551

31 or/9-30 1297260

32 8 and 31 3659

33 (midwi* adj3 assistant* adj3 (level* or ratio* or resourc* or model* or number* or mix* or rota* or rosta* or roster* or schedul* or overtime or supervision or supervisory or sufficient* or sufficiency or adequate* or adequac* or target* or insufficient* or insufficienc* or inadequate* or inadequac* or short or shortage* or efficient* or efficienc* or inefficien* or burnout or stress or fatigue or magnet)).tw. 5

34 (midwi* adj3 worker* adj3 (level* or ratio* or resourc* or model* or number* or mix* or rota* or rosta* or roster* or schedul* or overtime or supervision or supervisory or sufficient* or sufficiency or adequate* or adequac* or target* or insufficient* or insufficienc* or inadequate* or inadequac* or short or shortage* or efficient* or efficienc* or inefficien* or burnout or stress or fatigue or magnet)).tw. 5

35 (maternity adj3 assistant* adj3 (level* or ratio* or resourc* or model* or number* or mix* or rota* or rosta* or roster* or schedul* or overtime or supervision or supervisory or sufficient* or sufficiency or adequate* or adequac* or target* or insufficient* or insufficienc* or inadequate* or inadequac* or short or shortage* or efficient* or efficienc* or inefficien* or burnout or stress or fatigue or magnet)).tw. 0

36 (maternity adj3 worker* adj3 (level* or ratio* or resourc* or model* or number* or mix* or rota* or rosta* or roster* or schedul* or overtime or supervision or supervisory or sufficient* or sufficiency or adequate* or adequac* or target* or insufficient* or insufficienc* or inadequate* or inadequac* or short or shortage* or efficient* or efficienc* or inefficien* or burnout or stress or fatigue or magnet)).tw. 5

37 (midwi* adj3 (level* or ratio* or resourc* or model* or number* or mix* or rota* or rosta* or roster* or schedul* or overtime or supervision or supervisory or sufficient* or sufficiency or adequate* or adequac* or target* or insufficient* or insufficienc* or inadequate* or inadequac* or short or shortage* or efficient* or efficienc* or inefficien* or burnout or stress or fatigue or magnet)).tw. 1703

38 or/33-37 1710

39 "named midwi*".tw. 14

40 32 or 38 or 39 [Maternity staffing issues search 1] 4905

41 Perinatal Care/ec, og, st [Economics, Organization & Administration, Standards] 1581

42 Delivery Rooms/ec, og, st [Economics, Organization & Administration, Standards] 511

43 Birthing Centers/ec, og, st [Economics, Organization & Administration, Standards] 389

44 Midwifery/ec, og [Economics, Organization & Administration] 2244

45 Nurse Midwives/ec, og [Economics, Organization & Administration] 862

46 41 or 42 or 43 or 44 or 45 [Maternity service staffing] 5152

47 40 or 46 [All maternity staffing searches] 9525

48 exp animals/ not humans/ 5160002

49 47 not 48 [Animal studies removed] 9511

50 afghanistan/ or africa/ or africa, northern/ or africa, central/ or africa, eastern/ or "africa south of the sahara"/ or africa, southern/ or africa, western/ or albania/ or algeria/ or andorra/ or angola/ or "antigua and barbuda"/ or argentina/ or armenia/ or azerbaijan/ or bahamas/ or bahrain/ or bangladesh/ or barbados/ or belize/ or benin/ or bhutan/ or bolivia/ or borneo/ or "bosnia and herzegovina"/ or botswana/ or brazil/ or brunei/ or bulgaria/ or burkina faso/ or burundi/ or cabo verde/ or cambodia/ or cameroon/ or central african republic/ or chad/ or exp china/ or comoros/ or congo/ or cote d'ivoire/ or croatia/ or cuba/ or "democratic republic of the congo"/ or cyprus/ or djibouti/ or dominica/ or dominican republic/ or ecuador/ or egypt/ or el salvador/ or equatorial guinea/ or eritrea/ or eswatini/ or ethiopia/ or fiji/ or gabon/ or gambia/ or "georgia (republic)"/ or ghana/ or grenada/ or guatemala/ or guinea/ or guinea-bissau/ or guyana/ or haiti/ or honduras/ or independent state of samoa/ or exp india/ or indian ocean islands/ or indochina/ or indonesia/ or iran/ or iraq/ or jamaica/ or jordan/ or kazakhstan/ or kenya/ or kosovo/ or kuwait/ or kyrgyzstan/ or laos/ or lebanon/ or liechtenstein/ or lesotho/ or liberia/ or libya/ or madagascar/ or malaysia/ or malawi/ or mali/ or malta/ or mauritania/ or mauritius/ or mekong valley/ or melanesia/ or micronesia/ or monaco/ or mongolia/ or montenegro/ or morocco/ or mozambique/ or myanmar/ or namibia/ or nepal/ or nicaragua/ or niger/ or nigeria/ or oman/ or pakistan/ or palau/ or exp panama/ or papua new guinea/ or paraguay/ or peru/ or philippines/ or qatar/ or "republic of belarus"/ or "republic of north macedonia"/ or romania/ or exp russia/ or rwanda/ or "saint kitts and nevis"/ or saint lucia/ or "saint vincent and the grenadines"/ or "sao tome and principe"/ or saudi arabia/ or serbia/ or sierra leone/ or senegal/ or seychelles/ or singapore/ or somalia/ or south africa/ or south sudan/ or sri lanka/ or sudan/ or suriname/ or syria/ or taiwan/ or tajikistan/ or tanzania/ or thailand/ or timor-leste/ or togo/ or tonga/ or "trinidad and tobago"/ or tunisia/ or turkmenistan/ or uganda/ or ukraine/ or united arab emirates/ or uruguay/ or uzbekistan/ or vanuatu/ or venezuela/ or vietnam/ or west indies/ or yemen/ or zambia/ or zimbabwe/ 1305849

51 "Organisation for Economic Co-Operation and Development"/ 561

52 australasia/ or exp australia/ or austria/ or baltic states/ or belgium/ or exp canada/ or chile/ or colombia/ or costa rica/ or czech republic/ or exp denmark/ or estonia/ or europe/ or finland/ or exp france/ or exp germany/ or greece/ or hungary/ or iceland/ or ireland/ or israel/ or exp italy/ or exp japan/ or korea/ or latvia/ or lithuania/ or luxembourg/ or mexico/ or netherlands/ or new zealand/ or north america/ or exp norway/ or poland/ or portugal/ or exp "republic of korea"/ or "scandinavian and nordic countries"/ or slovakia/ or slovenia/ or spain/ or sweden/ or switzerland/ or turkey/ or exp united kingdom/ or exp united states/ 3507202

53 European Union/ 17769

54 Developed Countries/ 21410

55 or/51-54 3523249

56 50 not 55 [OECD search filter NICE 2021] 1215974

57 49 not 56 [non-OECD countries removed] 7907

58 limit 57 to (comment or editorial or letter or news) 836

59 57 not 58 [ephemera removed] 7071

60 limit 59 to english language 6701

61 limit 60 to yr="2014 -Current" 2941

**CEA Registry https://cevr.tuftsmedicalcenter.org/databases/cea-registry**

**Date searched: 05/10/2023**

**Records identified: 8**

Basic interface

Searched for the following words individually: maternity; midwife; midwifery; midwives; MSW; MSWs in ‘methods’, ‘ratios’ and ‘utilities’

Download 2014+

**Websites and Search Engines search strategies**

**King’s Fund** [**http://www.kingsfund.org.uk/**](http://www.kingsfund.org.uk/)

**Search dates: 03/10/2023, 23/02/2022**

**Records identified: 70 found, 2 downloaded**

Searched ‘publications’ section for single words: midwife; midwifery; midwives; maternity, and published from 2014 onwards. Note – searched for each term separately

**Royal College of Midwives** [**https://www.rcm.org.uk/publications/**](https://www.rcm.org.uk/publications/)

**Search dates: 03/10/2023, 23/02/2022**

**Records identified: 19 found, 6 downloaded**

Searched ‘publications’ section for single words: workforce, staff, staffing, ratio

Limited to reports published 2014+

**Royal College of Paediatrics and Child Health** [**https://www.rcpch.ac.uk/**](https://www.rcpch.ac.uk/)

**Search dates: 03/10/2023, 23/02/2022**

**Records identified: 80 found, 12 downloaded**

Searched for phrases: midwife staff, midwife staffing, midwife workforce, midwifery staff, midwifery staffing, midwifery workforce, maternity staff, maternity staffing, maternity workforce. Note – searched for each phrase separately.

Limited to reports published 2014+

**Department of Health https://www.gov.uk/**

**Search dates: 03/10/2023, 23/02/2022**

**Records identified: 250 found, 3 downloaded**

Search 1

searched for the string: maternity midwives midwife midwifery. Note terms are automatically combined with OR boolean logic.

Results limited to About ‘Health and social care’ and About ‘National Health Service’

Results limited to In ‘Guidance and regulation’ or In ‘Policy papers and consultations’

Results limited to Updated ‘after 1 January 2014’

Search 2

searched for the string: maternity midwives midwife midwifery. Note terms are automatically combined with OR boolean logic.

Results limited to About ‘Health and social care’ and About ‘National Health Service’

Results limited to In ‘Research & Statistics’

Results limited to Updated ‘after 1 January 2014’

**NHS England http://www.england.nhs.uk/**

**Search dates: 03/10/2023, 23/02/2022**

**Records identified: 7 found, 2 downloaded**

Selected Publications Tab

Searched for Keyword: maternity midwifery midwife midwives. Note terms are automatically combined with OR boolean logic.

Results limit to Topic: Safe Staffing, or Topic: Workforce

**NHS Scotland https://www.publications.scot.nhs.uk/**

**Search dates: 03/10/2023, 23/02/2022**

**Records identified: 2**

Searched for keyword phrases: midwife staff, midwife staffing, midwife workforce, midwives staff, midwives staffing, midwives workforce, midwifery staff, midwifery staffing, midwifery workforce, maternity staff, maternity staffing, maternity workforce. Note – searched for each phrase separately.

Limited to reports published 2014+

**Welsh Government Statistics and Research** <https://gov.wales/statistics-and-research>

**Search dates: 03/10/2023, 23/02/2022**

**Records identified: 1**

Searched for single words: midwife; midwives; maternity staff; maternity staffing, and published from 2014 onwards. Note – searched for each term separately

Limited to Topics: Health & Social Care

Limited to Type: Research

**Scottish Government** [**https://www.gov.scot/publications/**](https://www.gov.scot/publications/)

**Search dates: 03/10/2023, 23/02/2022**

**Records identified: 0**

Searched: (maternity OR midwi*) AND staffing

Limited to Topics: Health & Social Care

Limited to Type: Research and Analysis

Limited to From: 01/01/2014 to 22/02/2022

**NICE Evidence** [**https://www.evidence.nhs.uk/**](https://www.evidence.nhs.uk/)

**Date searched: 22/02/2022 (Not available to search in 2023)**

**Records identified: 823**

Searched: (maternity OR midwi*) AND (staffing OR workforce OR shortage* OR sufficien* OR number*)

Limited to From 01/01/2014- 22/02/2022

Limited to Evidence type: Primary Research OR Systematic Reviews OR Health Technology Assessments OR Economic Evaluations

**Google Scholar** [**http://scholar.google.co.uk/**](http://scholar.google.co.uk/) searched via Harzing’s Publish or Perish

**Search dates: 03/10/2023, 23/02/2022**

**Records identified: 94 (93 downloaded, 1 was removed as an obvious duplicate)**

Searched in Title only: maternity|midwi* AND staffing|workforce|shortage*|sufficien*|number*

Limited to From: 2014- 2022
