# Supplementary material for: The impact of midwife workload on delivery of care, and mother and baby outcomes in maternity settings in OECD countries: A systematic review
Source: PLoS One. 2025 Aug 25;20(8):e0329117. doi: 10.1371/journal.pone.0329117 (PMC12377604; doi:10.1371/journal.pone.0329117)
Supplement: S3 File — (DOCX) [file pone.0329117.s003.docx]

# Supporting Information S3. Excluded Studies Table

## Full Screening Reference List

| #N | Citation | Exclusion Reason |
| --- | --- | --- |
| 1 | Adeyemo OO, Morelli EE, Kennedy HP (2022). How to Foster Effective Midwife-Obstetrician Collaboration on Labor and Birth Units: Qualitative Analysis of Experiences of Clinicians in the United States. Journal of Midwifery and Women's Health. 67:552-60. | Study design |
| 2 | Anon. (2022) Stronger together. Midwives.25:18-24. | Study design |
| 3 | Callander, E., et al. (2021). "Using epidemiological and health economic measures to inform maternity staffing decisions: A guide." Women & Birth: Journal of the Australian College of Midwives 23: 23. | Study design |
| 4 | Care Quality, C. (2020). "Getting safer faster: key areas for improvements in maternity services." London: CQC. | Study design |
| 5 | Care Quality C. (2022). Hampshire Hospitals NHS Foundation Trust: Basingstoke and North Hampshire Hospital. London: CQC. 28(21). | Study design |
| 6 | Care Quality C. (2023). “Countess of Chester Hospital NHS Foundation Trust”. | Study design |
| 7 | Care Quality C. (2023). “Nottingham University Hospitals NHS Trust: Queen's Medical Centre”. | Study design |
| 8 | Care Quality C. (2023). “Nottingham University Hospitals NHS Trust: Nottingham City Hospital”. 2023 | Study design |
| 9 | House of Commons Select Committee (2021). "The safety of maternity services in England: fourth report of session 2021–22: report, together with formal minutes relating to the report." | Study design |
| 10 | Healthcare Safety Investigation Branch (HSIB) (2020). "Delays to intrapartum intervention once fetal compromise is suspected." Farnborough: HSIB. | Study design |
| 11 | Isrctn. (2022). “Does the midwife-led continuity of carer model improve birth outcomes and maternal mental health in vulnerable women?” https://trialsearchwhoint/Trial2aspx?TrialID=ISRCTN31836167. | Study design |
| 12 | McLardie-Hore F.E., et al. (2023). “Comparing the views of caseload midwives working with First Nations families in an all-risk, culturally responsive model with midwives working in standard caseload models, using a cross-sectional survey design”. Women & Birth: Journal of the Australian College of Midwives. 36(5):469-80. | Study design |
| 13 | Molina, R. L., et al. (2018). "Association Between Labor and Delivery Unit Census and Delays in Patient Management: Findings From a Computer Simulation Module." Obstetrics & Gynecology 131(3): 545-552. | Study design |
| 14 | National Institute for Health and Care Excellence (NICE) (2021). "Induction of labour." London: NICE. | Study design |
| 15 | Ockenden D. Findings, conclusions and essential actions from the Independent Review of Maternity Services at The Shrewsbury and Telford Hospital NHS Trust: our final report (Ockenden review of maternity services at Shrewsbury and Telford Hospital NHS Trust.)(Ockenden report). London: Stationery Office, 2022, 234p. 2022. | Study design |
| 16 | Ockenden D. Ockenden report - final. Findings, conclusions and essential actions from the Independent Review of Maternity Services at The Shrewsbury and Telford Hospital NHS Trust. 2023. | Study design |
| 17 | Rottenstreich, M., et al. (2021). "Midwife annual delivery workload and maternal and neonatal adverse outcomes, is there an association?" European Journal of Obstetrics, Gynecology, & Reproductive Biology 262: 147-154. | Study design |
| 18 | Royal College of Midwives (2015). "Position statement: Safe midwife staffing." London: Royal College of Midwives. | Study design |
| 19 | Royal College of Midwives (2015). Safe Midwife Staffing: position statement. | Study design |
| 20 | Royal College of Midwives (2018). "State of maternity services report 2018 - England." London: RCM. | Study design |
| 21 | Royal College of Midwives (2023). “England State of maternity services 2023”. London: RCM July. 2023;11. | Study design |
| 22 | Royal College of Midwives (2023). “Northern Ireland State of maternity services 2023”. | Study design |
| 23 | Sands (2022). Safe Staffing: The impact of staffing shortages in maternity and neonatal care.Report of the Baby Loss and Maternity All Party Parliamentary Groups. | Study design |
| 24 | Schmitt, N., et al. (2021). "Effects of the Covid-19 pandemic on maternity staff in 2020 - a scoping review." BMC Health Services Research 21(1): 1364. | Study design |
| 25 | Sentilhes, L., et al. (2020). "Staffing needs for unscheduled activity in obstetrics and gynecology." European Journal of Obstetrics, Gynecology, & Reproductive Biology 245: 19-25. | Study design |
| 26 | Simpson, K. R. (2015). "Predicting nurse staffing needs for a labor and birth unit in a large-volume perinatal service." JOGNN - Journal of Obstetric, Gynecologic, & Neonatal Nursing 44(2): 329-338. | Study design |
| 27 | Simpson, K. R. (2017). "Safe nurse staffing is more than numbers and ratios." MCN - American Journal of Maternal Child Nursing 42(5): 304. | Study design |
| 28 | Tabatabaee, S. S. and E. Daghighbin (2020). "Estimating of the required midwife in maternity ward in hospital using Workload Indicator of Staffing Need Method." | Study design |
| 29 | Turner, L., et al. (2021). "Midwifery and nurse staffing of inpatient maternity services - A systematic scoping review of associations with outcomes and quality of care." Midwifery 103: 103118. | Study design |
| 30 | Veach K. (2022) Making commitments reality. InScope. (24):44-5. | Study design |
| 31 | Allkins S. (2022) The Ockenden report: what are the next steps? British Journal of Midwifery.30(5):245-. | Publication type |
| 32 | Australian Nurses and Midwives Federation. (2015). "The missing links in patient care." Aust Nurs Midwifery J 23(3): 11. | Publication type |
| 33 | Callander E.J. et al. (2023). CASELOAD MIDWIFERY COST ANALYSIS USING RESULTS from the COSMOS RANDOMISED CONTROLLED TRIAL. Journal of paediatrics and child health.59:135. | Publication type |
| 34 | ChiCtr (2020). "Dvelopment and implementation of midwife-based care model for urban women with uncomplicated pregnancies: a randomized controlled trial." https://trialsearch.who.int/Trial2.aspx?TrialID=ChiCTR2000033459. | Publication type |
| 35 | Clark, R., et al. (2021). "Effects of Nurse Staffing and Nurse Education on Missed Care and Breastfeeding Support in Maternity Units with Different Nurse Work Environments." 2021 Annual Research | Publication type |
| 36 | Collins A. (2023). “Trusts given five years to achieve safe midwife staffing”. Health Service Journal; 31. | Publication type |
| 37 | Dahlen H. et al. (2023). “O35 - The impact of the model of maternity care on perinatal outcomes during the COVID-19 Pandemic: The Birth in the Time of COVID-19 (BITTOC) Study”. Australian College of Midwives National Conference – Be the Change, September 12-14, 2023, Adelaide, South Australia. Women & Birth. 36:S14-S. | Publication type |
| 38 | Dube M.M., et al. (2023). “O31 - Effect of an Australian community-based caseload midwifery group practice service on maternal and neonatal outcomes for women from a refugee background”. Australian College of Midwives National Conference – Be the Change, September 12-14, 2023, Adelaide, South Australia. Women & Birth. 2023;36:S13-S. | Publication type |
| 39 | Ford, S. (2018). "Figures suggest almost half of maternity units forced to temporarily shut during 2017." Nursing Times 114(8): 149-149. | Publication type |
| 40 | Hales, K. (2015). "Kirkup Investigation into Maternity Care at Morecambe Bay Trust." Midwifery Matters(145): 3-3. | Publication type |
| 41 | Harby J. & Sissons R. (2023). “Derby maternity deaths might have been prevented – report”. BBC News. 22. | Publication type |
| 42 | House of Commons (2019). "Midwives [written answer]." Hansard. | Publication type |
| 43 | House of Commons (2021). "Midwives [written answer]." Hansard. | Publication type |
| 44 | House of Commons (2021). "Midwives and Obstetrics [written answer]." Hansard. | Publication type |
| 45 | House of Commons (2022). "Midwives in the NHS [debate]." Hansard 707. | Publication type |
| 46 | InScope. We Counted the BABIES 123. InScope. 2023(26):8-. | Publication type |
| 47 | Lai Thom A. (2022). “The impact of midwifery staffing levels on breastfeeding rates in a North West National Health Service (NHS) maternity service: a research proposal”. MIDIRS Midwifery Digest. 32(3):392-6. | Publication type |
| 48 | The Lamp (2023). Crucial moment for public hospitals. Lamp.80(2):10-3. | Publication type |
| 49 | Lamp Editorial Team (2022). Ratios vital for safety and job satisfaction. Lamp.79(4):17-. | Publication type |
| 50 | Merrifield, N. (2017). "Half of women report 'red flag' care delays 'due to midwife shortages', finds survey." Nursing Times 113(1): 1-2. | Publication type |
| 51 | Merrifield N. (2017) Scotland consulting on safe staffing law. Nursing Times.113(5):9-. | Publication type |
| 52 | Midwives (2017). BLURRED LINES. Midwives.20(4):12-3. | Publication type |
| 53 | Midwives (2023). Making a connection. Midwives. (26):23-8. | Publication type |
| 54 | Molloy, E. J., et al. (2018). "Developing core outcome set for women's, newborn, and child health: the CROWN Initiative." Pediatric Research 84(3): N.PAG-N.PAG. | Publication type |
| 55 | National Assembly for Wales (2023). “Midwifery Staffing: Rural and Remote Areas [written answer]”. Record of Proceedings Written question WQ87645. 3. | Publication type |
| 56 | Nursing Standard (2014). "Staffing is key issue according to NICE guideline." Nurs Stand 29(8): 8. | Publication type |
| 57 | Nursing Standard (2015). "NICE outlines warning signs of understaffing in maternity." Nurs Stand 29(27): 11. | Publication type |
| 58 | Royal College of Midwives (2022). Royal College of Midwives calls for NHS cultural shift to improve safety. British Journal of Healthcare Assistants.16(3):154-. | Publication type |
| 59 | Simpson K.R. (2022). “Innovative Strategies to Promote Safe Nurse Staffing in the Maternity, Neonatal, and Pediatric Acute Care Setting”. MCN: The American Journal of Maternal Child Nursing.47(5):241-. | Publication type |
| 60 | Singh A.K. et al. (2023). “The impact of certified nurse midwife presence on cesarean delivery rates during night shifts”. American Journal of Obstetrics & Gynecology. 228:S593-S4. | Publication type |
| 61 | Turner L. et al. (2022). “Are poor experiences on postnatal wards linked to staffing levels?”. Nursing Times. 2022;118(10):49-50. | Publication type |
| 62 | World of Irish Nursing & Midwifery. (2020). "Deliveries continue despite unsafe midwifery levels." World of Irish Nursing & Midwifery 28(5): 8-8. | Publication type |
| 63 | Aiken, C. E., et al. (2016). "The influence of hours worked prior to delivery on maternal and neonatal outcomes: a retrospective cohort study." American Journal of Obstetrics & Gynecology 215(5): 634.e631-634.e637. | No midwifery staffing variable |
| 64 | All Party Parliamentary Group (2023). All Party Parliamentary Group on M. Safe Staffing: The impact of staffing shortages in maternity and neonatal care. Report of the Baby Loss and Maternity All Party Parliamentary Groups. | No midwifery staffing variable |
| 65 | Bernstein SL et al. (2022). Systems-Level Factors Affecting Registered Nurses During Care of Women in Labor Experiencing Clinical Deterioration. Joint Commission Journal on Quality & Patient Safety.48(6):309-18. | No midwifery staffing variable |
| 66 | Blackman, I., et al. (2015). "Factors influencing why nursing care is missed." Journal of Clinical Nursing 24(1): 47-56. | No midwifery staffing variable |
| 67 | Cegolon L., et al. (2019). “Length of stay following vaginal deliveries: A population based study in the Friuli Venezia Giulia region (North-Eastern Italy), 2005-2015”. PloS one. 14(1):e0204919. | No midwifery staffing variable |
| 68 | Creswell L. et al. (2023). “A retrospective observational study of labour ward work Intensity: The challenge of maternity staffing. European Journal of Obstetrics, Gynecology, & Reproductive Biology”. 286:90-4. | No midwifery staffing variable |
| 69 | Edqvist M. et al. (2022). “The effect of two midwives during the second stage of labour to reduce severe perineal trauma (Oneplus): a multicentre, randomised controlled trial in Sweden”. Lancet (london, england). 399(10331):1242‐53. | No midwifery staffing variable |
| 70 | Greene, N. H., et al. (2020). "Impact of labor and delivery unit policy modifications on maternal and neonatal outcomes during the coronavirus disease 2019 pandemic." American Journal of Obstetrics & Gynecology MFM 2(4): 100234. | No midwifery staffing variable |
| 71 | Grigg, C. P., et al. (2017). "Evaluating Maternity Units: a prospective cohort study of freestanding midwife-led primary maternity units in New Zealand-clinical outcomes." BMJ Open 7(8): e016288. | No midwifery staffing variable |
| 72 | Harmsen van der Vliet-Torij H.W. et al. (2023). “Type of deliveries supported by Dutch clinical midwives”. Midwifery. 124:103744. | No midwifery staffing variable |
| 73 | Irish Nurses and Midwifery Organisation. 89% of nurses/midwives on brink of burnout -- INMO survey reveals. World of Irish Nursing & Midwifery. 2023;31(5):11-. | No midwifery staffing variable |
| 74 | Israel G (2023). “Integrating Community Health Workers and Nurse Midwives on the Health-Care Team to Improve Birth and Breastfeeding Outcomes”. Journal of Perinatal Education. 32(1):8-13. | No midwifery staffing variable |
| 75 | Jarrett O., et al. (2022). “Factors contributing to neonatal readmissions to a level 4 hospital within 28 days after birth. Journal of paediatrics and child health”. 58(7):1251-5. | No midwifery staffing variable |
| 76 | Jiang W., et al. (2023). “The Impact of the Workload and Traumatic Stress on the Presenteeism of Midwives: The Mediating Effect of Psychological Detachment”. Journal of Nursing Management. 1-10. | No midwifery staffing variable |
| 77 | Jolles D.R., et al. (2023). The birth center model of care: Staffing, business characteristics, and core clinical outcomes. Birth. 13:13. | No midwifery staffing variable |
| 78 | Lundsberg, L. S., et al. (2017). "Variation in Hospital Intrapartum Practices and Association With Cesarean Rate." JOGNN - Journal of Obstetric, Gynecologic, & Neonatal Nursing 46(1): 5-17. | No midwifery staffing variable |
| 79 | Martin, C. R., et al. (2017). "The Birth Satisfaction Scale-Revised Indicator (BSS-RI)." BMC Pregnancy & Childbirth 17(1): 277. | No midwifery staffing variable |
| 80 | Martin, P., et al. (2018). "Timing of singleton births by onset of labour and mode of birth in NHS maternity units in England, 2005-2014: A study of linked birth registration, birth notification, and hospital episode data." PLoS ONE [Electronic Resource] 13(6): e0198183. | No midwifery staffing variable |
| 81 | McLachlan H.L., et al. (2022). “Translating evidence into practice: Implementing culturally safe continuity of midwifery care for First Nations women in three maternity services in Victoria, Australia”. EClinicalMedicine. 47:101415. | No midwifery staffing variable |
| 82 | Mills T.A., et al. (2022). “Better maternity care pathways in pregnancies after stillbirth or neonatal death: a feasibility study”. BMC Pregnancy & Childbirth. 2022;22(1):634. | No midwifery staffing variable |
| 83 | Morcos, C. and A. B. Caughey (2018). "Passive descent in the second stage: evaluation of variation in practice patterns." Journal of Maternal-Fetal & Neonatal Medicine 31(17): 2271-2275. | No midwifery staffing variable |
| 84 | Neal S., et al. (2023). “Assessing safe and personalised maternity and neonatal care through a pandemic: a case study of outcomes and experiences in two trusts in England using the ASPIRE COVID-19 framework”. BMC health services research. 23(1):675. | No midwifery staffing variable |
| 85 | Nijagal, M. A., et al. (2018). "Standardized outcome measures for pregnancy and childbirth, an ICHOM proposal." BMC Health Services Research 18(1): 953. | No midwifery staffing variable |
| 86 | Nursing. CLINICAL ROUNDS. Midwifery services help reduce cesarean rates. Nursing. 2015;45(12):29-. | No midwifery staffing variable |
| 87 | Ostenfeld A., et al. (2023). “Reorganising and improving quality of care for hyperemesis gravidarum in a Danish hospital: a quality improvement project”. BMJ Open Quality. 12(3). | No midwifery staffing variable |
| 88 | Pangerl S., et al. (2022). “Adherence to screening and management guidelines of maternal Group B Streptococcus colonization in pregnancy”. Journal of Advanced Nursing. 78(10):3247-60. | No midwifery staffing variable |
| 89 | Poskiene I., et al. (2023). “Outcomes of low-risk birth care during the Covid-19 pandemic: A cohort study from a tertiary care center in Lithuania”. Open Medicine. 18(1):20230720. | No midwifery staffing variable |
| 90 | Pyykonen, A., et al. (2014). "Determining obstetric patient safety indicators: the differences in neonatal outcome measures between different-sized delivery units." BJOG: An International Journal of Obstetrics & Gynaecology 121(4): 430-437. | No midwifery staffing variable |
| 91 | Reif, P., et al. (2018). "Do time of birth, unit volume, and staff seniority affect neonatal outcome in deliveries at >=34+0 weeks of gestation?" BJOG: An International Journal of Obstetrics & Gynaecology 125(7): 884-891. | No midwifery staffing variable |
| 92 | Saturno-Hernandez, P. J., et al. (2019). "Indicators for monitoring maternal and neonatal quality care: a systematic review." BMC Pregnancy & Childbirth 19(1): 25. | No midwifery staffing variable |
| 93 | Sfregola, G., et al. (2017). "Work load and management in the delivery room: changing the direction of healthcare policy." Journal of Obstetrics & Gynaecology 37(2): 185-190. | No midwifery staffing variable |
| 94 | Thiessen K. et al. (2020) “Maternity Service Delivery in Manitoba, Canada: A Retrospective Analysis of Three Maternity Care Provider Types”. Canadian Journal of Midwifery Research & Practice. 19(1):6-19. | No midwifery staffing variable |
| 95 | Wallace J. et al. (2023). “An alternative model of maternity care for low-risk birth: Maternal and neonatal outcomes utilizing the midwifery-based birth center model”. Health Services Research. 10:10. | No midwifery staffing variable |
| 96 | Abdel-Hafez, A., et al. (2021). "The Clinical Nursing and Midwifery Dashboard (CNMD): A State-Wide Implementation." Studies in Health Technology & Informatics 284: 20-24. | Wrong outcome |
| 97 | Blackman, I., et al. (2018). "Modeling Missed Care: Implications for Evidence-Based Practice." Worldviews on Evidence-Based Nursing 15(3): 178-188. | Wrong outcome |
| 98 | Bowers, J. and H. Cheyne (2016). "Reducing the length of postnatal hospital stay: implications for cost and quality of care." BMC Health Services Research 16: 16. | Wrong outcome |
| 99 | Clark R.R.S., et al. (2022). “Nursing Resources by Type of Maternity Unit Across Regions of the United States”. JOGNN: Journal of Obstetric, Gynecologic & Neonatal Nursing. 51(3):290-301. | Wrong outcome |
| 100 | Grollman C. et al. (2022). “Maternity service reconfigurations for intrapartum and postnatal midwifery staffing shortages: modelling of low-risk births in England”. BMJ Open. 12(9):e051747. | Wrong outcome |
| 101 | Helm, C. (2017). "Safe and effective staffing: nursing against the odds." | Wrong outcome |
| 102 | Oriji, V. K., et al. (2018). "Peak Delivery Time in a Semi-Urban Primary Health Centre; Implication for Maternity Staffing." | Wrong outcome |
| 103 | Plotkin, L. (2017). "Support overdue: women's experiences of maternity services 2017." London: The National Federation of Women's Institutes (NFWI); NCT. | Wrong outcome |
| 104 | Siddiqui, I., et al. (2014). "Developing Objective Metrics for Unit Staffing (DOMUS) study." BMJ Open 4(9): e005398. | Wrong outcome |
| 105 | Simms, R. A., et al. (2014). "Using data and quality monitoring to enhance maternity outcomes: a qualitative study of risk managers' perspectives." BMJ Quality & Safety 23(6): 457-464. | Wrong outcome |
| 106 | Walsh, D., et al. (2018). "Mapping midwifery and obstetric units in England." Midwifery 56: 9-16. | Wrong outcome |
| 107 | Cookson, G., et al. (2014). "The Cost-Effectiveness of Midwifery Staffing and Skill Mix on Maternity Outcomes." A Report for the National Institute for Health and Care Excellence.” | In original NICE guideline/review |
| 108 | National Institute for Health and Care Excellence (2015). "Safe midwifery staffing for maternity settings. NICE Guideline [NG4]" London: NICE. | In original NICE guideline/review |
| 109 | Rowe, R. E., et al. (2014). "Service configuration, unit characteristics and variation in intervention rates in a national sample of obstetric units in England: an exploratory analysis." BMJ Open 4(5): e005551. | In original NICE guideline/review |
| 110 | Sandall, J., et al. (2014). "The efficient use of the maternity workforce and the implications for safety and quality in maternity care: a population-based, cross-sectional study." NIHR Journals Library. Health Services and Delivery Research 10: 10. | In original NICE guideline/review |
| 111 | Amiri, A. (2020). "Role of nurses and midwives in improving patient safety during childbirth: Evidence from obstetric trauma in OECD countries." Applied Nursing Research 56: N.PAG-N.PAG. | Wrong setting |
| 112 | Pfniss I., et al. (2023). “Birth during off‐hours: Impact of time of birth, staff´s seniority, and unit volume on maternal adverse outcomes—a population‐based cross‐sectional study of 87 065 deliveries. Birth: Issues in Perinatal Care”. 50(2):449-60. | Wrong setting |
| 113 | Snyder, J. E., et al. (2020). "Regional Variations in Maternal Mortality and Health Workforce Availability in the United States." Annals of Internal Medicine 173(11): S45-S54. | Wrong setting |
| 114 | Stones W. & Nair A. (2023). “Metrics for maternity unit staffing in low resource settings: Scoping review and proposed core indicator”. Frontiers in Global Womens Health. 4:1028273. | Wrong setting |
| 115 | Sentilhes, L., et al. (2019). "Human Resources for Unplanned Activities in Obstetrics and Gynecology. Consensus statements by the CNGOF, CARO, CNSF, FFRSP, SFAR, SFMP and SFN." Gynecologie Obstetrique Fertilite et Senologie 47(1): 63-78. | Foreign language |
