# Supplementary material for: The impact of midwife workload on delivery of care, and mother and baby outcomes in maternity settings in OECD countries: A systematic review
Source: PLoS One. 2025 Aug 25;20(8):e0329117. doi: 10.1371/journal.pone.0329117 (PMC12377604; doi:10.1371/journal.pone.0329117)
Supplement: S4 File — (DOCX) [file pone.0329117.s004.docx]

# Supporting Information S4 Data Extraction & Risk of Bias Assessments RQ1

Data Extraction Tables

| **Author** | **Dani et al. (2019)** |
| --- | --- |
| Study characteristics | *Location:* Careggi University Hospital of Florence, Italy includes:   - “Margherita” centre, an in hospital midwife led centre with 300 births per year - An obstetrician-led centre, with 3100 births per year   *Study Design:* Cohort study with recruitment between January and June 2018, comparison made between outcomes for obstetrician-led centre and midwife led centre which have different midwife to infant ratios. Total of 220 recruited, 110 from each centre.  *Population includes:*   - Healthy infants with gestational age of >= 37 weeks and healthy birth weight - Birth after *uncomplicated* pregnancy   *Population excludes:*   - Any event that could interfere with post-natal course e.g. need for resuscitation   *Analytical methods*   - Multiple logistic regression |
| Staffing resource | The staffing resource is not directly measured. The study reports the following midwife to infant ratios for each centre:   - ‘Margherita’ centre 1:2.5 from 7.01am to 1.00pm, 1:5 from 1.01pm to 7.00am; - Obstetrician-led centre: 1:7 from 7.01am to 1.00pm, 1:9 from 1.01pm to 8.00pm and 1:15 from 8.01pm to 7.00am   Comparison is made between the Margherita centre and the obstetrician-led centre, interpretation assumes that the difference in midwife ratios between the centres is the cause of differences in outcomes. No description is provided on how midwife to infant ratios are obtained. |
| Outcome measures | Primary outcome:   - Exclusive breastfeeding rate   Secondary outcomes:   - Admission to neonatal ward - Length of hospital stay   No maternal, birth or process outcomes are used. It is stated that these outcomes are balanced between groups due to selection process e.g. sample only includes population with uncomplicated pregnancy. |
| Covariates | Table 1 includes descriptive statistics by centre for mother’s age; gestational age, birth weight, length, head circumference, Apgar score, Cord pH, peak weight loss %, weight at discharge, change of weight at discharge, sodium > 145 mEqL, peak TSP. It’s not clear if these are also used as covariates in the regression. No staffing variables are reported. |
| Risk of bias | The study is rated as “poor” quality on NHLBI quality assessment tool. |
| Findings | Midwife led centres (vs obstetrician led centres) had:   - Higher rates of exclusive breastfeeding (88% vs 78%) p=0.048 - Lower admission rate to neonatal care units (2% vs 9%) p=0.017 - Decreased length of hospital stay in days (2.6 +/- 0.8 vs. 3.1 +/- 1.8, p = 0.008)   Logistic regression results for midwife led vs obstetrician led, reported as odds ratios (OR) with the associated 95% confidence intervals in parenthesis.   - Exclusive breastfeeding OR 2.04 (1.07-3.92) - Neonatal care OR 0.17 (0.07-0.43) - Hospital stay OR 0.81 (0.51 – 1.23) |
| Interpretation (authors) | More favourable midwife to infant ratio can explain better outcomes in midwife led centre, given maternal and neonatal assistance and breastfeeding promotion followed same strategies, and staff training is consistent across centres. |
| Notes | The primary outcome is not directly relevant as it is post-natal. The study does not directly examine the associations between staffing ratios and outcomes, these are stated as being different between units, but no information is provided on how midwife to infant ratios are calculated. Women self-select into OU or midwife led units, it’s not clear if all relevant covariates are adjusted for and therefore differences between outcomes may be due to other differences in units and not due to staff ratios. |
| Data extractor | Richard Mattock |
| Date of extraction | 09 September 2022 |
| Eligible for RQ1 | Yes |

| **Author** | **Facchini (2020)** |
| --- | --- |
| Study characteristics | Population includes 6,142 births from mothers with spontaneous onset of labour (excludes planned caesarean sections and planned inductions). Population from maternity unit in a large teaching hospital in Tuscany (Italy) during 2011 to 2014. Links census data with hospital administrative data.  Premise is that during high midwife workload: (i) midwives may provide lower quality care, which might eventually result in the need for a caesarean section and (ii) caesarean sections might be increased through physician induced demand where it may be optimal to shift patients to physicians by changing the delivery method (caesarean sections are quicker than vaginal birth). |
| Study design | Study uses plausibly exogenous variability in the number of patients and midwives present at admission to causally identify the impact of workload on delivery method. Time of arrival for delivery in unknown to hospitals and capacity utilization is unknown to patients.  Analysis 1: OLS regression (linear probability model) where outcome is binary indicator for Caesarean section (yes/no) and explanatory variable is the ratio of patients to midwives (RPM).  Analysis 2: Same regression as analysis 1 but with an interaction between RPM and whether mothers are married (yes/no). This is to test patient induced demand under the assumption that non married mothers are more likely to experience patient induced demand. |
| Staffing resource | Ratio of patients to midwives in the delivery room *at the time of admission*.  The number of patients does not include those who are scheduled to give by Caesarean section (reason is that these cases would increase the number of physicians ready to perform Caesarean sections).  The number of midwives is the number of staff scheduled to be present in the delivery room each month, day of the week and shift combination. This is not staff present at each point in time but staffing rules of the delivery room.  RPM is measured as a linear variable (continuous) and as a dummy variable for high RPM if patient is admitted when the RPM is above the 20^th^ percentile. |
| Outcome measures | Primary outcome is caesarean-section (unscheduled).  Secondary outcomes: Process (operative vaginal birth, episiotomy); maternal morbidity (post partum haemorrhage, length of stay; infant morbidity (achieved skin to skin contact with mother, exclusive breastfeeding rate, APGAR <9, transfer to neonatal intensive care unit). |
| Covariates | Covariates include individual level variables of the mother and pregnancy characteristics, and fixed effects for shift, day of the week, month and year of admission. |
| Risk of bias | Ranked as good quality. |
| Findings | **Analysis 1:**  *Delivery outcomes*  RPM as a linear variable (continuous) is not statistically significant. The coefficient for RPM is 0.007.  RPM as non-linear variable is statistically significant (p<0.05). The regression coefficient for high RPM is 0.021. Caesarean section rate for patients admitted during low RPM is 8.9%, and 10.8% when admitted with high RPM, i.e. a 19% increase in unscheduled caesarean section.  There is no evidence of a relationship between RPM and the secondary outcomes operative vaginal birth and episiotomy, or administration of analgesia.  *Maternal and infant outcomes*  The coefficient for RPM on post-partum haemorrhage is 0.041 (p<0.01), there is a 21% rise in the probability of having post-partum haemorrhage for high versus low RPM. This is driven almost entirely by an increase in Caesarean sections, the coefficient for RPM reduced to 0.009 (p>0.10) when including caesarean section in the regression equations.  RPM has no impact on length of stay.  RPM has no impact on neonatal outcomes including skin-to-skin contact, exclusive breastfeeding, Apgar score, and admission to the neonatal unit.  **Analysis 2:**  Single mothers admitted at high workload levels have caesarean section rate 4.5% higher than those during low workload, this effect is statistically significant. The interaction between workload and marital status is statistically significant. Married women’s delivery method is unaffected by workload. |
| Interpretation | High workload leads to an increased likelihood of delivering by Caesarean section. There is no evidence to suggest that increased rate of Caesarean section is due to decreased care and subsequently increased clinical need for Caesarean section. Worse maternal outcomes appear to be driven by the increase in Caesarean section rates for higher workloads. There is suggestive evidence that physician induced demand explains the increase in Caesarean section rates: single and married women have the same probability of Caesarean section when workload is low, likelihood increases with workload only for single women. It is suggested that single women are more likely to be alone in the delivery room and are easier to induce (in relation to agency discrimination model not labour induction).  There is no evidence of impact on length of stay and neonatal health outcomes, however, these were secondary outcomes and estimates were imprecise due to small sample sizes and the rarity of some of the conditions. |
| Data extractor | Richard Mattock |
| Date of extraction | 09 September 2022 |
| Eligible for RQ1 | Yes |

| **Author** | **Freeman (2016)** |
| --- | --- |
| Study characteristics | Delivery unit in the maternity department of a large UK teaching hospital. Includes 16,355 births across 5 years between April 2008 and March 2013.  The population includes   - Unscheduled births   The population excludes   - Scheduled patients (i.e. elective Caesarean sections) - Transfers from midwife-led birthing units - Very high risk patients (gestation <34 weeks, birth weight <2000g, deliveries resulting in stillbirths - All excluded patients are *included* in the estimation of the workload measure |
| Study design | Econometric model, informed through the “gatekeeper-provider” model, where midwives are assumed to act as gatekeepers and providers (GP) of care. The study evaluates the impact of increased service demand on midwives, also accounting for the complexity of cases.  Cases are defined as non-complex if labour started spontaneously, and complex if labour was pharmacologically induced in hospital prior to arrival on the delivery unit.  The study hypotheses are that when facing increased demand midwives will:   - Decrease discretionary care (i.e. epidurals), which will be more pronounced for non-complex cases - Increase referrals to specialists, which will be greater for complex cases   The main analysis is a Probit model, including independent variables for midwife workload, and dependent variables for (1) number of epidurals (to indicate discretionary care) and (2) number of specialist referrals.  Secondary outcomes of perineal tear rate, and Apgar score analysed using a Probit model, length of stay is analysed using an OLS model and Heckman Selection model. |
| Staffing resource | Real time data on number of midwives and births in the delivery unit at any time during a patient episode.  The variable included in the model is workload, which is a standardized time weighted average number of patients per midwife for the period three hours before birth. |
| Outcome measures | Primary outcomes for delivery of care:   - Rate of epidural - Referral rate to specialists   Secondary outcomes related to maternal and baby health are:   - Incidence of third and fourth degree perineal tears - Apgar score 5-minutes after delivery - Length of time patient spent in the delivery unit |
| Covariates | Case complexity, dummy variable equal to 1 if there is a need for pharmacological induction (38% are complex)  Maternal characteristics: Age, BMI, number of previous births, age at first birth, previous caesarean-section  Pregnancy characteristics: Gestation, baby weight  Temporal: Daily trend, year quarter, hour of birth, weekend birth  Clinical complications: breech, malpresentation, shoulder dystocia, obstructed labor, diabetes, hypertension, PROM, COPD, other complications  Contextual factors: Deprivation index, health index, distance to hospital, antenatal stay, number of antenatal visits  Other operational factors: proportion epidural, proportion physician led, proportion escalated, post birth workload |
| Risk of bias | Study ranked as “good” quality on the NHLBI quality assessment tool. |
| Findings | Results reported as average partial effects (APE) for a continuous midwife workload  Higher workload associated with decreased epidurals: APE -0.025 (p<0.001)  Higher workload not associated with physician led delivery: APE -0.002, p not significant.  Higher workload leads to an increase in perineal tear rate for complex cases (APE = 0.011, p=0.001), but not for non-complex cases (APE =0.001, p=0.715).  Authors report no evidence of an association between workload and Apgar score.  For non-complex cases, an increase in workload results in a decrease in post-birth length of stay (APE = -0.018, p<0.01). Moving from low to high workload conditions indirectly (through reduction in epidural rate) causes an 8.3% decrease in post birth length of stay. For complex cases no association is identified on length of stay (APE = 0.007, p >0.10) |
| Interpretation (authors) | Findings suggest that midwives make substantial use of two levers to manage their workload (measured as patients per midwife): they ration resource-intensive discretionary services (epidural analgesia) for customers with noncomplex service needs (mothers with spontaneous onset of labor) and, increase the rate of specialist referral (physician-led delivery) for customers with complex needs (mothers with pharmacologically induced labor).  The results are consistent with over treatment at both high and low workload levels, but for different types of patients.  It is not possible to estimate the effect of midwife rationing and referral decisions on tear rate or Apgar score, due to lack of precision in a BiProbit model. Results are for a basic Probit model which cannot specifically comment on how workload induced changes in midwife behaviour affect this outcome. |
| Data extractor | Richard Mattock |
| Date of extraction | 15 September 2022 |
| Eligible for RQ1 | Yes |

| **Author** | **Hollowell (2015)** |
| --- | --- |
| Study characteristics | Further analyses from the Birthplace in England prospective cohort study.  Setting is obstetric units, alongside midwifery units, freestanding midwifery units and planned home birth in England.  Study 3 is relevant to RQ1 – it explores whether unit trust characteristics (e.g. staffing) are associated in variations in delivery/ maternal and neonatal outcomes.  Birthplace cohort study was a prospective cohort study with planned place of birth at start of care in labour as exposure. Groups include home birth, birth in functional midwifery unit, alongside midwifery unit, and obstetrics unit.  Included: women who were attended by NHS midwife during labour in planned place of birth for any amount of time  Excluded: planned caesarean section, preterm labour, multiple pregnancy, women who received no antenatal care, stillbirth.  Sample was split into “low risk” subgroup with term pregnancy (37 to 42 weeks gestation). This included 16,753 planning birth in an AMU, 11,210 in a FMU and 16,632 home births.  The data was combined with 19,686 staffing and workload logs, with 17,359 (88%) remained after cleaning. |
| Study design | Simple linear regression to examine associations between unit or trust characteristics and adjusted intervention, outcome and transfer rates.  Exploratory analyses conducted for several associations identified in the data, and assessed using Pearson’s correlation coefficient.  Sensitivity analyses were conducted by plotting data on scatter graphs to identify outliers and re-estimate equations with and without the outliers. |
| Staffing resource | Staffing and organisational data were collected alongside the Birthplace cohort study using daily staffing and workload logs which were completed at 09:00 and 21:00 by midwives in the obstetric and midwifery units. This included number of women in the unit, the number of midwives and maternity support workers on duty, and obstetric cover during the previous 12 hours.  Staffing variables were measured as the proportion of occasions where “understaffing” occurs in a unit, with understaffing defined when number of women in labour exceeds number of midwives on duty. Data is obtained on the wards on the day of delivery using the Birthplace staffing logs. It is then condensed to an average for the unit as a whole, over time to estimate % of days understaffed. |
| Outcome measures | Intervention during labour and birth   - instrumental delivery (ventouse or forceps) - Intrapartum caesarean section   Uncomplicated birth:   - “Straightforward birth” – without caesarean section, forceps or ventouse, third-/fourth-degree perineal trauma, or blood transfusion   Normal birth:   - birth without induction of labour, epidural or spinal analgesia, general anaesthetic, forceps or ventouse, caesarean section, or episiotomy   Maternal composite:   - one or more of augmentation, forceps or ventouse, intrapartum caesarean section, general anaesthetic, blood transfusion, third-/fourth-degree perineal trauma, and maternal admission to higher-level care   Transfer:   - Intrapartum transfer during labour or immediately after birth - Duration of transfer   Perinatal outcomes:   - Neonatal admission - Composite primary outcome from Birthplace study |
| Covariates | Study explores factors related to the mother: parity, age, ethnicity, index of multiple deprivation, completed weeks of gestation, BMI, marital status, understanding of English, complicating conditions (yes/ no).  The regression analysis is reported as “simple linear regression” suggesting that it does not include covariates. |
| Risk of bias | Study ranked as poor quality with high risk of bias due to type of staffing variable and lack of adjustment for potential confounders. |
| Findings | Intervention rates: No clear association between understaffing and intervention rates in OUs or AMUs. Some statistically significant findings but these occur in the opposite direction than may be expected e.g. higher rates of “straightforward birth” with more understaffing, and are inconsistent across types of unit.  Transfer rates: In AMUs, although higher midwifery staffing was significantly associated with higher transfer rates in multiparous women, there was no consistent relationship between any of the unit characteristics studied and transfer rates. There was no association with transfers rates and understaffing in FMUs.  Full results below reported separately by ward type. Significant associations are highlighted in **bold** text.  Obstetric units:  Nulliparous:   - Instrumental delivery: 0.02 (p= 0.798) - **Intrapartum caesarean section: -0.10 (p= 0.025)** - Straightforward birth: 0.06 (p= 0.307) - Normal birth: -0.01 (p= 0.889) - Epidural 0.05 (0.594) - Augmentation -0.10 (0.156)   Multiparous:   - Instrumental delivery: -0.04 (p= 0.068) - Intrapartum caesarean section: -0.05 (p= 0.106) - **Straightforward birth: 0.08 (p= 0.011)** - Normal birth: 0.05 (p= 0.482) - Epidural 0.0 (0.942) - **Augmentation -0.09 (0.048)**   Results for AMUs:  Nulliparous:   - Instrumental delivery: 0.17 (p= 0.483) - Intrapartum caesarean section: 0.05 (p= 0.511) - Straightforward birth: -0.28 (p= 0.255) - Normal birth: -0.34 (p= 0.254) - Transfer: 0.2 (p = 0.415)   Multiparous:   - Instrumental delivery: 0.04 (p= 0.471) - Intrapartum caesarean section: 0.00 (p= 0.956) - Straightforward birth: -0.06 (p= 0.496) - Normal birth: 0.03 (p= 0.735) - Transfer: -0.1 (p =0.569)   Results for FMUs:  Nulliparous:   - Instrumental delivery: -0.17 (p= 0.455) - Intrapartum caesarean section: 0.05 (p= 0.670) - Straightforward birth: 0.11 (p= 0.685) - Normal birth: -0.33 (p= 0.222)   Multiparous:   - Instrumental delivery: -0.01 (p= 0.847) - Intrapartum caesarean section: -0.05 (p= 0.046) - Straightforward birth: 0.07 (p= 0.462) - Normal birth: 0.13 (p= 0.119) |
| Interpretation | Understaffing was associated with significant variation in rates of some interventions in planned obstetric unit births in low risk women, but the lack of a consistent significant effect across multiple outcomes means reasonable possibility that these were chance findings. |
| Notes | Main purpose of the study is to guide women where to give birth rather than to investigate staffing. The staffing variable is collected on the ward at time of delivery but is then condensed to an average over time i.e. the % of time that the unit is understaffed from 0% to 100%. Therefore, variable does not identify specific births where maternity unit is understaffed, just whether the unit is understaffed in general. |
| Data extractor | Richard Mattock |
| Date of extraction | 15 September 2022 |
| Eligible for RQ1 | Yes |

| **Author** | **Isidore (2018)** |
| --- | --- |
| Study characteristics | 204 French midwives recruited from November 2015 to May 2016. A survey was administered online, midwives were invited to participant through invitations in the Paris public hospital system and social networks e.g. a French midwives Facebook groups. |
| Study Design | Descriptive cross-sectional study. Midwives completed an online survey including a case vignette including hourly partograms of a slowly progressing labour, and a short self-administered questionnaire.  The vignette described the spontaneous labour at 40 weeks of gestation of a 29-year old first time mother progressing slowly during the active phase of labour, with epidural, analgesia, after spontaneous rupture of membranes. Uterine contractility, fetal heart rate and cervical dilation are described hour by hour. Two choices are proposed with each hourly partogram: administration of oxytocin or expectant management. If oxytocin was selected, responses were obtained on starting dose, augmentation dose, and minimum time before increase.  A separate questionnaire asked midwives about their individual characteristic (e.g. time in work), organisational factors (e.g. number of delivery rooms, number of annual births) and factors they thought might influence use of oxytocin daily.  Two tailed statistical tests, with p<0.05, were conducted using Chi-squared or Fishers exact tests (categorical data) or Student’s t-test or Wilcoxon’s test for continuous data. |
| Staffing resource | Measures are derived from questionnaire responses and not measured directly on wards.  Midwives workload = the number of annual births in the maternity unit / number of midwives working daily.  Delivery room overload = the number of annual birth in maternity unit / number of delivery rooms |
| Outcome measures | Primary outcome: oxytocin administration and dose. Note this is not an observed outcome from practice, but midwives stated care when presented with a case vignette. |
| Covariates | None. The selection of statistical tests don’t allow for the inclusion of covariates. |
| Risk of bias | Study rated as “poor” quality on NIH quality assessment tool. |
| Findings | Observed widespread use of oxytocin (77.9% of mid-wives), high doses (64.1%>2mI U/min) and short dose-increase delays (62.9% <30min).  Midwives workload was higher for groups who prescribed oxytocin use (mean = 987.5, SD = 241.7) versus those that did not (mean = 937.6, SD = 984.1) (p<0.001).  Delivery room overload was higher for groups who prescribed oxytocin use (mean = 489.5, SD = 135.6) versus those that did not (mean = 472.2, SD = 130.3) (p<0.001). |
| Interpretation | Administration of oxytocin by midwives was significantly associated with higher annual number of deliveries, higher midwife workloads, and with overloaded delivery rooms. According to current guidelines, oxytocin administration is not indicated for the clinical vignette, therefore midwife responses are inappropriate in 77.9% of cases. |
| Notes | The analysis does not include any covariates. The study shows that multiple factors are associated with increased oxytocin usage e.g. level of neonatal care unit, protocol in delivery room, maternity workload, overloaded deliver rooms. These factors are likely to be correlated so could be confounded. Also strong potential for confounding with unobserved variables. |
| Data extractor | Richard Mattock |
| Date of extraction | 12 December 2022 |
| Eligible for RQ1 | Yes |

| **Author** | **Kpea (2015)** |
| --- | --- |
| Study characteristics | Cross sectional data from 2010 French National Perinatal Survey, collected through interviews with mothers in post partum wards, from medical records, and on maternity unit organization through questionnaires completed by the head of each unit.  Sample included 7,123 women with vaginal deliveries who were low risk for caesarean delivery, 22 weeks or more gestation, neonatal weight >=500g.  Excluded fetal deaths, caesareans, induced labour, medical indications for neuraxial analgesia.  The sample was split into those with: 5,288 (74.2%) women with preference for labour with neuraxial analgesia; and 1,835 (25.8%) with preference without neuraxial analgesia which included 961 (52.4%) who received analgesia during pregnancy and 874 (47.6%) who did not |
| Study design | First analysis conducted for all women using univariate and multivariate analyses to test associations between preference for labour without neuraxial analgesia.  Second analysis conducted to estimate the use of neuraxial analgesia in the sub sample of women who initially preferred not to.  Univariate associations were tested with chi squared tests, multivariate analyses were conducted using Poisson regression models with robust error variance. Random effects were included for maternity unit based on hierarchical data. Interaction terms were tested between covariates. Missing data was excluded from the analysis. |
| Staffing resource | Midwives workload, estimated at each unit by calculating the ratio of number of midwives per shift in the labour ward to the number of annual deliveries. Workload was considered high in the quartile with the lowest ratio (i.e. the 25% of maternity units with the fewest midwives per annual deliveries). |
| Outcome measures | Neuraxial analgesia (epidural pain relief). |
| Covariates | Maternal: adverse obstetrical history, chronic medical conditions, medical disorders. Characteristics of labour: gestational age, oxytocin augmentation of labour, mode of vaginal delivery. Characteristics of maternity unit: public/private, availability of anaesthesiologist. |
| Risk of bias | Study ranked as fair quality with high risk of bias. |
| Findings | High midwife workload was significantly associated with increased use of neuraxial analgesia in population of women who did not originally want it, adjusted relative risk = 1.1, p = 0.0289. |
| Interpretation | High midwife workload was associated with a greater rate of neuraxial analgesia in women who did not initially want it. It is known that continuous support during labor influences the management and outcome of labor and reduces the likelihood that this analgesia will be administered. It is possible that midwives encourage the placement of neuraxial analgesia when their workload is high if they think that women with neuraxial analgesia require less support than those without it. |
| Notes | Midwife staffing levels is not the primary focus. Midwife staffing ratios to birth are measured annually and therefore don’t account for random peaks in demand that occur daily/weekly. The covariates don’t include other staff rates on the ward which may confound the relationship between workload and outcomes. |
| Data extractor | Richard Mattock |
| Date of extraction | 15 September 2022 |
| Eligible for RQ1 | Yes |

| **Author** | **Draper (2017)** |
| --- | --- |
| Study characteristics | The third perinatal confidential enquiry carried out by MBRRACE-UK into stillbirths and perinatal deaths. The enquiry aimed to identify potentially preventable failures of care during labour, delivery and any resuscitation which might have contributed to perinatal death. The study considered 225 out of 4392 (5.1%) perinatal deaths that occurred in the UK in 2015, 104 of which were randomly selected for the enquiry. Deaths eligible for inclusion were >= 37 weeks at gestational age, singleton, intrapartum stillbirths, and intrapartum-related neonatal deaths in 2015. |
| Study design | A random sample of 104 eligible cases (perinatal deaths) were identified and selected for enquiry. The enquiry was a process of systematic, multidisciplinary, anonymous case reviews where a consensus opinion is reached about the quality-of-care provision for all cases undergoing review. Ten separate panel meetings were conducted to discuss cases. The panel group consisted of multidisciplinary team included obstetricians, midwives, neonatologists, neonatal nurses, anaesthetists, and perinatal pathologists. Panel members received anonymised notes of each case prior to meetings. Meetings discussed each case in turn, following a checklist of activities. Panel groups classified cases as having “good care, no improvements identified”, “improvements in care identified which would have made no difference to outcome”, and “improvements in care which may have made a difference to the outcome”. The research did not include any counterfactuals e.g. cases that did not result in neonatal death. |
| Staffing resource | No specific definition used. Panel described this as “staffing and capacity issues” |
| Outcome measures | Neonatal death, still birth, induction of labour |
| Covariates | None |
| Risk of bias | Study ranked as poor with a high risk of bias. |
| Findings | The panel consensus was that in nearly 80% of deaths improvements in care were identified which may have made a difference to the outcome of the baby.  Capacity issues were identified as a problem in over a quarter of the cases undergoing panel review (n=21). The majority of staffing and capacity problems were related to delivery suite (n=17) with the remaining issues relating to neonatal care provision.  In four cases problems of unit staff capacity were indicated which may have prevented women being admitted to the most appropriate setting.  In ten cases the mother was delayed in transferring from either an antenatal setting or a midwifery-led unit to the delivery suite, due to either the lack of a room, increased activity levels, or a shortage of staff.  In four cases induction labour was delayed for up to two days due to unit capacity issues and in one case a woman experienced a five-hour delay in performing an artificial rupture of the membranes because of increased activity of the unit. |
| Interpretation | During periods of high activity the ability of the wider maternity service to cope with the demand for one-to-one care and/or timely review by obstetric or medical staff is sometimes compromised |
| Data extractor | Richard Mattock |
| Date of extraction | 15 September 2022 |
| Eligible for RQ1 | Yes |
| **Author** | **Turner (2022)** |
| Study characteristics | A brief report looking at how the experiences of women on post-natal wards in the UK are linked to staffing levels. |
| Study design | Cross sectional secondary data analysis linking responses from a 2018 maternity survey to staffing data for midwives. Individual patient data obtained from the UK Data service. Includes data from 129 trusts and 17,611 individuals. Multilevel logistic regression models were fitted accounting for different trusts. |
| Staffing resource | The number of FTE midwives was extracted from NHS Digital’s (2018) workforce statistics. Staffing was measured as a continuous variable, then by dividing the variable into tertiles. |
| Outcome measures | Survey of maternal responses to post-natal survey. Of four questions asked, one was relevant for inclusion: Did mothers experience a delay in discharge? (Yes, No) |
| Covariates | Parity, type of birth, age, ethnicity, size of trust. |
| Risk of bias | The study is ranked as “poor” quality with high risk of bias. |
| Findings | Women cared for in trusts with higher levels of midwifery staffing had more favourable responses to each of the questions on postnatal care. Regression models found every additional FTE midwife per 100 births in the organisation is associated with 15% reduction in delays in discharge, odds ratio 0.85, 95% CI 0.75- 0.96. An estimated 5.7% fewer women (1 in 18) reported a delay in discharge in trusts in the highest tertile for staffing (3.7-5.2 FTE midwives per 100 births) compared with the lowest tertile (2.5-3.4 FTE midwives per 100 births) |
| Interpretation | The study found improved maternal experiences in trusts employing a higher number of midwives. A causal link cannot be attributed, because results come from a cross-sectional study. The staffing levels in this study were not measured at ward level, therefore, differences seen at organisational level may not translate into proportional staffing on postnatal wards. |
| Data extractor | Richard Mattock |
| Date of extraction | 11 July 2024 |
| Eligible for RQ1 | Yes |

| **Author** | **Vanderlaan (2023)** |
| --- | --- |
| Study characteristics | Study from the USA exploring the impact of practice type (independent midwifery practice, and restricted midwifery practice) on pregnancy outcomes (caesarean section and preterm birth). The study explores how midwifery workforce density (i.e. workload) may moderate the association between practice type and pregnancy outcomes. |
| Study design | Cross sectional design. Data on practice type and birth records are obtained for 6 states from the State Inpatient Databases. This is merged with county level data on midwife density. The study included 875,156 women. Logistic regression analysis is used, with caesarean section/ pre term birth as the dependent variable and practice type and midwifery workforce density as independent variables. An interaction term is added between practice type and midwifery workforce density to explore moderating effects. |
| Staffing resource | Midwife density is the number of midwives per 1000 births per year in the county, classified as no midwives, low midwife density (<4.5 per 1000 births), and high midwife density (>=4.5 midwives per 1000 births). The variable is calculated from data in the Area Health Resource File based on the number of advanced practice midwives with National Provider Identifier numbers.  Classifications were based on symmetric distributions i.e. 10% of counties had zero midwives, and 10% of counties have >= 4.5 midwives per 1000 births. |
| Outcome measures | Caesarean section measured using the Inpatient Quality Indicator 22: Primary Caesarean Uncomplicated from the Agency for Healthcare Research and Quality. Preterm birth measured as any birth less than 37 completed weeks’ gestation based on the ICD-10-CM codes for gestational age (Z3A). |
| Covariates | Maternal race (Asian or Pacific Islander, Black, Hispanic, Native American, White, or other); primary payer source (Medicaid or other), maternal risk scored sing the Obstetric Comorbidity Index (OCI); access to obstetricians (calculated as number of obstetricians per 1000 births per year in country); access to maternity hospitals (ordinal, derivation not stated); state midwifery Medicaid parity (yes/no); maternal state of residence. |
| Risk of bias | The study is ranked as “fair” quality with moderate risk of bias. |
| Findings | Prevalence of caesarean section was the same in counties with no midwives and high midwife density (15.2%) but higher in counties with low midwife density (19.29%; P < .001). Small but significant differences in prevalence of preterm birth, with lowest rates for women in counties with low midwife density and highest in counties with no midwives (10.5% vs. 11% p <0.001).  There was evidence of moderation effects on both outcomes. The interaction between restricted practice and no midwives was significant for caesarean section (p = 0.040) and preterm birth (p = 0.001). The interaction between restricted practice and counties with low midwife density was significant for caesarean section (P < .001), preterm birth (P = 0.11). The largest magnitude of difference was the increased odds of preterm birth in counties with a high midwife density and restricted practice (odds ratio, 3.50; 95% CI, 2.43-5.06) compared with those with high midwife density and independent practice. |
| Interpretation | Midwife density moderates the association between independent midwifery practice and primary caesarean birth and preterm birth. The magnitude of association between perinatal outcomes and restricted practice differs between counties with no midwives, low midwife density, and high midwife density. |
| Data extractor | Richard Mattock |
| Date of extraction | 11 July 2024 |
| Eligible for RQ1 | Yes |

| **Author** | **Zbiri (2018)** |
| --- | --- |
| Study characteristics | Retrospective cohort study identifying factors that influence caesarean delivery rates in France.  Study in perinatal network, covering 11 maternity units in district of Yvelines (west of Paris) from 2008 to 2014. Population included over 100,000 deliveries and over 24,000 caesarean deliveries.  Perinatal and maternal morbidity and mortality outcomes, demographic characteristics, and pregnancy and delivery outcomes obtained from CoNaissance 78 database. Data is recorded at two points on certificates completed by midwives and physicians.  Data on maternity units obtained from Annual Statistics for French Hospitals database.  Three analyses were conducted:   1. Prelabour urgent caesarean – this population included all elective, intrapartum caesarean and vaginal deliveries 2. Planned elective caesarean delivery – this population included planned elective caesarean and planned vaginal deliveries (intrapartum caesarean and vaginal deliveries) 3. Intrapartum caesarean deliveries – population included all vaginal deliveries |
| Study design | Multilevel logistic regression model, with women nested within maternity units. Estimated hospital specific random intercepts and robust variance for dependence between observations. Conducted bivariate and multivariate analyses with results reported as odds ratios. |
| Staffing resource | The variable included in models was the number of FTEs per 100 deliveries.  Number of FTE obstetricians, anaesthesiologists, and midwives was an average annual FTE. Assumption that part time private physicians were 50% FTE.  Number of unit (number of annual deliveries >1000, 1000 to 1999, or >=2000). |
| Outcome measures | Mode of delivery. Three analyses were conducted for three different outcomes:  Prelabour urgent caesarean (yes/no); Planned elective caesarean delivery (yes/no); intrapartum caesarean delivery (yes/no). |
| Covariates | Demographic characteristics: maternal age, parity;  Medical characteristics: previous caesarean, medical risk, plurality, preterm delivery, fetal presentation, induced labour, and birth weight;  Maternity unit information: type of organization (public/private); university status; level of neonatal care  Maternity unit organizational factors: day of delivery (working day or weekend); obstetrician availability; |
| Risk of bias | The study has been categorised as “poor” with a high risk of bias. |
| Findings | Bivariate analysis results for staff related variables:   \|  \| OR [95% CI] \| \| \| \| --- \| --- \| --- \| --- \| \|  \| Urgent caesarean \| Elective caesarean \| Intrapartum caesarean \| \| Obstetricians FTEs/100 births \| 1.11 (0.62-1.99) \| 0.83 (0.62-1.10) \| 0.54 (0.40 – 0.71) \| \| Anaesthesiologists FTEs/100 births \| 1.42 (0.64-3.18) \| 1.07 (0.69-1.66) \| 1.17 (0.74 – 1.84) \| \| Midwives FTEs/100 births \| 1.16 (0.81-1.66) \| 0.84 (0.64-1.09) \| 1.06 (0.80 – 1.41) \|   Multivariate analysis results (extracted outcomes for staff only not other demographic/birth/hospital outcomes that were included as covaraties)   \|  \| OR [95% CI] \| \| \| \| --- \| --- \| --- \| --- \| \|  \| Urgent caesarean \| Elective caesarean \| Intrapartum caesarean \| \| Obstetricians FTEs/100 births \| 1.26 (0.58-2.74) \| 0.91 (0.57 – 1.45) \| 0.55 (0.36 – 0.83) \| \| Anaesthesiologists FTEs/100 births \| 1.33 (0.71- 2.48) \| 0.99 (0.70 – 1.40) \| 1.14 (0.72 – 1.82) \| \| Midwives FTEs/100 births \| 1.40 (0.76-2.60) \| 0.79 (0.69 – 0.90) \| 1.11 (0.84 – 1.48) \|   From elasticity study:  Probability of intrapartum caesarean delivery with respect to the number of FTE obstetricians per 100 deliveries was -0.25 (95% CI -0.43,-0.08, P-value = 0.004)  Probability of elective caesarean delivery with respect to the number of FTE midwives per 100 deliveries was -0.34 (95% CI -0.52,-0.15, P-value < 0.001)  Likelihood of intrapartum caesarean delivery associated with a decrease of 2.5 percentage points per 10% increase in obstetrician level. Likelihood of elective caesarean delivery associated with decrease of 3.4 percentage points per 10% increase in midwife level. |
| Interpretation | Observed a statistically significant association between staffing levels of the maternity unit and caesarean delivery use.  As the number of FTE obstetricians per 100 deliveries per unit increased, the rate of intrapartum caesarean deliveries decreased. This may be due to improved care, e.g. through the availability of a full time laborist on units, improved organization and co-ordination with teams, and lessened time pressures.  As the number of FTE midwives per 100 deliveries increases, the rate of elective caesarean deliveries decreased. Midwives may affect women’s preferences about labour and delivery, reduce anxiety, and reassure them to deliver normally. |
| Notes | For intrapartum caesarean there is a significant increase if obstetricians per 100 deliveries is reduced, but a non-significant decrease if midwives or anaesthesiologists per 100 deliveries is reduced. Interpretation could be that these outcomes are linked, e.g. an increase in obstetricians necessarily means a decrease in other staff members. |
| Data extractor | Richard Mattock |
| Date of extraction | 18 September 2022 |
| Eligible for RQ1 | Yes |
| **Author** | **Robertson (2021)** |
| Study characteristics | Two analyses were conducted. The first part was a retrospective analysis of real-world data that is relevant for RQ1. The second part is a simulation study of intervention effectiveness which is not relevant.  The setting is a maternity unit in a large UK district general hospital (Stoke Mandeville Hospital, Buckinghamshire NHS Trust). 4,932 women were included who gave birth in 2018.  The labour ward comprises 10 delivery rooms and 4 observation beds for high-risk patients, as well as a three-bedded Triage unit for women attending acutely.  The retrospective analysis used anonymised electronic hospital records. |
| Study design | Simple univariate linear regression between delay in induction of labour and causative factors. |
| Staffing resource | Staffing “shortfall” per 24-hour period and staffing levels were assessed using the Birthrate Plus (BR+) system. This allows for calculation of the number of midwives required on a labour ward, accounting for both demand and case mix. When compared with actual staffing, BR+ allows calculation of staffing shortfall as the number of midwives short for the case mix over a given time period. |
| Outcome measures | Delay to the induction of labour (IOL). IOL was defined as any labour started artificially by any means in the absence of spontaneous labour, and was defined as booked or emergency depending on indication. The outcome was number of IOL delayed by more than 12 hours within one week. |
| Covariates | None – the model was a simple univariate linear regression. |
| Risk of bias | Study ranked as poor quality with a high risk of bias due to lack of adjustment for any potential confounders. |
| Findings | Total number of women (p = 0.008) and total number of IOLs (p =0.041) and number of booked inductions (p = 0.009) were significantly associated with delay in IOLs. There was no significant association between staffing shortfall and delay in IOLs. |
| Interpretation | Staffing shortfall is not associated with delays to induction of labour. |
| Notes | The staffing variable is less prone to measurement error as it is collected on the ward at the time of delivery and is adjusted for case mix. |
| Data extractor | Richard Mattock |
| Date of extraction | 20 March 2025 |
| Eligible for RQ1 | Yes |

| **Author** | **Lyndon (2022)** |
| --- | --- |
| Study characteristics | Observational study establishing the relationship between nurse-reported staffing, missed nursing care during labour and birth, and exclusive breast milk feeding during childbirth.  The study used nurse survey data emailed to 10,620 nurses from 277 labour and birth units in 37 states across the United States from Feb 2018 to July 2019. In total 3,676 responses were provided.  Hospital data were obtained from American Hospital Association Annual Survey and the 2018 Joint Commission Health Care Quality data.  Inclusion criteria for nurses were working in labour and birth units that report >=40 births per year. Only hospitals with >= 4 surveys were included in hospital level analyses, or if response rates were greater than >35%. |
| Study design | All predictor, mediator and covariates were aggregated to hospital level. Bivariate linear regression was used to identify associations between nurse and hospital characteristics and outcome measures. |
| Staffing resource | Nurse perception of unit adherence to staffing guidelines on a 4-point Likert-type scale for each of 14 items presenting recommended nurse-to-patient ratios to particular parts of care. A staffing score was created by taking the mean of their individual responses scores, with higher scores indicating better adherence to staffing. |
| Outcome measures | Exclusive breast milk feeding rate during the birth hospitalisation. Perinatal missed care obtained from surveys of nurses using the MISSCARE survey which includes 25 items related to essential nursing care during labour and birth (e.g. delayed, unfinished or missed care) measured using a 4-point Likert type scale. The 25 items were dichotomized and summed to a final score. |
| Covariates | Patient safety climate measured from nurses’ responses using seven items from the Safety Climate subscale of the Safety Attitudes Questionnaire; nurse demographics including age, education, years of experience, years working at current hospital, shift work, gender, race, ethnicity; hospital characteristics including birth volume, obstetric care, critical access and sole community provider status, teaching status, rurality. |
| Risk of bias | Study ranked as poor quality with risk of bias as staffing was measured through surveys and represents an average across the study period i.e. not measured directly on wards during delivery. |
| Findings | No association between  Positive associations were identified between staffing and breast milk feeding (beta = 0.141, p<0.001)  Negative associations between staffing and missed skin-to-skin care (beta -0.098, p not reported)  Negative associations between staffing and missed breastfeeding care within one hour of birth (beta -0.107, p not reported) |
| Interpretation | Nurses’ responses to staff surveys on staffing were associated with breast milk feeding rates. |
| Notes | The perinatal missed care outcome was considered a mediator for this analysis but is a relevant outcome measure for the review. |
| Data extractor | Richard Mattock |
| Date of extraction | 20 March 2025 |
| Eligible for RQ1 | Yes |

| **Author** | **Wilson (2020)** |
| --- | --- |
| Study characteristics | Retrospective descriptive study examining nurse staffing patterns on the likelihood of caesarean section.  Data were obtained from 11-hospitals from a large integrated health care system in the United States for low-risk term gestation deliveries occurring between October 2016 and September 2017.  The sample was restricted to full gestation births (37 to 42 weeks) with no indicators of high clinical risk. Sample sizes reported in Tables are 2,419. |
| Study design | The study uses multiple linear regression specifications to analyse the impact of nursing hours (H) per delivery (D) on c-section outcomes. The first model uses a simple hours of nursing care divided by number of deliveries (H/D). Two other models are used to check whether the current level of staffing results in optimal c-section outcomes. The second model includes a quadratic term (H/D) + (H/D)^2^. The third model uses a piecewise regression (H/D) + (H/D – X)*Dum^i^ where X is the number of hours in a birthing protocol that minimises risk of c-section and Dum^i^ is a dummy variable based on H/D values greater than or equal to X. |
| Staffing resource | Staffing is measured as nursing hours (per delivery) with various specifications in the regression equations as described in the study design. No information is provided on how nursing hours are measured or how the number of deliveries is measured. |
| Outcome measures | Rate of c-section, however no details are provided on the data sources.  Secondary analyses are performed on rates of induction and augmentation. |
| Covariates | Weeks of gestation, race, sex of the child, mother’s age. |
| Risk of bias | The study was ranked as fair quality due to the level of reporting where it is not clear how and when the exposure and outcomes were measured. |
| Findings | They found no meaningful or significant relationship between the staffing term (H/D) and c-section rates (coefficient -0.0002). There was a positive coefficient (0.011) associated with (H/D)^2^ term (0.011, non-significant). When applying the “optimal” model with 31.2 hours staff time the coefficient for the (H/D)^2^ term reduces to near zero (0.00002).  Rates of augmentation are significantly associated with staffing (p <0.01): [H/D] coefficient = 0.001, [H/D]^2^ coefficient = 0.009  Rates of induction are significantly associated with staffing (p <0.01): [H/D] coefficient = 0.005, [H/D]^2^ coefficient = 0.050 |
| Interpretation | Simple linear regression of the likelihood of a C-section on nursing hours per delivery indicated no distinguishable effect. Two complimentary models (the quadratic and piecewise regressions) distinguishing optimal staffing patterns from ineffective staffing patterns suggested that current nurse staffing hours minimize c-sections. |
| Notes | A robustness check is performed on “yesterday’s nursing hours” so it might be assumed that nursing hours are measured at the time of deliveries. |
| Data extractor | Richard Mattock |
| Date of extraction | 21 March 2025 |
| Eligible for RQ1 | Yes |

| **Author** | **Mercer (2016)** |
| --- | --- |
| Study characteristics | Observational cohort study of 101,120 in 24 hospitals in the USA. |
| Study design | Multivariable regression. |
| Staffing resource | Nurse patient ratios (NPRs) were the total nursing hours per shift / births per shift/8 hours. Due to level of reporting it is not clear if these were measured on the wards at the time of deliver, or are an aggregate measure. |
| Outcome measures | Postpartum hemorrhage, 5-minute Apgar below 4 (LoAp5), Hypoxic Ischemic Encephalopathy (HIE), Shoulder dystocia (SD), and cord pH below 7.0. |
| Covariates | Not reported. |
| Risk of bias | The study was ranked as poor quality with high risk of bias due to the level of reporting (conference abstract). |
| Findings | There were no significant associations between frequencies of any adverse perinatal complications and nurse patient ratios. |
| Interpretation | Nurse patient ratios do not predict adverse perinatal outcomes. |
| Notes | The publication is a conference abstract with limited reporting detail. |
| Data extractor | Richard Mattock |
| Date of extraction | 21 March 2025 |
| Eligible for RQ1 | Yes |

| **Author** | **Knape (2014)** |
| --- | --- |
| Study characteristics | Study examines the influence of ‘supportive care’ by midwives on the mode of birth in a population of low-risk women in German multicentre sample. The study is conducted in 1,238 deliveries. |
| Study design | Prospective controlled multicentre trial. Backward logistic regression models were used to analyse associations between workload of midwives and mode of birth. |
| Staffing resource | Midwives registered their time with women and their workload during their shifts using self-constructed time documentation. The variable "workload" measured whether midwives were caring for more than one woman during their shift (or not). If midwives were required to care for more than one woman per shift, they were not able to guarantee one-to-one support for a woman. The measure didn’t specify whether cases happened at the same time or how long each case lasted. |
| Outcome measures | Mode of birth, all the spontaneous births defined one group, and all the operative deliveries and unplanned caesareans defined the second group. |
| Covariates | N/A |
| Risk of bias | The study was ranked as low quality for the review question. |
| Findings | Midwives’ workload was not associated with mode of birth in the multivariate model and was therefore not included in the final model.  In bivariate (unadjusted) models workload was significantly associated with mode of birth (increased risk of caesarean section or operative delivery 11% vs 20.1% (p <0.01). |
| Interpretation | The hypothesis that midwife workload influences mode of birth outcomes is not supported. |
| Notes | Due to backward logistic regression methods, midwife workload was removed from the multivariate analysis so no results are reported. |
| Data extractor | Richard Mattock |
| Date of extraction | 24 March 2025 |
| Eligible for RQ1 | Yes |

Risk of bias assessment

| **Dani et al. (2019)** | | | |  |
| --- | --- | --- | --- | --- |
| ***Major Components*** | ***Response*** | | | ***Notes*** |
| 1. Was the research question or objective in this paper clearly stated? | Yes |  |  |  |
| 2. Was the study population clearly specified and defined? | Yes |  |  |  |
| 3. Was the participation rate of eligible persons at least 50%? |  |  | Not Reported |  |
| 4. Were all the subjects selected or recruited from the same or similar populations (including the same time period)? Were inclusion and exclusion criteria for being in the study prespecified and applied uniformly to all participants? | Yes |  |  |  |
| 5. Was a sample size justification, power description, or variance and effect estimates provided? | Yes |  |  | Primary outcome only |
| 6. For the analyses in this paper, were the exposure(s) of interest measured prior to the outcome(s) being measured? | Yes |  |  |  |
| 7. Was the timeframe sufficient so that one could reasonably expect to see an association between exposure and outcome if it existed? | Yes |  |  |  |
| 8. For exposures that can vary in amount or level, did the study examine different levels of the exposure as related to the outcome (e.g., categories of exposure, or exposure measured as continuous variable)? |  | No |  |  |
| 9. Were the exposure measures (independent variables) clearly defined, valid, reliable, and implemented consistently across all study participants? |  | No |  | Exposure (midwife to birth ratio) was not measured directly. Study compared difference between OC and midwife led units, which are reported to have different staffing ratios so assumed that all differences in units caused by ratios. Not clear when ratios are measured or how. |
| 10. Was the exposure(s) assessed more than once over time? |  | No |  |  |
| 11. Were the outcome measures (dependent variables) clearly defined, valid, reliable, and implemented consistently across all study participants? | Yes |  |  |  |
| 12. Were the outcome assessors blinded to the exposure status of participants? |  |  | Not Reported |  |
| 13. Was loss to follow-up after baseline 20% or less? |  |  | Not Reported |  |
| 14. Were key potential confounding variables measured and adjusted statistically for their impact on the relationship between exposure(s) and outcome(s)? |  |  | Not Reported | List of variables reported in Table 1 but not clear if these are covariates or not. |
| Quality Rating |  |  | Poor |  |
| Additional Comments (If Poor, please state why):  Midwife to birth ratio is reported to be different between midwives led and obstetric led units. Differences between outcomes for units may be due to other factors not related to staffing ratios. It is not clear how or when staffing ratios are measured. | | | | |
| Data extractor Richard Mattock  Date of extraction 09 September 2022 | | | | |

| **Facchini (2020)** | | | | |
| --- | --- | --- | --- | --- |
| ***Major Components*** | ***Response options*** | | | ***Notes*** |
| 1. Was the research question or objective in this paper clearly stated? | Yes |  |  |  |
| 2. Was the study population clearly specified and defined? | Yes |  |  |  |
| 3. Was the participation rate of eligible persons at least 50%? | Yes |  |  |  |
| 4. Were all the subjects selected or recruited from the same or similar populations (including the same time period)? Were inclusion and exclusion criteria for being in the study prespecified and applied uniformly to all participants? | Yes |  |  |  |
| 5. Was a sample size justification, power description, or variance and effect estimates provided? |  | No |  | Large sample size, should have adequate power. |
| 6. For the analyses in this paper, were the exposure(s) of interest measured prior to the outcome(s) being measured? | Yes |  |  | Measured on admission |
| 7. Was the timeframe sufficient so that one could reasonably expect to see an association between exposure and outcome if it existed? | Yes |  |  |  |
| 8. For exposures that can vary in amount or level, did the study examine different levels of the exposure as related to the outcome (e.g., categories of exposure, or exposure measured as continuous variable)? | Yes |  |  | Both dummy variable for high and low workload, and continuous measured. |
| 9. Were the exposure measures (independent variables) clearly defined, valid, reliable, and implemented consistently across all study participants? |  | No |  | Staffing was not measured directly, but assumed to be equivalent to staffing rosters i.e. the number of staff that were expected to be on wards on the day of delivery. |
| 10. Was the exposure(s) assessed more than once over time? | Yes |  |  | Results for days prior to admission in sensitivity analysis. |
| 11. Were the outcome measures (dependent variables) clearly defined, valid, reliable, and implemented consistently across all study participants? | Yes |  |  |  |
| 12. Were the outcome assessors blinded to the exposure status of participants? |  |  | Not Reported |  |
| 13. Was loss to follow-up after baseline 20% or less? | Yes |  |  |  |
| 14. Were key potential confounding variables measured and adjusted statistically for their impact on the relationship between exposure(s) and outcome(s)? | Yes |  |  |  |
| Quality Rating | Good |  |  |  |
| Additional Comments (If Poor, please state why): | | | |  |
| Data extractor Richard Mattock  Date of extraction 09 September 2022 | | | |  |

| **Freeman et al. (2016)** | | | | |
| --- | --- | --- | --- | --- |
| ***Major Components*** | ***Response options*** | | | ***Notes*** |
| 1. Was the research question or objective in this paper clearly stated? | Yes |  |  |  |
| 2. Was the study population clearly specified and defined? | Yes |  |  |  |
| 3. Was the participation rate of eligible persons at least 50%? | Yes |  |  |  |
| 4. Were all the subjects selected or recruited from the same or similar populations (including the same time period)? Were inclusion and exclusion criteria for being in the study prespecified and applied uniformly to all participants? | Yes |  |  |  |
| 5. Was a sample size justification, power description, or variance and effect estimates provided? |  | No |  | Very large sample size so power not an issue. |
| 6. For the analyses in this paper, were the exposure(s) of interest measured prior to the outcome(s) being measured? | Yes |  |  |  |
| 7. Was the timeframe sufficient so that one could reasonably expect to see an association between exposure and outcome if it existed? | Yes |  |  |  |
| 8. For exposures that can vary in amount or level, did the study examine different levels of the exposure as related to the outcome (e.g., categories of exposure, or exposure measured as continuous variable)? | Yes |  |  | Midwife load measured as a continuous variable |
| 9. Were the exposure measures (independent variables) clearly defined, valid, reliable, and implemented consistently across all study participants? | Yes |  |  |  |
| 10. Was the exposure(s) assessed more than once over time? | Yes |  |  | Weighted average across three time points |
| 11. Were the outcome measures (dependent variables) clearly defined, valid, reliable, and implemented consistently across all study participants? | Yes |  |  | Secondary outcomes are relevant for RQ1. |
| 12. Were the outcome assessors blinded to the exposure status of participants? |  |  | Not Reported |  |
| 13. Was loss to follow-up after baseline 20% or less? |  |  | Not Reported |  |
| 14. Were key potential confounding variables measured and adjusted statistically for their impact on the relationship between exposure(s) and outcome(s)? | Yes |  |  | Yes, extensive list of covariates |
| Quality Rating | Good |  |  |  |
| Additional Comments (If Poor, please state why):  Only secondary outcomes are relevant for RQ1, however these are well reported in an Appendix. The analytical design may have been improved if these were primary outcomes as the study isn’t able to identify the direct impact of changes to midwife practices on maternal and baby outcomes in fact of increasing demand. | | | |  |
| Data extractor Richard Mattock  Date of extraction 09 September 2022 | | | |  |

| **Hollowell (2015)** | | | | |
| --- | --- | --- | --- | --- |
| ***Major Components*** | ***Response options*** | | | ***Notes*** |
| 1. Was the research question or objective in this paper clearly stated? | Yes |  |  |  |
| 2. Was the study population clearly specified and defined? | Yes |  |  |  |
| 3. Was the participation rate of eligible persons at least 50%? | Yes |  |  |  |
| 4. Were all the subjects selected or recruited from the same or similar populations (including the same time period)? Were inclusion and exclusion criteria for being in the study prespecified and applied uniformly to all participants? | Yes |  |  |  |
| 5. Was a sample size justification, power description, or variance and effect estimates provided? |  | No |  | Large sample size, should have adequate power. |
| 6. For the analyses in this paper, were the exposure(s) of interest measured prior to the outcome(s) being measured? |  |  | Don’t know | Staffing was measured on the ward through rosters at time of delivery but was aggregated to % understaffing over time. Aggregated measure may have included observations prior to delivery. |
| 7. Was the timeframe sufficient so that one could reasonably expect to see an association between exposure and outcome if it existed? | Yes |  |  |  |
| 8. For exposures that can vary in amount or level, did the study examine different levels of the exposure as related to the outcome (e.g., categories of exposure, or exposure measured as continuous variable)? | Yes |  |  | Staffing was measured as a continuous variable i.e. % understaffed. |
| 9. Were the exposure measures (independent variables) clearly defined, valid, reliable, and implemented consistently across all study participants? |  | No |  | The exposure status was based on aggregated % understaffed in a unit. It’s not clear over what period the values were aggregated. |
| 10. Was the exposure(s) assessed more than once over time? | Yes |  |  |  |
| 11. Were the outcome measures (dependent variables) clearly defined, valid, reliable, and implemented consistently across all study participants? | Yes |  |  |  |
| 12. Were the outcome assessors blinded to the exposure status of participants? |  |  | Not Reported |  |
| 13. Was loss to follow-up after baseline 20% or less? |  |  | Not Reported |  |
| 14. Were key potential confounding variables measured and adjusted statistically for their impact on the relationship between exposure(s) and outcome(s)? |  | No |  | Analysis was reported as “simple linear regression” and does not mention any covariates. |
| Quality Rating |  |  | Poor |  |
| Additional Comments (If Poor, please state why):  The study is ranked as poor in relation to interpretations regarding midwife staffing levels. The “understaffing” variable is an aggregate across multiple time points and therefore may not be applicable to births that occur when unit is sufficiently staffed. The analyses only use simple linear regression and do not appear to include any covaraites. | | | |  |
| Data extractor Richard Mattock  Date of extraction 09 September 2022 | | | |  |

| **Isodore et al. (2018)** | | | | |
| --- | --- | --- | --- | --- |
| ***Major Components*** | ***Response options*** | | | ***Notes*** |
| 1. Was the research question or objective in this paper clearly stated? | Yes |  |  |  |
| 2. Was the study population clearly specified and defined? | Yes |  |  |  |
| 3. Was the participation rate of eligible persons at least 50%? |  |  | Not Reported |  |
| 4. Were all the subjects selected or recruited from the same or similar populations (including the same time period)? Were inclusion and exclusion criteria for being in the study prespecified and applied uniformly to all participants? |  |  | Not Reported |  |
| 5. Was a sample size justification, power description, or variance and effect estimates provided? |  | No |  |  |
| 6. For the analyses in this paper, were the exposure(s) of interest measured prior to the outcome(s) being measured? | Yes |  |  | Assuming “exposure” is midwives workload stated prior to clinical vignette exercise. |
| 7. Was the timeframe sufficient so that one could reasonably expect to see an association between exposure and outcome if it existed? |  |  | Not applicable | The outcomes were hypothetical i.e. what midwives state that they would do for a case vignette. |
| 8. For exposures that can vary in amount or level, did the study examine different levels of the exposure as related to the outcome (e.g., categories of exposure, or exposure measured as continuous variable)? | Yes |  |  | Midwife workload is measured as a continuous variable |
| 9. Were the exposure measures (independent variables) clearly defined, valid, reliable, and implemented consistently across all study participants? |  | No |  | Workload includes annual births and does not account for daily variations in demand. |
| 10. Was the exposure(s) assessed more than once over time? |  | No |  |  |
| 11. Were the outcome measures (dependent variables) clearly defined, valid, reliable, and implemented consistently across all study participants? |  | No |  | Outcome measures are how nurses think they would respond to clinical vignette, not their actual responses in practice. |
| 12. Were the outcome assessors blinded to the exposure status of participants? |  |  | Not Reported |  |
| 13. Was loss to follow-up after baseline 20% or less? |  |  | Not Reported |  |
| 14. Were key potential confounding variables measured and adjusted statistically for their impact on the relationship between exposure(s) and outcome(s)? |  | No |  | The statistical analysis was simple statistical tests of association between two variables. No covariates were included in statistical analyses e.g. by using multivariate regression analysis. Several independent variables were associated with the outcome, but no analysis was done to assess correlations between the independent variables. |
| Quality Rating |  |  | Poor |  |
| Additional Comments (If Poor, please state why):  There is a very high risk of confounding bias as the statistical analysis does not include covariates. The outcome was not measured, rather implied based on what midwives would do in given scenarios. | | | |  |
| Data extractor Richard Mattock  Date of extraction 12 December 2022 | | | |  |

| **Kpea (2015)** | | | | |
| --- | --- | --- | --- | --- |
| ***Major Components*** | ***Response options*** | | | ***Notes*** |
| 1. Was the research question or objective in this paper clearly stated? | Yes |  |  |  |
| 2. Was the study population clearly specified and defined? | Yes |  |  |  |
| 3. Was the participation rate of eligible persons at least 50%? |  |  | Not Reported |  |
| 4. Were all the subjects selected or recruited from the same or similar populations (including the same time period)? Were inclusion and exclusion criteria for being in the study prespecified and applied uniformly to all participants? | Yes |  |  |  |
| 5. Was a sample size justification, power description, or variance and effect estimates provided? |  | No |  | Large sample size, should have adequate power. |
| 6. For the analyses in this paper, were the exposure(s) of interest measured prior to the outcome(s) being measured? | Yes |  |  |  |
| 7. Was the timeframe sufficient so that one could reasonably expect to see an association between exposure and outcome if it existed? | Yes |  |  |  |
| 8. For exposures that can vary in amount or level, did the study examine different levels of the exposure as related to the outcome (e.g., categories of exposure, or exposure measured as continuous variable)? |  | No |  | Single dummy variable for high midwife workload used. |
| 9. Were the exposure measures (independent variables) clearly defined, valid, reliable, and implemented consistently across all study participants? |  | No |  | The exposure status was based on annual birth rates and did not account for temporal variations. |
| 10. Was the exposure(s) assessed more than once over time? |  | No |  |  |
| 11. Were the outcome measures (dependent variables) clearly defined, valid, reliable, and implemented consistently across all study participants? | Yes |  |  |  |
| 12. Were the outcome assessors blinded to the exposure status of participants? |  |  | Not Reported |  |
| 13. Was loss to follow-up after baseline 20% or less? |  |  | Not Reported |  |
| 14. Were key potential confounding variables measured and adjusted statistically for their impact on the relationship between exposure(s) and outcome(s)? | Yes |  |  |  |
| Quality Rating |  | Fair |  |  |
| Additional Comments (If Poor, please state why):  The study is ranked as fair in relation to interpretations regarding midwife staffing levels, which is not the primary focus. Midwife staffing ratios to birth are measured annually and therefore don’t account for random peaks in demand that occur daily/weekly. The covariates don’t include other staff rates on the ward which may confound the relationship between workload and outcomes. | | | |  |
| Data extractor Richard Mattock  Date of extraction 09 September 2022 | | | |  |

| **Draper (2017)** | | | | |
| --- | --- | --- | --- | --- |
| ***Major Components*** | ***Response options*** | | | ***Notes*** |
| 1. Was the research question or objective in this paper clearly stated? | Yes |  |  |  |
| 2. Was the study population clearly specified and defined? | Yes |  |  |  |
| 3. Was the participation rate of eligible persons at least 50%? |  |  | NA |  |
| 4. Were all the subjects selected or recruited from the same or similar populations (including the same time period)? Were inclusion and exclusion criteria for being in the study prespecified and applied uniformly to all participants? | Yes |  |  |  |
| 5. Was a sample size justification, power description, or variance and effect estimates provided? |  |  | NA | No effect sizes estimated. |
| 6. For the analyses in this paper, were the exposure(s) of interest measured prior to the outcome(s) being measured? |  |  | Not Reported |  |
| 7. Was the timeframe sufficient so that one could reasonably expect to see an association between exposure and outcome if it existed? |  |  | Not Reported |  |
| 8. For exposures that can vary in amount or level, did the study examine different levels of the exposure as related to the outcome (e.g., categories of exposure, or exposure measured as continuous variable)? |  |  | Not Reported |  |
| 9. Were the exposure measures (independent variables) clearly defined, valid, reliable, and implemented consistently across all study participants? |  | No |  |  |
| 10. Was the exposure(s) assessed more than once over time? |  |  | Not Reported |  |
| 11. Were the outcome measures (dependent variables) clearly defined, valid, reliable, and implemented consistently across all study participants? | Yes |  |  |  |
| 12. Were the outcome assessors blinded to the exposure status of participants? |  |  | NA | No counterfactual so blinding not possible. |
| 13. Was loss to follow-up after baseline 20% or less? |  |  | Not Reported |  |
| 14. Were key potential confounding variables measured and adjusted statistically for their impact on the relationship between exposure(s) and outcome(s)? |  | No |  |  |
| Quality Rating |  |  | Poor |  |
| Additional Comments (If Poor, please state why):  Study is a review of case studies and no counterfactual is included. There are no analytical methods used to estimate associations, and the exposure status (i.e. staffing variable) is not described in any detail. | | | |  |
| Data extractor Richard Mattock  Date of extraction 09 September 2022 | | | |  |

| **Turner (2022)** | | | | |
| --- | --- | --- | --- | --- |
| ***Major Components*** | ***Response options*** | | | ***Notes*** |
| 1. Was the research question or objective in this paper clearly stated? | Yes |  |  |  |
| 2. Was the study population clearly specified and defined? |  | No |  | No indication of inclusion/exclusion criteria |
| 3. Was the participation rate of eligible persons at least 50%? |  | No |  | Overall response rate = 37% |
| 4. Were all the subjects selected or recruited from the same or similar populations (including the same time period)? Were inclusion and exclusion criteria for being in the study prespecified and applied uniformly to all participants? | Yes |  |  |  |
| 5. Was a sample size justification, power description, or variance and effect estimates provided? |  | No |  | Sample size was sufficiently large for statistical power |
| 6. For the analyses in this paper, were the exposure(s) of interest measured prior to the outcome(s) being measured? |  | No |  | Cross sectional design using annual measures |
| 7. Was the timeframe sufficient so that one could reasonably expect to see an association between exposure and outcome if it existed? | Yes |  |  |  |
| 8. For exposures that can vary in amount or level, did the study examine different levels of the exposure as related to the outcome (e.g., categories of exposure, or exposure measured as continuous variable)? | Yes |  |  | Midwifery staffing measured as continuous variable and categorical variable |
| 9. Were the exposure measures (independent variables) clearly defined, valid, reliable, and implemented consistently across all study participants? |  | No |  | The exposure status was based on ratios of midwives to births at national levels reported annually. |
| 10. Was the exposure(s) assessed more than once over time? |  | No |  |  |
| 11. Were the outcome measures (dependent variables) clearly defined, valid, reliable, and implemented consistently across all study participants? |  | No |  | Self-reported measures used to identify length of stay |
| 12. Were the outcome assessors blinded to the exposure status of participants? |  | No |  | Secondary cross sectional analysis |
| 13. Was loss to follow-up after baseline 20% or less? |  |  | Not Reported |  |
| 14. Were key potential confounding variables measured and adjusted statistically for their impact on the relationship between exposure(s) and outcome(s)? | Yes |  |  |  |
| Quality Rating |  |  | Poor |  |
| Additional Comments (If Poor, please state why):  The study is ranked as poor in relation to interpretations regarding midwife staffing levels. Midwife staffing ratios to birth are measured annually and therefore don’t account for random peaks in demand that occur daily/weekly. The outcome measure for delay in discharge is self-report rather than length of stay e.g. from routine hospital data, and response rates are low. | | | |  |
| Data extractor Richard Mattock  Date of extraction 11 July 2024 | | | |  |

| **Vanderlaan (2023)** | | | | |
| --- | --- | --- | --- | --- |
| ***Major Components*** | ***Response options*** | | | ***Notes*** |
| 1. Was the research question or objective in this paper clearly stated? | Yes |  |  |  |
| 2. Was the study population clearly specified and defined? | Yes |  |  |  |
| 3. Was the participation rate of eligible persons at least 50%? |  |  | N/A |  |
| 4. Were all the subjects selected or recruited from the same or similar populations (including the same time period)? Were inclusion and exclusion criteria for being in the study prespecified and applied uniformly to all participants? | Yes |  |  |  |
| 5. Was a sample size justification, power description, or variance and effect estimates provided? |  | No |  | Sample size was sufficiently large for statistical power |
| 6. For the analyses in this paper, were the exposure(s) of interest measured prior to the outcome(s) being measured? |  | No |  | Cross sectional design using annual measures |
| 7. Was the timeframe sufficient so that one could reasonably expect to see an association between exposure and outcome if it existed? | Yes |  |  |  |
| 8. For exposures that can vary in amount or level, did the study examine different levels of the exposure as related to the outcome (e.g., categories of exposure, or exposure measured as continuous variable)? | Yes |  |  | 3 categories (no midwives, low level, high level) |
| 9. Were the exposure measures (independent variables) clearly defined, valid, reliable, and implemented consistently across all study participants? |  | No |  | The exposure status was based on ratios of midwives to births at the county level so did not account for temporal and ward-level variations. |
| 10. Was the exposure(s) assessed more than once over time? |  | No |  |  |
| 11. Were the outcome measures (dependent variables) clearly defined, valid, reliable, and implemented consistently across all study participants? | Yes |  |  |  |
| 12. Were the outcome assessors blinded to the exposure status of participants? |  | No |  | Secondary cross sectional analysis |
| 13. Was loss to follow-up after baseline 20% or less? |  |  | Not Reported |  |
| 14. Were key potential confounding variables measured and adjusted statistically for their impact on the relationship between exposure(s) and outcome(s)? | Yes |  |  |  |
| Quality Rating |  | Fair |  |  |
| Additional Comments (If Poor, please state why):  The study is ranked as fair in relation to interpretations regarding midwife staffing levels. Midwife staffing ratios to birth are measured annually and therefore don’t account for random peaks in demand that occur daily/weekly. The ratio also uses an arbitrary cut-off where top 10% classified as high midwife density whilst 80% is classified as low midwife density. | | | |  |
| Data extractor Richard Mattock  Date of extraction 11 July 2024 | | | |  |

| **Zbiri (2018)** | | | | |
| --- | --- | --- | --- | --- |
| ***Major Components*** | ***Response options*** | | | ***Notes*** |
| 1. Was the research question or objective in this paper clearly stated? | Yes |  |  |  |
| 2. Was the study population clearly specified and defined? | Yes |  |  |  |
| 3. Was the participation rate of eligible persons at least 50%? | Yes |  |  |  |
| 4. Were all the subjects selected or recruited from the same or similar populations (including the same time period)? Were inclusion and exclusion criteria for being in the study prespecified and applied uniformly to all participants? | Yes |  |  |  |
| 5. Was a sample size justification, power description, or variance and effect estimates provided? |  | No |  | Very large sample size, should have adequate power. |
| 6. For the analyses in this paper, were the exposure(s) of interest measured prior to the outcome(s) being measured? |  |  | Don’t know | Staffing was and aggregated annual measure of FTE per 100 births. Aggregated measure may have included observations prior to delivery. |
| 7. Was the timeframe sufficient so that one could reasonably expect to see an association between exposure and outcome if it existed? | Yes |  |  |  |
| 8. For exposures that can vary in amount or level, did the study examine different levels of the exposure as related to the outcome (e.g., categories of exposure, or exposure measured as continuous variable)? | Yes |  |  | Staffing was measured as a continuous ratio i.e. FTE per 100 births. |
| 9. Were the exposure measures (independent variables) clearly defined, valid, reliable, and implemented consistently across all study participants? |  |  | Don’t know | This is not clear, insufficient information provided. |
| 10. Was the exposure(s) assessed more than once over time? | Yes |  |  | Aggregated annual measures were obtained, it is likely (although not stated) that these were measured more than once as the study period covered multiple years. |
| 11. Were the outcome measures (dependent variables) clearly defined, valid, reliable, and implemented consistently across all study participants? | Yes |  |  |  |
| 12. Were the outcome assessors blinded to the exposure status of participants? |  |  | Not Reported |  |
| 13. Was loss to follow-up after baseline 20% or less? | Yes |  |  |  |
| 14. Were key potential confounding variables measured and adjusted statistically for their impact on the relationship between exposure(s) and outcome(s)? | Yes |  |  | Some potential issues with included both FTE per 100 births and size of maternity unit (measured as number of births per year) in the same regression model. Potential for multi collinearity. |
| Quality Rating |  |  | Poor |  |
| Additional Comments (If Poor, please state why):  The study is ranked as poor in relation to interpretations regarding staffing levels. The staffing variable is an annual aggregate measure for births and staff number and therefore does not account for varied demand within the year. There are also issues with the empirical equations, where births per year is included twice, first directly as a measure of “size of the maternity unit”, second indirectly through the staff variable FTE per 100 births. | | | |  |
| Data extractor Richard Mattock  Date of extraction 15 September 2022 | | | |  |

| **Robertson et al. (2021)** | | | |  |
| --- | --- | --- | --- | --- |
| ***Major Components*** | ***Response*** | | | ***Notes*** |
| 1. Was the research question or objective in this paper clearly stated? | Yes |  |  |  |
| 2. Was the study population clearly specified and defined? | Yes |  |  |  |
| 3. Was the participation rate of eligible persons at least 50%? |  |  | Not Reported |  |
| 4. Were all the subjects selected or recruited from the same or similar populations (including the same time period)? Were inclusion and exclusion criteria for being in the study prespecified and applied uniformly to all participants? | Yes |  |  |  |
| 5. Was a sample size justification, power description, or variance and effect estimates provided? |  | No |  | Large sample power should be sufficient. |
| 6. For the analyses in this paper, were the exposure(s) of interest measured prior to the outcome(s) being measured? | Yes |  |  |  |
| 7. Was the timeframe sufficient so that one could reasonably expect to see an association between exposure and outcome if it existed? | Yes |  |  |  |
| 8. For exposures that can vary in amount or level, did the study examine different levels of the exposure as related to the outcome (e.g., categories of exposure, or exposure measured as continuous variable)? |  | No |  |  |
| 9. Were the exposure measures (independent variables) clearly defined, valid, reliable, and implemented consistently across all study participants? | Yes |  |  | Birthrate plus tool used to measure shortfall on wards at time of delivery, adjusted for case mix. |
| 10. Was the exposure(s) assessed more than once over time? |  |  | Not reported |  |
| 11. Were the outcome measures (dependent variables) clearly defined, valid, reliable, and implemented consistently across all study participants? | Yes |  |  |  |
| 12. Were the outcome assessors blinded to the exposure status of participants? |  |  | Not Reported |  |
| 13. Was loss to follow-up after baseline 20% or less? |  |  | Not Reported |  |
| 14. Were key potential confounding variables measured and adjusted statistically for their impact on the relationship between exposure(s) and outcome(s)? |  | No |  | There were no covariates included in the analyses. |
| Quality Rating |  |  | Poor |  |
| Additional Comments (If Poor, please state why):  The analysis was not adjusted for any covariates, however the measurement of staffing was good and unlikely to be prone to measurement error. | | | | |
| Data extractor Richard Mattock  Date of extraction 20 March 2025 | | | | |

| **Lyndon et al. (2022)** | | | |  |
| --- | --- | --- | --- | --- |
| ***Major Components*** | ***Response*** | | | ***Notes*** |
| 1. Was the research question or objective in this paper clearly stated? | Yes |  |  |  |
| 2. Was the study population clearly specified and defined? | Yes |  |  |  |
| 3. Was the participation rate of eligible persons at least 50%? |  | No |  |  |
| 4. Were all the subjects selected or recruited from the same or similar populations (including the same time period)? Were inclusion and exclusion criteria for being in the study prespecified and applied uniformly to all participants? | Yes |  |  |  |
| 5. Was a sample size justification, power description, or variance and effect estimates provided? |  | No |  | Large sample power should be sufficient. |
| 6. For the analyses in this paper, were the exposure(s) of interest measured prior to the outcome(s) being measured? |  |  | Not clear | Survey of nurse perception of staffing was collected separately to the outcome measure, the timing of these measures is unclear. |
| 7. Was the timeframe sufficient so that one could reasonably expect to see an association between exposure and outcome if it existed? | Yes |  |  |  |
| 8. For exposures that can vary in amount or level, did the study examine different levels of the exposure as related to the outcome (e.g., categories of exposure, or exposure measured as continuous variable)? |  | No |  |  |
| 9. Were the exposure measures (independent variables) clearly defined, valid, reliable, and implemented consistently across all study participants? |  | No |  | Staffing was a subjective response from a nurses survey, the study did not analyse time variable nature of staffing at different delivery times (i.e. used same score across who study period) |
| 10. Was the exposure(s) assessed more than once over time? |  | No |  |  |
| 11. Were the outcome measures (dependent variables) clearly defined, valid, reliable, and implemented consistently across all study participants? | Yes |  |  |  |
| 12. Were the outcome assessors blinded to the exposure status of participants? |  |  | Not Reported |  |
| 13. Was loss to follow-up after baseline 20% or less? |  |  | Not Reported |  |
| 14. Were key potential confounding variables measured and adjusted statistically for their impact on the relationship between exposure(s) and outcome(s)? | Yes |  |  | . |
| Quality Rating |  |  | Poor |  |
| Additional Comments (If Poor, please state why):  The statistical methods appear appropriate, and were adjusted for relevant covariates. However the staffing measure is prone to measurement error as it was subjective based on nurses’ interpretation of guidelines, and was an aggregate i.e. average measure of staffing across the study period. | | | | |
| Data extractor: Richard Mattock  Date of extraction: 20 March 2025 | | | | |

| **Wilson et al. (2021)** | | | |  |
| --- | --- | --- | --- | --- |
| ***Major Components*** | ***Response*** | | | ***Notes*** |
| 1. Was the research question or objective in this paper clearly stated? | Yes |  |  |  |
| 2. Was the study population clearly specified and defined? | Yes |  |  |  |
| 3. Was the participation rate of eligible persons at least 50%? |  |  | Not Reported |  |
| 4. Were all the subjects selected or recruited from the same or similar populations (including the same time period)? Were inclusion and exclusion criteria for being in the study prespecified and applied uniformly to all participants? |  |  | Not reported |  |
| 5. Was a sample size justification, power description, or variance and effect estimates provided? |  |  | Not reported | Sample power should be sufficient. |
| 6. For the analyses in this paper, were the exposure(s) of interest measured prior to the outcome(s) being measured? |  |  | Not reported |  |
| 7. Was the timeframe sufficient so that one could reasonably expect to see an association between exposure and outcome if it existed? |  |  | Not reported |  |
| 8. For exposures that can vary in amount or level, did the study examine different levels of the exposure as related to the outcome (e.g., categories of exposure, or exposure measured as continuous variable)? | Yes |  |  | Multiple specifications for the exposure variable were used in regression equations |
| 9. Were the exposure measures (independent variables) clearly defined, valid, reliable, and implemented consistently across all study participants? |  |  | Unclear | No details are provided on when the exposure variable was measured. Robustness checks are performed for yesterdays staffing hours so it might be assumed staffing hours were measured at the time of delivery. |
| 10. Was the exposure(s) assessed more than once over time? |  |  | Not reported |  |
| 11. Were the outcome measures (dependent variables) clearly defined, valid, reliable, and implemented consistently across all study participants? |  |  | Not reported | No details were provided on how outcomes were measured |
| 12. Were the outcome assessors blinded to the exposure status of participants? |  |  | Not Reported |  |
| 13. Was loss to follow-up after baseline 20% or less? |  |  | Not Reported |  |
| 14. Were key potential confounding variables measured and adjusted statistically for their impact on the relationship between exposure(s) and outcome(s)? | Yes |  |  |  |
| Quality Rating |  | Fair |  |  |
| Additional Comments (If Poor, please state why):  In general the analysis appeared to be of good quality but the level of reporting was poor in terms of the sample used and how exposure and outcomes were measured. | | | | |
| Data extractor: Richard Mattock  Date of extraction: 21 March 2025 | | | | |

| **Knape et al. (2014)** | | | |  |
| --- | --- | --- | --- | --- |
| ***Major Components*** | ***Response*** | | | ***Notes*** |
| 1. Was the research question or objective in this paper clearly stated? | Yes |  |  |  |
| 2. Was the study population clearly specified and defined? | Yes |  |  |  |
| 3. Was the participation rate of eligible persons at least 50%? | Yes |  |  |  |
| 4. Were all the subjects selected or recruited from the same or similar populations (including the same time period)? Were inclusion and exclusion criteria for being in the study prespecified and applied uniformly to all participants? | Yes |  |  |  |
| 5. Was a sample size justification, power description, or variance and effect estimates provided? | Yes |  |  |  |
| 6. For the analyses in this paper, were the exposure(s) of interest measured prior to the outcome(s) being measured? | Yes |  |  |  |
| 7. Was the timeframe sufficient so that one could reasonably expect to see an association between exposure and outcome if it existed? | Yes |  |  |  |
| 8. For exposures that can vary in amount or level, did the study examine different levels of the exposure as related to the outcome (e.g., categories of exposure, or exposure measured as continuous variable)? |  | No |  |  |
| 9. Were the exposure measures (independent variables) clearly defined, valid, reliable, and implemented consistently across all study participants? |  | No |  | The measure of midwife workload was based on midwives having a single case during their shift or more than one case. This did not account for whether cases occurred simultaneously or how long each case occurred for. |
| 10. Was the exposure(s) assessed more than once over time? |  | No |  |  |
| 11. Were the outcome measures (dependent variables) clearly defined, valid, reliable, and implemented consistently across all study participants? | Yes |  |  |  |
| 12. Were the outcome assessors blinded to the exposure status of participants? |  |  | Not Reported |  |
| 13. Was loss to follow-up after baseline 20% or less? | Yes |  |  |  |
| 14. Were key potential confounding variables measured and adjusted statistically for their impact on the relationship between exposure(s) and outcome(s)? |  | No |  | No covariates were included for the outcome of interest (mode of birth) as the model was a backwards logistic regression. This means non-significant variables are removed and therefore workload results are only reported for bivariate (unadjusted) analyses. |
| Quality Rating |  | Fair |  |  |
| Additional Comments (If Poor, please state why):  The analysis was not adjusted for any covariates, the measurement of staffing could also be influenced by timing of deliveries i.e. higher workload could represent low workload in some cases if deliveries do not overlap during shifts. | | | | |
| Data extractor: Richard Mattock  Date of extraction: 24 March 2025 | | | | |
