# Supplementary material for: The impact of midwife workload on delivery of care, and mother and baby outcomes in maternity settings in OECD countries: A systematic review
Source: PLoS One. 2025 Aug 25;20(8):e0329117. doi: 10.1371/journal.pone.0329117 (PMC12377604; doi:10.1371/journal.pone.0329117)
Supplement: S5 File — (DOCX) [file pone.0329117.s005.docx]

# Supporting Information S5 Data Extraction Tables RQ2 & RQ3

| **Author** | | **Facchini (2022)** |
| --- | --- | --- |
| Design | | Estimates the impact of workload, the ratio of patients to midwives (RPM), on probability of getting a caesarean section. The second analysis looks at whether physician induced demand plays a role in differential treatment between patients admitted during high and low staffing. Hypothesis is that physicians may find it easier to offer treatment that reduce midwife workload (i.e. caesarean section) to some patients than others. The study estimates the impact of workload on caesarean section using an interaction term between mother’s civil status (married and unmarried) and the RPM.  The study also includes multiple covariates: those related to the mother and the pregnancy; the number of gynaecologists scheduled to be in the delivery room at the time of admission; and shift, day of the week, month and year of admission. |
| Findings: direct assessment | Sub-samples | Not applicable |
|  | Interaction terms | Single mothers have an increasing probability of caesarean section for higher workload levels, while married mothers have almost no association between the two variables:  The RPM*Married coefficient is -0.076 (p<0.01), the coefficient remains consistent and statistically significant in samples which: only include: healthy mothers, young mothers, not first-time mothers, mothers with university degrees.  The average marginal effects: Single and married patients observe a similar rate of C-section when observing workload at the 10th percentile (8.9% and 8.6% respectively), single patients are 42% more likely to have a C-section than married patients if admitted when workload is at the 90th percentile (~13.8% and ~9.9% respectively).  Overall the population who are married do not have underlying characteristics that would be expected to decrease probability of caesarean section e.g. they are older, and less likely to be first-time mothers, and more likely to hold a tertiary degree. |
| Findings: covariates | Not associated with outcome | Not reported |
|  | Associated with outcome | Not reported |
|  | Associated with staffing | Not reported |
|  | Not associated with staffing | Not reported |
| Interpretation | | Patients admitted during high midwife workload are more likely to get a caesarean section. This change in delivery method could be explained by worsening patient health due to lower midwife care or by physician induced demand. Evidence for the latter as single and married women have the same probability of caesarean section when workload is low, and likelihood increases with workload for single women only. It is assumed that single women are more likely to be alone in the delivery room and therefore easier to convince. |
| Data extractor | | Richard Mattock |
| Date of extraction | | 9 September 2022 |
| Eligible RQ2 | | Yes |
| Eligible RQ3 | | No |

| **Author** | | **Freeman (2016)** |
| --- | --- | --- |
| Design | | Impact of workload (staffing) on epidural rates and specialist referral for physician led delivery are estimated separately for complex and non-complex cases. Non complex cases are defined as those with spontaneous onset of labour. Complex cases are those whose labour is pharmacologically induced in hospital prior to arrival at the delivery unit.  The study also includes a wide range of covariates, related to mother and the pregnancy, time-related factors, medical complications during delivery, contextual factors and operational factors. |
| Findings: direct assessment | Sub-samples | Main econometric results, reported for the average partial marginal effect per one standard deviation increase in midwife workload.   - For rates of epidural: -0.025 (p<0.001) for the whole sample; -0.025 (p<0.001) for non-complex cases; and -0.006 (p= 0.415) for complex cases. - For referral rate to obstetrician: 0.002 (p=0.497) for the whole sample; -0.006 (p>0.10) for non-complex cases; 0.015 (p<0.05) for complex cases.   Illustrative results obtained by comparing rates for two standard deviations below and above mean for workload:   - For noncomplex cases, epidural rates fall from 32% at low workload to 22.8% at high workload. Reduction in epidural rate lead directly to increase in referral rates. - For complex cases, no significant effect on epidural rate, referral rate increases from 37.1% to 42.3% as workload increases.   OLS results for clinical outcomes:   - Post birth length of stay: Decreases by 8.3% at high workloads for non-complex cases due to reduction in epidural rates; no effect in complex cases - Apgar score: No effect for complex and non-complex cases. - Perineal tear: Non-complex cases no effect APE 0.001 (p>0.10); in complex cases rates increase with increased workload APE 0.011 (p<0.01). |
|  | Interaction terms | The supplementary appendix include interaction terms between workload and complexity, rather than conducting analyses in sub samples. Results are consistent with those reported in the main paper. |
| Findings: covariates | Not associated with outcome | Temporal: daily trend, daily trend squared, year quarter, weekend birth  Clinical complications: Shoulder dystocia, obstructed labour, COPD  Contextual factors: deprivation index, health index, distance to hospital, antenatal stay  Other operational factors: proportion epidural, physician-led, proportion escalated, post birth workload, post birth workload |
|  | Associated with outcome | Mother: age, BMI, number of previous births, age of first birth, previous caesarean section  Pregnancy: Gestation, baby weight, baby weight squared  Temporal: hour of birth  Clinical complications: Malpresentation, diabetes, hypertension, “other”, PROM  Contextual factors: number of antenatal visits |
|  | Associated with staffing | Not reported |
|  | Not associated with staffing | Not reported |
| Interpretation | | Workload appears to affect midwife decision making, and this is dependent on case complexity. Midwives may make use of two levers to manage workload: first rationing resource-intensive discretionary care (epidural analgesia) for non-complex cases, second increasing rates of specialist referral (physician led deliver) for complex cases.  The impact of higher workload on outcomes is likely to be complex. It is possible that changes in midwife decision making in higher workload situations is beneficial/more efficient. E.g. if analgesia is typically over prescribed for women who don’t need it, and if complex patients don’t get referred to physicians who should (e.g. physicians may be more likely to accept patients when midwives have very high workloads). |
| Data extractor | | Richard Mattock |
| Date of extraction | | 9 September 2022 |
| Eligible RQ2 | | Yes |
| Eligible RQ3 | | No |

| **Author** | | **Hollowell (2015)** |
| --- | --- | --- |
| Design | | The impact of the staffing variables was estimated separately for samples of nulliparous and multiparous women. |
| Findings: direct assessment | Sub-samples | Results report the regression coefficient and (p-value) between understaffing and outcomes:  Results for  Obstetric units:  Nulliparous:   - Instrumental delivery: 0.02 (p= 0.798) - **Intrapartum caesarean section: -0.10 (p= 0.025)** - Straightforward birth: 0.06 (p= 0.307) - Normal birth: -0.01 (p= 0.889) - Epidural 0.05 (0.594) - Augmentation -0.10 (0.156)   Multiparous:   - Instrumental delivery: -0.04 (p= 0.068) - Intrapartum caesarean section: -0.05 (p= 0.106) - **Straightforward birth: 0.08 (p= 0.011)** - Normal birth: 0.05 (p= 0.482) - Epidural 0.0 (0.942) - **Augmentation -0.09 (0.048)**   Results for AMUs:  Nulliparous:   - Instrumental delivery: 0.17 (p= 0.483) - Intrapartum caesarean section: 0.05 (p= 0.511) - Straightforward birth: -0.28 (p= 0.255) - Normal birth: -0.34 (p= 0.254) - Transfer: 0.2 (p = 0.415)   Multiparous:   - Instrumental delivery: 0.04 (p= 0.471) - Intrapartum caesarean section: 0.00 (p= 0.956) - Straightforward birth: -0.06 (p= 0.496) - Normal birth: 0.03 (p= 0.735) - Transfer: -0.1 (p =0.569)   Results for FMUs:  Nulliparous:   - Instrumental delivery: -0.17 (p= 0.455) - Intrapartum caesarean section: 0.05 (p= 0.670) - Straightforward birth: 0.11 (p= 0.685) - Normal birth: -0.33 (p= 0.222)   Multiparous:   - Instrumental delivery: -0.01 (p= 0.847) - Intrapartum caesarean section: -0.05 (p= 0.046) - Straightforward birth: 0.07 (p= 0.462) - Normal birth: 0.13 (p= 0.119) |
|  | Interaction terms | Not applicable |
| Findings: covariates | Not associated with outcome | Not applicable |
|  | Associated with outcome | Not applicable |
|  | Associated with staffing | Not applicable |
|  | Not associated with staffing | Not applicable |
| Interpretation | | Observed some significant associations, these showed no consistent pattern, suggesting that there is no straightforward or clear relationship between midwifery staffing intervention rates. There was no association between understaffing and intervention rates in FMUs or AMUs. There was no consistent findings of differences between nulliparous and multiparous women with respect to the impact of staffing on outcomes. |
| Data extractor | | Richard Mattock |
| Date of extraction | | 9 September 2022 |
| Eligible RQ2 | | Yes |
| Eligible RQ3 | | No |

| **Author** | | **Kpéa (2022)** |
| --- | --- | --- |
| Design | | The study assesses the impact of multiple factors, including staffing on the administration of neuraxial analgesia during birth.  The study includes multiple covariates in addition to staffing: factors relating to mothers including demographics, medical conditions, prenatal care; factors relating to the delivery; and factors relating to the maternity unit. |
| Findings: direct assessment | Sub-samples | Not applicable |
|  | Interaction terms | Not applicable |
| Findings: covariates | Not associated with outcome | Mother: maternal age, education level, family status, adverse obstetrical history, chronic medical conditions, medical disorder during pregnancy  Care provision: prenatal care (adequate/ inadequate), childbirth education  Delivery: vaginal instrumental delivery  Maternity unit: public/private. |
|  | Associated with outcome | Mother: gestational age, parity  Delivery: Oxytocin augmentation of labour  Maternity Unit: Anaesthesiologist availability (No, Yes in hospital, Yes in obstetric department) |
|  | Associated with staffing | Not reported |
|  | Not associated with staffing | Not reported |
| Interpretation | | No discussion is provided regarding the impact of covariates to modify the relationship between staffing and administration of neuraxial analgesia. |
| Data extractor | | Richard Mattock |
| Date of extraction | | 9 September 2022 |
| Eligible RQ2 | | Yes |
| Eligible RQ3 | | Yes |

| **Author** | | **Zbiri (2018)** |
| --- | --- | --- |
| Design | | The study assesses the impact of multiple factors, including staffing on caesarean section (emergency, elective, and intrapartum)  The study includes multiple covariates in addition to staffing: factors relating to mothers including demographics, medical conditions, factors relating to the delivery; factors relating the baby; and factors relating to the maternity unit. |
| Findings: direct assessment | Sub-samples | Not applicable |
|  | Interaction terms | Not applicable |
| Findings: covariates | Not associated with outcome | Intrapartum caesarean section:  Maternity unit : Private/public hospital, teaching status of hospital, level of neonatal care (no unit, neonatal unit, intensive care neonatal unit), weekend/holiday delivery, on call obstetrician outside of unit, anaesthesiologist staffing level (FTEs per 100 deliveries) |
|  | Associated with outcome | Intrapartum caesarean section:  Individual level: Maternal age, parity, previous caesarean section, medical risk condition, preterm delivery, breech/transverse presentation, induced labour and birth weight,  Maternity Unit: size of the maternity unit (<1000, 1000-1999, >= 2000 deliveries per year); obstetrician staffing level (FTEs per 100 deliveries) |
|  | Associated with staffing | Not reported |
|  | Not associated with staffing | Not reported |
| Interpretation | | No discussion is provided regarding the impact of covariates to modify the relationship between staffing and caesarean section. |
| Data extractor | | Richard Mattock |
| Date of extraction | | 15 September 2022 |
| Eligible for RQ2 | | Yes |
| Eligible for RQ3 | | Yes |

| **Author** | | **Lyndon (2022)** |
| --- | --- | --- |
| Design | | The study analyses the effect of staffing scores, measured using nurse survey data on adherence to staffing guidelines on exclusive breast-feeding rates. A Generalized structural equation model was used to model the mediating impact of “missed care” between staffing and breast feeding. Missed care was obtained from a nurses survey and is the summed total of 25 items on the MISSCARE survey. |
| Findings: direct assessment | Sub-samples | Not applicable |
|  | Mediation analysis | Overall perinatal missed care (sum of all items) did not significantly mediate the association between staffing guideline adherence and EBMF rate (p = 0.236).  Two aspects of perinatal missed care related to breastfeeding showed partial mediation. Missed skin-to-skin mother–baby care after birth had a significant indirect effect (β = 0.035, p = 0.003), and missed breastfeeding within 1 hour after birth had a significant indirect effect (β = 0.029, p =0.007). The indirect effects for these two elements were significant, whilst the direct result between staffing and breast feeding remained indicating partial mediation. |
| Findings: covariates | Not associated with outcome | Hospital factors:   - Urban rural characteristics of the hospital (p = 0.306) - Teaching hospital (p = 0.161) - Critical hospital (p =0.256) - Sole community provider (p = 0.461) - Obstetric level (uncomplicated/ complicated/ serious) p =0.068 - Neonatal intensive care hospital (p =0.226)   Staffing factors:   - Safety climate on nurses survey (β = 0.051, p = 0.091) - Mean age (p = 0.101) - Mean years of experience (p = 0.170) - Nurse education (p = 0.076) |
|  | Associated with outcome | Hospital factors   - Ownership (government/church/non profit) p = 0.017 - Annual birth volume (β = -0.014, p = 0.019) |
|  | Associated with staffing | N/A |
|  | Not associated with staffing | N/A |
| Interpretation | | There was no significant mediation identified for the overall missed care variable. Some aspects of missed care related to mother-baby interaction appear to mediate relationship between staffing and exclusive breastfeeding i.e. missed skin-to-skin contact and missed breastfeeding within 1-hour of delivery. |
| Data extractor | | Richard Mattock |
| Date of extraction | | 20 March 2025 |
| Eligible for RQ2 | | No |
| Eligible for RQ3 | | Yes |
