# Supplementary material for: The impact of midwife workload on delivery of care, and mother and baby outcomes in maternity settings in OECD countries: A systematic review
Source: PLoS One. 2025 Aug 25;20(8):e0329117. doi: 10.1371/journal.pone.0329117 (PMC12377604; doi:10.1371/journal.pone.0329117)
Supplement: S6 File — (DOCX) [file pone.0329117.s006.docx]

# Supporting Information S6. Summary of Included Studies

Dani et al. (2020) (17) conducted a cohort study to establish the effect of midwife-to-infant ratios on outcomes for healthy term infants, in a hospital led midwife centre and an obstetrician led centre in Florence, Italy. Recruitment occurred between January and June 2018, with 220 included from the study, 110 from each centre. A midwifery workload variable was not directly measured, instead comparisons were made between centres assuming effects based on differences in midwife to infant ratios which were stated as being equal to 1:2.5 or 1:5 (midwife led centre); and 1:7 or 1:9 (obstetrician led centre). Using logistic regression the study identified significantly higher rates of exclusive breastfeeding, and reduced hospital stay and admission to neonatal care for the midwife led centre. The study was ranked as “poor” quality as the midwife workload variable was not directly measured, and differences could be due to non-staffing factors, e.g. women self-select to centre of choice, so there may be different underlying risks in each population.

Facchini (2022) (18) conducted a cohort study investigating the relationship between midwife workload and rates of emergency C-section, and maternal and neonatal outcomes in a maternity unit in Tuscany, Italy. The population included 6,142 births between 2011 and 2014 from spontaneous onset of labour and excluded elective C-sections and inductions. Workload was measured as the ratio of patients to midwives at the time of admission, as a binary (above/ below 20th percentile) and continuous variable. The number of midwives was not directly measured but assumed equivalent to staff shift schedules. The linear probability model identified a significant increase in C-section for (binary) high versus normal workload. There was a 21% increase in the probability of post-partum haemorrhage for high versus normal workload, which was almost entirely driven by the increase in C-section. An interaction term between marital status and workload was significant, single mothers had a significantly higher rate of C-section at high midwife workload, but married women were unaffected. There was no significant association for workload measured as a continuous variable. The study was ranked as good quality with low risk of bias.

Freeman et al. (2017) (19) conducted a cohort study investigating the relationship between service demand for midwives, and delivery, maternal and baby outcomes by maternal clinical risk. The study sample was 16,355 births from the maternity department of a large UK teaching hospital between 2008 and 2013. The population excluded elective C-sections, transfers, and very high-risk patients. The staffing variable was workload, a standardized time weighted average number of patients per midwife for three hours prior to birth, measured using real time data in the delivery unit. The primary outcome variables were probability of epidurals and referral to obstetricians both estimated separately by clinical risk. Multiple maternal and neonatal secondary outcomes were also included. The Probit model indicated that high workload was associated with lower epidural rates for non-complex cases with no impact on complex cases. Higher rates of referral were observed for complex cases whereas non-complex cases were less likely to be referred. No effect were found on Apgar score. Moving from low to high workload resulted in an 8.3% decrease in post-birth length of stay due to reductions in epidural rates. The study was ranked as good quality with low risk of bias.

Hollowell et al. (2015) (20) conducted several analyses using data from the Birthplace in England prospective cohort study. The sample included women attended by NHS midwives during labour, and excluded planned C-section, preterm labour, multiple pregnancy, and stillbirth. The relevant analysis (study 3) explored the impact of NHS trust factors on maternal and neonatal outcomes, in a sub-sample of low-risk term pregnancies comprising 16,753 planned in alongside midwifery units, 11,210 in freestanding midwifery units, and 16,632 home births. Midwife workload was measured using logs, completed on the wards twice daily. The study reported the mean number of midwives on duty per woman in labour and also included an “understaffing” variable derived by calculating the % of days per year that the number of births exceed the number of midwives on duty. Both staffing variables were trust level aggregate measures i.e. mean values across trusts that did not differ by individuals. Simple linear regression did not identify any consistent associations between staffing and delivery mode. Higher staffing rates were associated with significantly increased transfer but only for multiparous births, but no effects were identified for the “understaffing” variable. The study was ranked as poor quality with high risk of bias as the staffing variable was a condensed annual measure, and the analysis did not adjust for covariates.

Isidore & Rousseau (2018) (21) conducted a cross-sectional study of 204 French midwives in 2015 and 2016 investigating the relationship between staffing levels and oxytocin administration during birth. The study involved an online survey asking midwives to describe the treatment they would provide for different birth related case vignettes. A separate questionnaire identified midwife characteristics including staffing factors. The staffing variable was derived from questionnaire responses and was the number of annual births divided by the number of midwives working daily. The study found that midwives with higher workload were more likely to recommend administering oxytocin. In 78% of cases, oxytocin was not indicated for the case vignette. The study was rated as “poor” quality as the outcome variable was not observed (i.e. midwives only stated what they hypothetically would do) and no covariates were included in the analysis.

Kpéa et al. (2015) (22) used cross sectional data from the 2010 French National Perinatal Survey to investigate reasons for neuraxial analgesia during labour. The sample included 7,123 low risk women who had normal vaginal deliveries, and excluded C-sections, inductions, births resulting in foetal death and medical contraindications for neuraxial analgesia. The sample was split into women with preference for labour with (26%) and without neuraxial analgesia (74%). Midwife workload was included as a covariate and measured as the ratio of midwives per shift in the labour ward to the number of annual deliveries. High workload was defined for the 25% of maternities with the fewest midwives per annual deliveries. High workload was significantly associated with increased use of neuraxial analgesia in the sub sample of women who did not originally want it. The study was ranked as poor quality as midwife staffing levels were not the primary focus, and workload was derived from annual births.

Draper et al. (2017) (23) reported on the third perinatal confidential enquiry by MBRRACE-UK. The enquiry aimed to identify failures of care during labour, delivery and resuscitation that may have led to stillbirths and perinatal deaths. 104 cases were selected randomly from a sample covering 5.1% of all perinatal deaths in the UK in 2015. Cases were reviewed anonymously during panel meetings by healthcare staff including obstetricians, midwives, and neonatal nurses. Midwife workload was described in minimal detail as part of “staffing and capacity issues”. In 80% of deaths, improvements in care were identified that may have made a difference to outcomes. The panel concluded that staffing and capacity issues: were a problem in 21 cases with 17 related to the delivery suite; prevented women being admitted appropriately in 4 cases; delayed transfer between antenatal and midwifery units in 10 cases; and delayed induction of labour in 4 cases. Capacity issues were considered to be a contributing factor to poor outcomes in over half the cases where capacity issues were identified. The study was ranked as poor quality as the definition for midwife workload was not specific, and did not include a robust quantitative analysis e.g. regression.

Turner et al. (2022) conducted a study on the association between midwifery staffing levels and experiences of mothers on postnatal wards. The study was cross-sectional and used data from the National Maternity Survey in 2018 including 17,611 women from 129 organisations. The data was linked to hospital midwifery staffing numbers from NHS Workforce statistics. The relevant outcome variable was a survey of whether women experienced delays in discharge (yes/no). The staffing variable was the ratio of full-time equivalent midwives per 100 births and was an annual measure. Logistic regression results indicated that higher staffing levels were associated with less likelihood of women reporting delays in discharge (adjusted odds ratio 0.849, 95% CI 0.753 to 0.959, p < 0.008). The study was ranked as poor quality as the outcome variable was maternal self-report and had low response rates, in addition the maternal staffing variable was an annual measure.

Vanderlaan et al. (2023) conducted a cross-sectional study examining how midwifery density (i.e. workload) moderates the association between independent practice and pregnancy outcomes in USA. Data on births was obtained from the State Inpatient Databases from six different states and was combined with annual data on midwife to birth ratios from county level data in the Area Health Resource File. Midwife workload was categorised as no midwives, low midwife density (< 4.5 per 1,000 births), and high midwife density (>= 4.5 per 1,000 births). Multivariate logistic models were used assess the impact of states with independent midwifery practice (vs no independent midwifery practice) and midwifery density on C-section and preterm birth outcomes. High vs low midwifery density was associated with significantly lower C-section and preterm birth rates. There was a statistically significant interaction term between independent midwifery practice and midwifery density providing evidence of moderating effects. The study was ranked as fair quality as the midwife workload variable was an annual measure with an arbitrary cut-off (10%) used for categorisation.

Zbiri et al. (2018) (24) conducted a retrospective cohort study to identify factors that influence C-section delivery rates in France. Data was obtained from 11 maternity units in Yvelines from 2008 to 2014, including 24,000 C-section deliveries, split into urgent, elective, and intrapartum cases. Multiple staff workload variables were collected as the average number of obstetrician, anaesthesiologist, and midwife full time equivalents (FTEs) per 100 deliveries. Both staffing and deliveries were measured annually. Multilevel logistic regression analysis was conducted, with women nested within maternity units, and including hospital specific random effects. For elective cases, a higher ratio of midwife FTEs per deliveries was significantly associated with decreased C- sections, but there was no impact of obstetrician or anaesthesiologist staffing levels. For intrapartum C-section, a higher ratio of obstetrician FTEs per births was significantly associated with decreased C- sections, whereas higher midwife and anaesthesiologist staffing levels were associated with (non-significant) increases in C-sections. The study was ranked as poor quality as staffing and births were measured annually, and there was potential for multi collinearity in the regression equations.

Lyndon et al. (2022) conducted a cross-sectional study examining the relationship between nurse-reported staffing, missed nursing care, and exclusive breastmilk feeding during childbirth hospitalisation. The study linked 2018 Joint Commission PC-05 exclusive breastfeeding rates to survey data from 2,691 labour nurses across 184 hospitals in 29 US states. Nurse-reported staffing was measured based on perceived compliance with Association of Women’s Health, Obstetric, and Neonatal Nurses (AWHONN) staffing guidelines. Bivariate analyses showed a positive association between better staffing and exclusive breastfeeding rates, while missed nursing care, specifically missed skin-to-skin contact and breastfeeding initiation within one hour, was negatively associated with breastfeeding. Structural equation modelling suggested that some components of missed nursing care may mediate the relationship between staffing and breastfeeding rates. The study was rated as poor quality due to reliance on self-reported staffing data rather than objective measures that were aggregate over time (i.e. not obtained on the day of delivery).

Mercer (2016) conducted an observational cohort study of 101,120 pregnancies across 24 hospitals. Perinatal adverse outcomes included postpartum haemorrhage, low 5-minute Apgar scores, hypoxic-ischemic encephalopathy (HIE), shoulder dystocia, fetal trauma, and cord pH below 7.0. Multivariate regression did not identify any significant associations between nurse patient ratios (measured as total nursing hours per shift / births per shift/8 hours) and adverse perinatal outcomes. The study was published as a conference abstract and was therefore ranked as poor quality with high risk of bias due to the level of reporting.

Robertson (2021) investigated factors contributing to delays in the induction of labour (IOL) process and evaluated potential interventions to reduce these delays. Using retrospective data from a UK district general hospital (n=4,932 births in 2018), the study quantified delays in IOL and examined their association with maternity unit workload. Midwifery staffing shortfall was included as an explanatory, using the Birthrate Plus (BR+) tool which measures staff workload on wards at the time of delivery and accounts for caseload. Staffing shortfall was not found to be significantly associated with delays in IOL. Higher numbers of total labouring women (p=0.008) and planned IOL cases (p=0.009) were identified as significant predictors. The study was rated as fair quality but with a potentially high risk of bias as no covariates were included in the simple linear regression analysis.

Wilson et al. (2020) conducted a retrospective descriptive study examining the relationship between midwifery staffing and caesarean section rates among low-risk, full-term births. Using data from 11 hospitals in a large U.S. healthcare system, the study analysed the impact of midwifery hours per delivery on the likelihood of caesarean section rates, as well as induction and augmentation. Findings showed no significant relationship between a simple linear measure of staffing and caesarean section. However, when applying quadratic and piecewise regression models, an "optimal staffing" level of 31.2 nursing hours per delivery appeared to minimise caesarean section. In contrast, augmentation and induction rates were significantly associated with staffing levels (p < 0.01) even at optimal staffing levels. Despite the analysis appearing to be of high quality, the study was rated as fair quality due to limited reporting on exposure and outcome measurement. The study illustrates the importance of correct specification for staffing variables in regression equations.
